# A Tunable Hydrogel Platform Based on Platinum-Containing Polymeric Arsenicals

**DOI:** 10.1021/acs.chemmater.5c00184

**Published:** 2025-05-07

**Authors:** Alexandros Magiakos, Evelina Liarou, Spyridon Efstathiou, Andrea Dsouza, Chrystala Constantinidou, Marc Walker, Constantinos Methenitis, Paul Wilson

**Affiliations:** a Department of Chemistry, 2707 University of Warwick, Coventry CV4 7AL, U.K.; b Warwick Medical School, 2707 University of Warwick, Coventry CV4 7AL, U.K.; c Department of Physics, 2707 University of Warwick, Coventry CV4 7AL, U.K.; d Department of Chemistry, National and Kapodistrian University of Athens, Panepistimioupolis GR-15771, Greece

## Abstract

Platinum and arsenic (e.g., cisplatin, As_2_O_3_) have been used extensively in modern medicine due to their strong anticancer and antimicrobial activities. Here, polymeric arsenical scaffolds with varying As-functionalized acrylamide monomer (AsAm) composition are combined with Pt^II^ giving rise to hydrogels (**P1-Pt**–**P4-Pt**) with their properties being dependent on the AsAm content. The nature of the Pt–polymer interaction has been thoroughly investigated by ^1^H and ^195^Pt NMR spectroscopy as well as FT-IR, SEM, XPS, and potentiometric titration suggesting that cross-linking occurs primarily via coordination between oxygen atoms of the pendant arsenic acid group and Pt^II^, while secondary noncovalent interactions are proposed to provide further structural integrity and stability. Importantly, the hydrogels demonstrate potential self-healing properties, while the nature of the cross-linking promotes deep penetration of water into the loosely cross-linked networks. Finally, preliminary qualitative antimicrobial evaluation conducted via disk diffusion assay indicates that **P4-Pt** is active against Gram-negative (uropathogenic Escherichia coli CFT073 and Escherichia coli K12MG1655) and Gram-positive (Bacillus subtilis and Staphylococcus aureus USA 300 JE2) bacterial strains. Overall, the combination of polymeric arsenical scaffolds with Pt^II^ results in the formation of cross-linked networks generating soft, strong, and self-healing hydrogels with tunable stiffness and elasticity and preliminary indications of antimicrobial potential.

## Graphical abstract

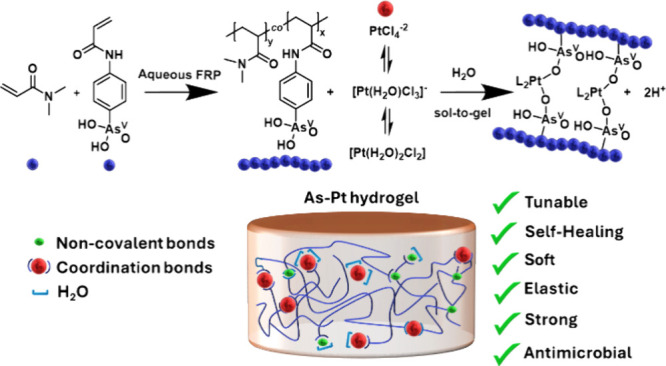

## Introduction

Polymer hydrogels are a versatile class of materials composed of 3D networks of cross-linked polymer scaffolds and varying amounts of water.^1^ The defining feature of polymer hydrogels, in contrast to other polymeric materials, is their high water content, typically exceeding 90%, thus providing access to a plethora of biomedical and nanotechnology-relevant applications.^2–9^ Typically, hydrogels can be fabricated via either a one-step method, in which multifunctional monomers are included in the original monomer feed, or a two-step approach, which involves the synthesis of polymer scaffolds containing reactive functional groups, followed by reaction of these scaffolds with multifunctional cross-linking reagents. It is the nature and density of the cross-linking process that ultimately determines the physical and mechanical properties of hydrogels.^10–12^ To increase the breadth of accessible mechanical properties, combinations of covalent and noncovalent cross-linking have been explored,^13–15^ while alternative supramolecular chemistries,^16,17^ notably host–guest,^18^ and metal–ligand (M–L)^19,20^ interactions have been developed. Metal–ligand interactions are particularly attractive for cross-linking polymer hydrogels^21^ because the local coordination sphere of the central metal ion (M^*n*+^) is often well-known based on the equivalent molecular complexes. The binding strengths can be tuned based on the choice of M and L, the oxidation state^22^ of the metal center, and the stoichiometry (M:L),^23^ while ligand exchange reactions in the coordination sphere of the central metal can elicit dynamic and self-healing properties.^24^ Importantly, these interactions are stronger than other noncovalent interactions and can approach the strength of covalent bonds when optimal M–L pairs are used.^25^ Moreover, while M–L interactions can be the driving force for cross-linking, they can also be supported by secondary noncovalent interactions to further influence and tune mechanical properties.^26,27^ Examples of M–L interactions exploited for the fabrication of polymeric hydrogels include (but not limited to); M^*n*+^—COO^–^,^28^ M^*n*+^—imidazole,^29^ M^*n*+^—(bi/ter-)­pyridine,^30^ M^*n*+^—catechol/pyrogallol.^31,32^

The distinct combination of platinum (Pt) and arsenic (As) containing materials exhibits significant interest, primarily owing their historical and ongoing use in medicine, from the use of As for thousands of years in Chinese medicine^33^ to the more recent development of As- and Pt-based chemotherapeutics.^34,35^ In the mid-20th century, the discovery that Pt inhibited cancer cell growth led to the development of Pt^II^ drugs, with cisplatin^36^ being approved by the FDA in 1978. Similarly, a renaissance in the use of (in)­organic As compounds was stimulated by the discovery that arsenic trioxide (As^III^) induced remission in patients with acute promyelocytic leukemia (APL).^37^ To date, Pt^II^ complexes cisplatin, carboplatin, oxaliplatin, and arsenic trioxide are the only nonradiative metal/metalloid complexes approved to treat cancer by the FDA.^38–40^ The antimicrobial activity of As^41^ and Pt^36^ complexes predates their clinical use as antitumor agents. More recently, aromatic organic arsenicals have been developed as antimicrobial additives for poultry litter,^42^ while cisplatin has been shown to have an extensive antimicrobial spectrum.^43^

Furthermore, Pt-complexes and (in)­organic arsenicals have exhibited diverse chemical reactivity that can be exploited in the context of polymeric materials,^44,45^ including protein/peptide–polymer bioconjugation^46^ as well as the fabrication of responsive polymeric arsenical nanoparticles^47–51^ and hydrogels,^52^ nontoxic and able to support cell culture. Likewise, Pt^II^-salts have been used as precursors for the synthesis of Pt^0^ nanoparticles (PtNPs), which have potential applications in catalysis, electronics, energy storage, and biomedical applications.^53^

In 2009, O’Halloran et al. reported a combination therapy for As and Pt drugs via coencapsulation into liposomes.^54^ New Pt^II^–As^III^ adducts were hypothesized to stabilize the liposomal formulation leading to attenuated toxicity toward hemotological and solid tumors. The structure and reactivity of the proposed Pt^II^–As^III^ complexes have been elaborated leading to the development of arsenoplatin-1,^55^ a new anticancer agent with greater bioactivity against solid tumor cell lines than the parent Pt^II^ and As^III^ drugs, exhibiting dual pharmacophore properties promoted by uptake of the complex followed by slow breakage of the Pt^II^–As^III^ bond.^56^ More recently, two discrete Pt^II/IV^–As^III^ clusters with Pt–As and Pt–O bonding have been reported.^57,58^ In the case of the Pt^IV^–As^III^ cluster, the Pt^IV^ ion was shown to be coordinated to two As_3_O_6_ units via coordination to oxygen (Pt^IV^–O ∼2.02 Å). Conversely, in the case of the Pt^II^–As^III^ cluster, the Pt^II^ ions were shown to be coordinated to four AsO_3_ ligands through two oxygen and two As^III^ atoms, respectively. Inspired by these results and based on our experience in developing polymeric arsenical (nano)­materials, we hypothesized that combining Pt^II^ with polymeric arsenical scaffolds could afford a new class of arsenoplatino (nano)­materials.

Aiming to exploit the synergy between As and Pt in a polymer hydrogel context, we synthesized a series of Pt-containing polymeric arsenical (As^V^) hydrogels. Thorough evaluation of the Pt–polymer interaction indicates that cross-linking is driven by coordination between oxygen atoms of the arsenic acid (AsO_3_H_2_, As^V^) and Pt^II^. Significantly, the kinetics of gel formation, the swelling ratio, and the physical and mechanical properties of the hydrogels can all be tuned based on the AsAm monomer content and [As]/[Pt], both of which influence the cross-linking density. Overall, we have demonstrated that Pt-containing polymeric arsenical hydrogels are soft but relatively strong, with self-healing potential and tunable stiffness and elasticity.

## Experimental Section

### Materials

Acryloyl chloride (≥97%), K_2_PtCl_4_, *N*,*N*-dimethylacrylamide (DMAm, con. 500 ppm monomethyl ether hydroquinone as an inhibitor), 4-amidobenzoic acid (≥99% *ReagentPlus*), dichloromethane anhydrous (≥99.8%, contains 40–150 ppm amylene as a stabilizer), ethyl acetate, *N,N*-dimethylformamide (DMF), and all deuterated solvents (D_2_O, CDCl_3_) were obtained from Sigma-Aldrich. Glacial acetic acid, HPLC grade water (DI), CuCl_2_, CuSO_4_, CaCl_2_, Na_2_HPO_4_, KH_2_PO_4_, NaClO_4_, NaCl, NaOH, K_2_CO_3_, H_3_PO_4_ (≤99%), KOH pellets, PBS 1X, pH 7.4 (Gibco), methanol, con. HCl (37%), and H_2_SO_4_ (98%) were purchased from Fisher Scientific. Chloroform and tetrahydrofuran were received from VWR international LLC. 2,2′-Azobis­[2-(2-imidazolin-2-yl)­propane] dihydrochloride (VA-044) was purchased from Wako Chemicals. Alfa Aesar provided *p*-arsanilic acid (≥98%). (Propionic acid)­yl butyl trithio-carbonate (PABTC) was synthesized according to formerly reported synthetic process.^59^ An Ultrapure milli-Q Type I water system was purchased from Merck. A Lucky Reptile Herp Nursery II Incubator employed with an H–B Instrument Durac Electronic Thermometer-Hygrometer was bought by Fisher Scientific. For pH measurements other than titration, a Mettler FiveGo F2 pH/mV Meter Field equipped with a LE438 IP67 Sensor Mettler Toledo (0–14 range) standardized with certified buffer solutions of pH = 4 and 7 provided from the same company was utilized. Uropathogenic Escherichia coli (E. coli) CFT073 and E. coli K12 MG1655 were obtained from Dr. Chrystala Constantinidou (Warwick Medical School, University of Warwick). Staphylococcus aureus (S. aureus) *USA 300 JE2* and Bacillus subtilis (B. subtilis) were obtained from Prof. Meera Unnikrishnan (Warwick Medical School, University of Warwick). Lysogeny Broth (LB), Tryptic Soya Broth (TSB), and Mueller Hinton Broth-II (MHB-II) were purchased from Merck Millipore. Bacteriological agar was purchased from Becton Dickinson & Company Difco. All reagents were used without further purification unless otherwise stated. Before any experiment, the hydrogels were washed three times with milli-Q water to remove excess unreacted materials stuck on the surface and dried thoroughly with paper tissue.

### General Procedure for the Synthesis of 4-(*N*-Acrylamido)­phenylarsonic Acid (AsAm)^50^

Potassium hydroxide (5.1 g, 91.8 mmol) was dissolved in deionized water (125 mL). The mixture of *p-*arsanilic acid (10.0 g, 46.1 mmol) and Na_2_CO_3_ (14.5 g, 137.1 mmol) was added portionwise to the KOH to afford dissolution. Acryloyl chloride (5.6 mL, 68.9 mmol) in dichloromethane (25 mL) was quickly added into the *p*-arsanilic acid solution at 0 °C and stirred for 15 min. The aqueous phase was collected and carefully acidified to pH 1 through the addition of H_2_SO_4_ (98%), which resulted in precipitation of the product. The precipitate was collected by filtration, washed with cold water, and dried in the vacuum oven overnight to afford the title compound as a white solid. (11.43 g, 49.9 mmol, α = 91,8%). ^1^H NMR (D_2_O/NaOH 1 M, 400 MHz): δ_H_(ppm) = 7.55 (2H, d, J_HH_ = 8.07 Hz, AsCC**H**), 7.11 (2H, d, J_HH_ = 7.95 Hz CC**H**), 6.25 (1H, m, J_HH_ = 10.51, 6.85 Hz, CC**H**), 5.95 (1H, d, J_HH_ = 17.36 Hz, CH**H**), 5.53 (1H, d, J_HH_ = 10.51 Hz, C**H**H); ^13^C NMR: δ_C_(ppm) = 123.12 (**Ar**), 124.29 (H_2_ **C**=C−), 130.53 (**Ar**) 130.62 (H_2_C=**C**−), 133.34 (**Ar**), 134.73 (**Ar**), 168.72 (−**C**=O); FT-IR (ATR, cm^–1^): 3440, 3296, 3189, 3110, 3060, 2780, 1673 (vC=O), 1640, 1622, 1589, 1532, 1296, 906 (vAsO_*x*_), 834 (vAsO_*x*_), 777.

### General Procedure for the Synthesis of 4-Acrylamidobenzoic Acid^60^

To a stirred solution of 4-aminobenzoic acid (2.74 g, 20 mmol) and K_2_CO_3_ (6.9 g, 50 mmol) in 20 mL dry CH_2_Cl_2_ was added acryloyl chloride (1,95 mL, 24 mmol) slowly under an argon atmosphere at 0 °C. The resulting mixture was stirred at 0 °C for 1 h, transferred to room temperature overnight, followed by addition of water (40 mL), and then extracted with ethyl acetate (3 × 20 mL). The aqueous layers were combined, and the pH was adjusted with HCl (37%) at 1 to afford precipitation of the product. The precipitate was collected by filtration, washed with cold water, and dried in the vacuum oven overnight to afford the title compound as a white solid (2,79 g, 23.2 mmol, α = 73%). ^1^H NMR (D_2_O/NaOH 1 M, 400 MHz): δ_H_(ppm) = 7.76 (2H, d, J_HH_ = 8.56 Hz, COCC**H**), 7.26 (2H, d, J_HH_ = 8.44 Hz, CC**H**), 6.33 (1H, m, J_HH_ = 10.39, 6.72 Hz, CC**H**), 6.12 (1H, d, J_HH_ = 17.24 Hz, CH**H**), 5.67 (1H, d, J_HH_ = 10.52 Hz, C**H**H); FT-IR (ATR, cm^–1^): 3405, 3305, 3000, 1694 (vC=O carboxylic), 1663 (vC=O Amide I), 1630, 1593, 1522, 1408, 1316, 1291, 767.

### General Procedure for Polymer Synthesis via Free Radical Polymerization

AsAm (or 4-acrylamidobenzoic acid) was dissolved in water with 1 equiv of sodium hydroxide and added to a cooled aqueous solution of DMAm (1 M) containing VA-044 (0.01 equiv relative to the total monomer content). In the case of PDMAm, no comonomer was added. The solution was degassed for 15 min and then heated at 90 °C for 2 h. For the synthesis of low-molecular-weight scaffolds, 0.01 equiv of the chain transfer agent PABTC (with respect to total monomer content) was also added to the reaction mixture. The full consumption of the monomer was confirmed by ^1^H NMR before dialysis (SpectrumLabs PreWet Laboratory dialysis membrane 6000 Da) and freeze-drying directly to yield white solids (<99%). **P1**–**P4**: ^1^H NMR (D_2_O, 400 MHz): δ_H_(ppm) = 7.20–7.82 (**ArH**), 2.3–3.2 (−NC**H**_3_, −C**H**– backbone), 1.0–1.9 (−CC**H**_2_C– backbone); FT-IR (ATR, cm^–1^): 3405, 3236, 2930, 1672 (vC=O Amide I—AsAm), 1608 (vC=O Amide I—DMAm), 1532 (bN–H Amide II—AsAm), 1254 (vC–N Amide III—DMAm), 880/862 (vAsO_*x*_), 719 (b, vAsOH/p-aromatic bending).

### General Procedure for Arsenoplatinohydrogel Synthesis

For 10 wt % gels, 100 mg of each polymer scaffold (**P1**–**P4**) was initially dissolved in 1 mL of DI water before adding 1 eq. to AsAm groups, dipotassium tetrachloroplatinate (II) (K_2_PtCl_4_). The solution was left to incubate at 50 °C overnight, resulting in a successful clear gel formation (black-orange color). The freshly prepared hydrogels were directly utilized (within 3–5 days and stored at −5 °C) for all experiments. **P4-Pt**: ^1^H NMR (D_2_O, 400 MHz): δ_H_(ppm) = 7.28–7.86 (**ArH**), 2.3–3.2 (−NC**H**_3_, −C**H**– backbone), 1.0–1.9 (−CC**H**_2_C– backbone); FT-IR (ATR, cm^–1^): 3405, 3236, 2930, 1669 (vC=O Amide I—AsAm), 1608 (vC=O Amide I—DMAm), 1532 (bN–H Amide II—AsAm), 1254 (vC–N Amide III—DMAm), 903 (vAs=O), 832 (vAs–O–Pt) 728 (vAs–OH/p-aromatic bending).

### Nuclear Magnetic Resonance (^1^H NMR, ^13^C NMR, and ^195^Pt NMR)

All spectra were recorded on Bruker DPX-400 and DPX-600 MHz spectrometers in deuterated chloroform (CDCl_3_) or deuterium oxide (D_2_O). Bruker triple resonance observer TXO CryoProbe was used to acquire ^195^Pt NMR. Chemical shifts are reported in ppm relative to the internal standard tetramethylsilane (TMS). ^195^Pt NMR chemical shifts were referenced indirectly to TMS in the ^1^H NMR spectrum such that K_2_^195^PtCl_6_ in D_2_O would resonate at 0.0 ppm (δ = −1617 ppm (s, 1Pt) for K_2_PtCl_4_). ACDLABS software was used to analyze the data. All hydrogel samples were prepared *in situ* within standard NMR tubes prior to each measurement.

### Infrared Spectroscopy (IR)

All infrared spectra were recorded using a Bruker ALPHA II or Agilent Cary 630 Fourier-transform infrared spectrometer (FT-IR), scanning between wavenumbers of 500 and 4000 cm^–1^. Freshly prepared hydrogels (10 wt %) were freeze-dried prior to each measurement.

### UV–vis (Absorption) Spectroscopy

UV–vis spectra were recorded on an Agilent Technologies Cary 60 UV–vis spectrometer in the range 200–600 nm, equipped with a Quantum Northwest temperature-controlled cuvette holder to adjust the temperature during kinetic studies. For all measurements, quartz cuvettes (purchased from HELLMA) with 1 or 0.1 cm optical lengths were used. All gels were prepared inside the cuvette.

### Size Exclusion Chromatography (SEC)

AqueousSEC: Agilent PL50 instrument was equipped with a differential refractive index (DRI) detector. The system was equipped with a PL Aquagel OH 60 column (300 mm × 7.5 mm) and an 8 μm aquagel guard column. The eluent was 80% NaNO_3_ in milli-Q water at 0.1 M and 20% methanol, and the flow rate was 1 mL min^–1^ at 35 °C. PEG/PEO standards in the range of 190–1,100,000 g/mol (Agilent *EasiVials*) were used for calibration. Prior to injection of the samples, they were filtered through a GVHP membrane with 0.45 mm pore size before injection. DMF SEC: Agilent Infinity II MDS instrument equipped with differential refractive index (DRI), viscometry (VS), dual angle light scatter (LS), and variable wavelength UV detectors. The system was equipped with 2 × PLgel Mixed D columns (300 × 7.5 mm) and a PLgel 5 μm guard column, made from poly­(styrene divinylbenzene). The eluent was DMF with 0.01% NH_4_BF_4_ additive and the flow rate 1 mL min^–1^ at 50 °C. Poly­(methyl methacrylate) standards (Agilent *EasiVials*) were used for calibration in the range of 550–955,000 g mol^–1^. Prior to injection, the samples were filtered through a nylon membrane with a pore size of 0.22 μm. Respectively, experimental molar mass (*M*_*n*_, SEC) and dispersity (*Đ*) values of all synthesized polymers were determined by conventional calibration using Agilent GPC/SEC software.

### Potentiometric Titrations

pHmetric titrations were performed in ambient temperature using an automatic Mettler Toledo compact G20S titrator equipped with a pH electrode Mettler Toledo DGi115-SC (0–14 range) standardized with certified buffer solutions of pH = 4 and 7 provided from the same company. The electrode was stored and immersed in InLab Storage solution of KCl 3 mol/L. Calibration curves with 0.1 M acetic acid (prepared by glacial acetic acid in DI water) were performed prior to every experiment. Error was always less than 0.3%. NaOH (0.1 M) obtained from Sigma-Aldrich was used as a titrant in all cases. Fully protonated scaffolds were used for potentiometric titrations. DI water or MeOH/H_2_O mixture was used as solvent in sample preparation. Where necessary, reported results were converted to aqueous solutions according to recommended by IUPAC, method.^61^ All experiments were performed at least twice in 50 mL of sample solutions.

### Swelling Studies

Freshly prepared hydrogels (10 wt %) were immersed in DI water (DI) or in different pH phosphate buffer solutions for 7 days at 25 °C. A solvent was added occasionally if needed, to keep the volume constant (8 mL). All hydrogels were periodically removed from solution, excess water was removed using tissue, and the weight was recorded. The swelling ratio was calculated using the formula:

$$\mathrm{Swelling}\;\mathrm{ratio}\;(\%)=\frac{W_{s}-W_{o}}{W_{o}}\times 100$$ 1

where *W*_s_ is the weight of the swollen gel and *W*_o_ is the weight of the initial wet weight before immersed (directly after preparation). Unless stated otherwise, all tests were performed in triplicate, and results are presented with the standard deviation bars.

### Scanning Electron Microscopy (SEM) and Energy-Dispersive X-ray (EDX) Spectroscopy

Scanning electron microscopy was performed using a ZEISS Gemini SEM field-emission scanning electron microscope and ZEISS Supra SEM. Best results were obtained when using the InLens detector with ∼3.5 mm working distance, 20 (Gemini) or 30 (Supra) μm aperture, and 1–7 kV acceleration voltage, with respect to sample tolerance. EDX spectroscopy and elemental analysis were performed on a Gemini instrument through an SDD EDX detector. Washed with H_2_O and then freeze-dried gels were casted on carbon tabs (9 mm) attached to aluminum specimen stubs. For the improvement of the sample imaging, gold (Au) sputter coating was applied for 10 s prior imaging. Elemental analysis was carried out during SEM imaging on the same samples using an Oxford Instruments X-Max 150 large area SDD electron dispersive X-ray analysis (EDX) detector.

### Compression Tests

All tests were carried out by utilizing a Shimadzu EZ-LX Universal Testing Instrument equipped with a 500 N load cell. Hydrogels were prepared in molds then cut to size before mounting (approximately 15 mm width, 5 mm thickness). The extension was zeroed prior to starting data collection when the top plate was starting to load the sample (preload force of ∼0.09 N), and the compression velocity was set at 5 mm min^–1^. Each hydrogel was compressed to a fixed load of 500 N to generate representative stress/strain curves and quantify gel stiffness (Young’s Modulus, *Y*). **P1-Pt**–**P4-Pt** gels were retested in three consecutive cycles to check relaxation and elasticity. The tests were repeated at least 3 times for each sample. All data were initially processed in Bluehill software and then plotted using OriginLab software.

### Rheology

Rheological measurements were performed in an Anton Paar MCR 302 rheometer equipped with a parallel plate configuration (25 mm diameter, 1 mL scale). The normal force was kept constant at 5 N during measurements, and all measurements were performed at room temperature. For the frequency sweep, constant strain (10.0%) was applied and frequency was ramped logarithmically from 1 to 100 rad s^–1^. Amplitude sweeps were performed at an oscillating frequency of 10 rad/s. Cyclic strain experiments were conducted to examine the self-healing behavior. For the tests, hydrogels were cut in half and recombined for 1 h in a humidity chamber before amplitude sweeps were remeasured. The tests were repeated at least 3 times for each sample. The data were analyzed using RheoCompass software and plotted using OriginLab software.

### X-ray Photoelectron Spectroscopy (XPS)

Measurements were performed on a SiO_2_ sample bar (substrate) using a Kratos AXIS Ultra DLD in an ultrahigh vacuum system with a base pressure below 1 × 10^–10^. The spectrometer had a monochromatic Al K_α_ X-ray source with an energy of 1486.7 eV operating at 15 kV, 10 mA (150 W) for all data acquisitions, and the photoelectrons were detected at a 90° takeoff angle. The overall information depth was ∼10 nm (3λ, where λ is the electron free path), and core level spectra were recorded using a pass energy of 20 eV (resolution ∼0.4 eV). The binding energies were charge corrected to 285.0 eV for aliphatic C 1s. The surface was flooded with a beam of low energy electrons throughout the experiment to neutralize the surface charging of organoarsenicals. High-resolution spectra were resolved into mixed Gaussian–Lorentzian (Voigt) line shapes using a least-squares fitting program (CasaXPS package using Shirley background). Component energies, number of peaks, and peak widths were fixed initially (full width at half-maximum (fwhm) values of 1.0, 1.6, 1.2, 1.08, and 1.1 for all C s, O s, N s, Pt 4f, and As 3d, respectively) and refinement was performed for peak heights only. In a final optimization cycle, component energies and peak widths were also refined, and these changed by less than 1.0%. Peak fit results were imported into OriginLab Pro graph software for displaying the modeled data, and the intensities were normalized when stated. For compositional analysis, the analyzer transmission function has been determined using clean metallic foils to determine the detection efficiency across the full binding energy range. The samples measured were washed thoroughly with milli-Q water to remove surface impurities before lyophilization and utilization for XPS measurements.

All data were analyzed with OriginLab Pro graph software, unless stated otherwise. The samples for XPS and ^195^Pt NMR analysis were initially fabricated at 25 °C to avoid platinum hydroxide formation.

### Antibiotic Diffusion Assay

The antibacterial activity of hydrogels was tested against Gram-negative (uropathogenic E. coli CFT073 (*UPEC*), E. coli K12 MG1655) and Gram-positive (S. aureus USA 300 JE2, B. subtilis). Bacteria were revived from glycerol stocks and streaked on LB agar (for E. coli and B. subtilis) and Tryptic Soya Agar (TSA) (for S. aureus). Single bacterial colonies were picked from each agar plate and inoculated into LB (for E. coli and B. subtilis) and TSB (for S. aureus) for an overnight primary culture. Subsequently, 100 μL of *UPEC*, E. coli K12 MG1655, and B. subtilis were plated onto LB agar plates, while S. aureus was plated onto TSB agar plates, each as lawn cultures. The assay included two control samples: a **P4** polymer scaffold aqueous solution at a concentration of 100 mg/mL and a platinum (Pt^II^) aqueous solution of 30 mM K_2_PtCl_4_, matching the Pt^II^ concentration in the gel 10 wt % matrix. The test samples comprised **P4-Pt** freshly prepared hydrogels to evaluate their effect on bacterial growth. They were cut in 0.4 cm diameter and placed on plates containing lawn bacterial growth. After hydrogel introduction on agar plates, the plates were incubated at 37 °C for 18 h followed by measurement of zone of bacterial inhibition.

## Results and Discussion

### Synthesis of Polymer Scaffolds

For the incorporation of an As^V^ motif into target polymer scaffolds, 4-(*N*-acrylamido)­phenylarsonic acid (AsAm) was synthesized and isolated with high spectroscopic purity (Figure S1A–C). Subsequently, high-molecular-weight copolymers of AsAm and *N*,*N*-dimethylacrylamide (DMAm) with AsAm contents of 2 (**P1**), 4 (**P2**), 6 (**P3**), and 8 mol % (**P4**) were synthesized by aqueous free radical polymerization (FRP) at 90 °C using 2,2′-azobis­[2-(2-imidazolin-2-yl)­propane] dihydrochloride (VA-044) as the initiator (Table 1).^52^ After purification, the composition of AsAm incorporated into each scaffold was quantified by ^1^H NMR spectroscopy (Figure S2A); the presence of the As^V^ motif was further verified by detailed Fourier transfer infrared (FT-IR) spectroscopy (Figure S2B), while size exclusion chromatography (SEC) was used for the analysis of the polymer scaffolds’ molecular weight and dispersity values (Figure S3). A homo polymer of DMAm (PDMAm, **P5**) of comparable molecular weight was also synthesized by FRP as a control for hydrogel fabrication (Figure S4A,B).

**Table 1:**
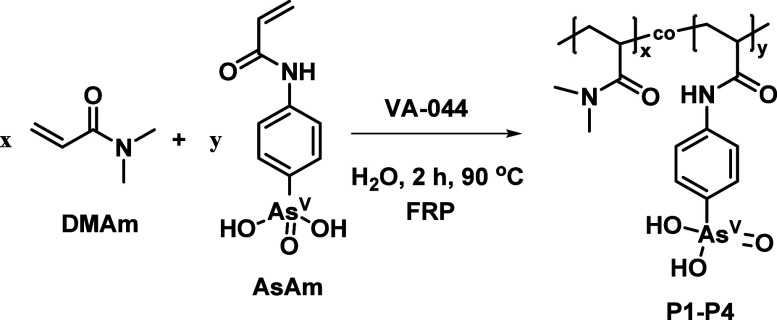
Polymeric Arsenical Scaffolds P­(AsAm_*x*_-DMAm_*y*_) Synthesized by Free Radical Polymerization

**Polymer**	***M*_n,SEC_** ^a^ **/g mol** ^**–1**^	***M*_w,SEC_** ^a^ **/g mol** ^**–1**^	***Đ*_m_** ^a^
P(DMAm_0.98_-AsAm_0.02_)**/P1**	88,000	355,000	4.00
P(DMAm_0.96_-AsAm_0.04_)**/P2**	86,000	359,000	4.16
P(DMAm_0.94_-AsAm_0.06_)**/P3**	122,000	396,000	3.25
P(DMAm_0.92_-AsAm_0.08_)**/P4**	56,000	312,000	5.53
P(DMAm)**/P5**	50,000^b^	270,000^b^	5.37^b^

a Aqueous SEC.

b DMF SEC.

### Fabrication of As^V^–Pt^II^ Hydrogels

Hydrogels were generated through a simple mixing of polymer scaffolds **P1**–**P4** with K_2_PtCl_4_ in water at neutral pH. Initially, **P4** (10 wt %) was dissolved in an aqueous solution of K_2_PtCl_4_ ([As]/[Pt] = 1) and left at room temperature. An inversion test after 48 h revealed formation of a brown, transparent gel (Figure 1A). Subsequently, **P1**–**P4** (10 wt %) were separately dissolved in aqueous solutions of K_2_PtCl_4_ ([As]/[Pt] = 1) and heated at 50 °C overnight, resulting in gel formation (Figure S5). When **P4** with the highest mole fraction of AsAm (8 mol %) was dissolved in DI water and heated at 50 °C in the absence of K_2_PtCl_4_, no gelation was observed (Figure 1B), while the same result was obtained when **P5** (without AsAm content) was used (Figure 1C). These observations indicated that interactions between the pendent As^V^ group of AsAm and Pt^II^ were responsible for the formation of the hydrogel network.

**Figure 1:**
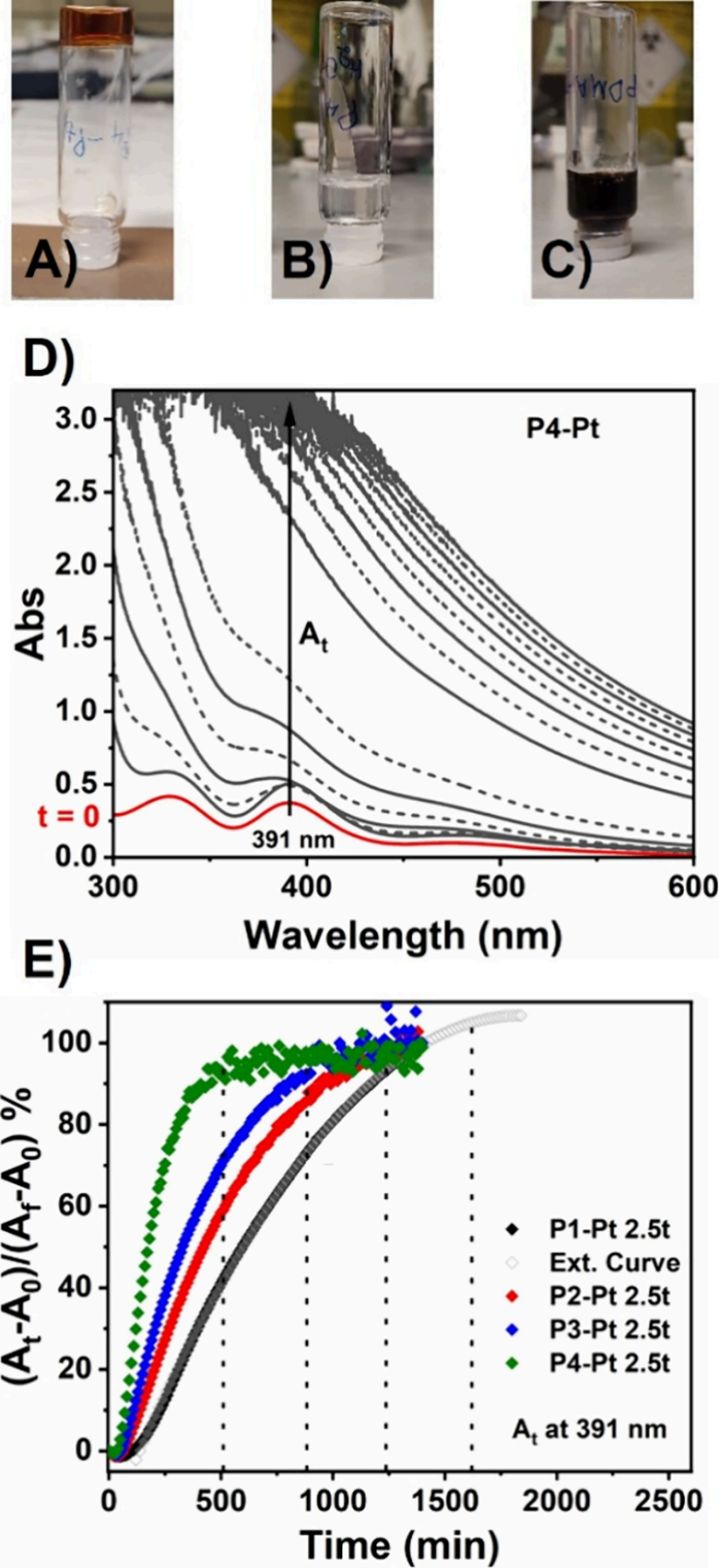
Inversion test for: (A) **P4** in the presence of K_2_PtCl_4_ (**P4** = 10 wt %, [As]:[Pt] = 1:1) formed at room temperature resulting in formation of **P4**-**Pt**, (B) **P4** in the absence of K_2_PtCl_4_ (**P4** = 10 wt %), and (C) **P5** in the presence of K_2_PtCl_4_ (**P5** = 10 wt %, [As]/[Pt] = 1 w.r.t. **P4**); (D) representative UV–vis spectroscopy of **P4** in the presence of K_2_PtCl_4_ (**P4** = 2.5 wt %, [As]/[Pt] = 1, 50 °C, 1 cm path length, 24 h, H_2_O) following changes to the peak at λ = 391 nm to monitor gel formation; and (E) gel formation as a function of time for **P1**–**P4** in the presence of K_2_PtCl_4_ (**P1**–**P4** = 2.5 wt %, [As]/[Pt] = 1) following changes to the peak at λ = 391 nm monitored at 10 min intervals. Structures of **P1**–**P4** can be found in Table 1, and a proposed reaction scheme for gel formation is presented in Scheme 1.

The measurement of absorption is a useful tool to monitor the relative kinetics of gelation processes due to the commonly observed increase in the turbidity of samples as they gel.^62^ Initially, UV–vis spectroscopy of the polymer scaffolds revealed a λ_max_ = 254 nm and no absorption at λ > 300 nm (Figure S6). Conveniently, analysis of K_2_PtCl_4_ exhibited low intensity peaks at λ = 329, 391, and 478 nm attributed to d-d transitions of the d^8^ D_4h_ symmetry system.^63^ Monitoring of the K_2_PtCl_4_ peaks revealed that the intensity of the peak at λ = 391 nm remained unaltered over a period of 48 h, with a small blue shift attributed to ligand exchange (Cl^–^ → H_2_O) in the cis position (Figure S7).^64^ At 50 °C, the same blue shift was observed with a concomitant increase in absorbance due to deprotonation of the Pt-coordinated water molecules resulting in the formation of darker Pt­(OH)_2_ species (Figure S8A,B).^65^ In the presence of the polymer scaffold (**P4**), potentiometric titration indicated that the formation of Pt­(OH)_2_ species was negligible under the conditions employed for hydrogel formation (*vide infra*) indicating that the peak at λ = 391 nm was suitable for monitoring gel formation (Figure 1D).

The absorption spectra of **P1**–**P4** (2.5 wt %) in aqueous K_2_PtCl_4_ ([As]/[Pt] = 1) were then monitored at 10 min intervals for 24 h at 50 °C. Each polymer exhibited a lag phase (*t*_lag_), during which absorbance at λ = 391 nm was unchanged (*A*_t_ = *A*_0_). The duration of the lag phase (*t*_lag_) was found to decrease with an increasing AsAm content (**P1** > **P2** > **P3** > **P4**, Figure S9A). The subsequent increase in absorption as a function of time (*A*_t_ > *A*_0_) was attributed to the increasing interactions between the polymers and Pt^II^ in solution. The increase plateaued (*A*_f_) when the majority of K_2_PtCl_4_ had been consumed to afford complete gelation (Figure 1E). Notably, the time required to reach complete gelation decreased with increasing AsAm content (Figure S9B), with the polymer scaffold containing the highest mole fraction of AsAm (**P4**, 8 mol %) reaching *A*_f_ after 502 min. The time increased to 900 min for **P3** and 1220 min for **P2**. The polymer scaffold with the lowest AsAm mole fraction (**P1**, 2 mol %) did not reach *A*_f_ over the course of the experiment, with the inversion test confirming complete gelation had not occurred. Extrapolation of the data suggested that *A*_f_ would be reached after 1690 min (Ext. curve, Figure 1E).

### Understanding the Pt^II^–Polymer Interaction

To explore the chemical interactions between the Pt^II^ and As^V^ moieties and understand the origin of cross-linking, the hydrogel derived from the polymer scaffold with the highest AsAm content (**P4-Pt**) was examined. ^1^H NMR spectroscopy of **P4** and **P4-Pt** showed no shift for the proton environments associated with the polymer backbone (δ_H_ = 1.00–2.75 ppm) and the DMAm side chains (δ_H_ = 2.75–3.25 ppm) (Figure 2A). Conversely, the protons associated with the 1,4-substituted aromatic ring of the AsAm side chain showed discrete downfield shifts from δ_H_ = 7.67/7.78 ppm to 7.71/7.79 ppm. This was attributed to interactions between the arsenic acid side chain of AsAm and Pt^II^ leading to subtle changes in the electronic current densities within the aromatic ring. To confirm the nonparticipation of the amide functional group (of DMAm) in interactions with Pt^II^, ^1^H NMR spectra of **P5** and **P5-Pt** were acquired and showed no shift in the respective proton environments (Figure S10).

**Figure 2:**
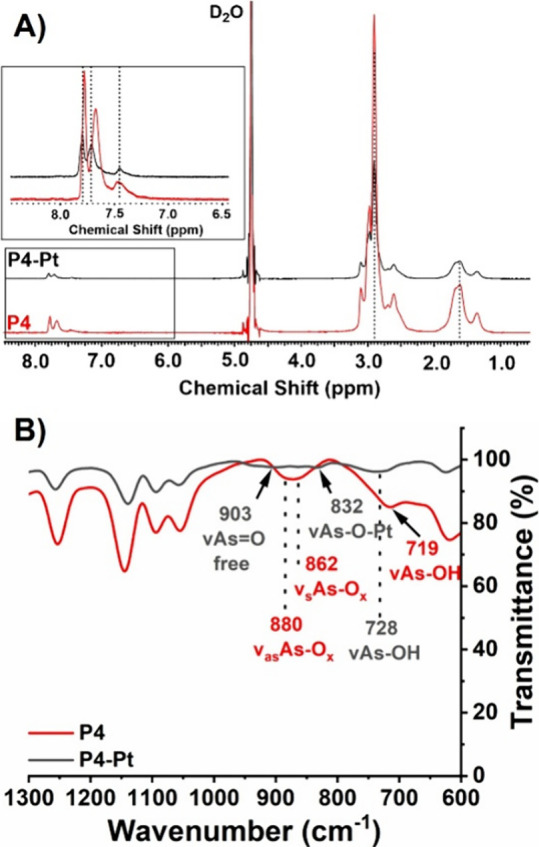
(A) ^1^H NMR spectra of **P4** (red) and **P4**–**Pt** (green) showing no changes to the polymer backbone and DMAm side chain peaks but a small shift in the side chain peaks of AsAm upon addition of K_2_PtCl_4_, and (B) FT-IR spectra of **P4** (red) and **P4**–**Pt** (green) showing changes to the AsAm functional group upon addition of K_2_PtCl_4_. This supports the proposal that As^V^–Pt^II^ interactions, rather than the interaction with the amide functionality present, promote the gelation process.

Comparison of the FT-IR spectra of **P4** and **P4-Pt** revealed a splitting of the broad νAs–O_*x*_ peak at 860–880 cm^–1^ in **P4** into two lower intensity peaks at 903 and 832 cm^–1^, which were assigned to the stretching vibration of As–O (Figure 2B). The higher frequency peak (903 cm^–1^) was assigned to the As=O bond. The lower frequency peak (832 cm^–1^) was assigned to the coordinated As–O–Pt stretching vibration. A third resonance in this region at 728 cm^–1^ was attributed to changes in the As–OH bond energies upon interaction with Pt^II^. FT-IR analysis of **P4** and **P4-Pt** showed no change in the amide carbonyl region (1608 cm^–1^) providing further evidence for nonparticipation in the cross-linking process (Figure S11).

Influenced by the exceptional anticancer activity of arsenoplatin-1 in which the Pt–As core combines the synergistic activity of arsenous acid (As^III^) and Pt^II^ based drugs, the formulation of **P4-Pt** was monitored by ^195^Pt NMR spectroscopy. Arsenoplatins have been shown to contain a formal Pt^II^–As^III^ covalent bond resulting in ^195^Pt resonances in the region of δ_Pt_ = −3400 to δ_Pt_ = −3700 pmm.^56,66^ When **P4** and K_2_PtCl_4_ were dissolved in D_2_O and stored at room temperature in the dark to afford formation of **P4-Pt**, no distinctive peak was observed indicating that a formal Pt–As bond was not present (Figure S12). However, the pendent group of AsAm within the polymer scaffolds used contains arsenic acid (As^V^) functional groups rather than arsenous acid (As^III^) present in arsenoplatin-1, which confirms that the covalent bond formation between Pt–As is not the driving force for hydrogel formation. The region between δ_Pt_ = −1000 to δ_Pt_ = −1650 ppm was examined revealing peaks at −1617 and −1184 ppm, which were assigned to K_2_PtCl_4_ and [Pt­(H_2_O)­Cl_3_]^−^, respectively,^67^ confined within the hydrogel network (Figure S13). It is known that substitution of PtCl_4_^–2^ with oxo-ligands such as H_2_O, Me_2_S=O, and OSO_3_^2–^ leads to a downfield shift due of ^195^Pt resonance.^68^ For example, when H_2_O molecules replace the chloride ions in cisplatin, a downfield shift of about 300 ppm was observed.^69^ This was verified by ^195^Pt NMR of K_2_PtCl_4_ (30 mM) in D_2_O, which revealed equivalent peaks with a better signal-to-noise ratio than observed in **P4-Pt** (Figure S14). Importantly, the absence of any signals that could be confidently assigned to chelating *O*- and *N-*substitution of the Pt-center provided further evidence that interactions with the pendant amide groups were not responsible for hydrogel formation.^70,71^

While ^195^Pt NMR was useful to rule out Pt–As bond formation and amide coordination, it did not conclusively reveal the nature of the Pt^II^–polymer interaction leading to hydrogel formation. Considering the acidity of the arsenic acid pendent group, **P1**–**P4** constitutes a polyelectrolyte system. As polyelectrolyte-metal interactions are pH sensitive, potentiometric analysis of a low-molecular-weight fully protonated analogue of **P4** (**P4′** 8 mol % AsAm, *M*_n_ = 17,000 g·mol^–1^, *Đ*_m_ = 2.37, Figure S15A–C) was performed. This was to avoid very high viscosity and gelation during the experiment following the addition of Pt^II^. Initially, the potentiometric titration of AsAm revealed p*K*_a_ values of p*K*_a1_ = 2.96 and p*K*_a2_ = 7.46 (Figure S16). In the case of **P4′**, the p*K*_a_ was determined at a degree of dissociation α = 0.5 (p*K*_1/2_) using the extended and modified Henderson–Hasselbach equation (see the Supporting Information);^72^

$$\mathrm{pH}=pK_{1/2}-n\log[\frac{1-a}{a}]$$ 2

The titration curve of **P4′** in water revealed 2 inflection points, which when plotted using eq 2 revealed an increase in p*K*_a_ values compared to AsAm with p*K*_a1_ = 3.88 and p*K*_a2_ = 8.51 (Figure 3A). Considering the influence of ionic strength on the *K*_a_,^73^ the titration of **P4′** was repeated at higher ionic strength (0.1 M NaClO_4_). There was a minimal change in the titration curve and apparent p*K*_a_ values calculated (Figure 3B), which is likely due to the relatively low mole fraction of AsAm (8 mol %) present in the polymer composition. The linearity of the Henderson–Hasselbach plots indicates a minimal conformational change across all α values. To exclude any possible pH influence on the pDMAm homopolymer **P5**, its titration curve was acquired showing the expected absence of any equivalence points (Figure S17). Next, titrations of **P4′** were repeated in the presence of K_2_PtCl_4_ with [As]/[Pt] = 1–4.

**Figure 3:**
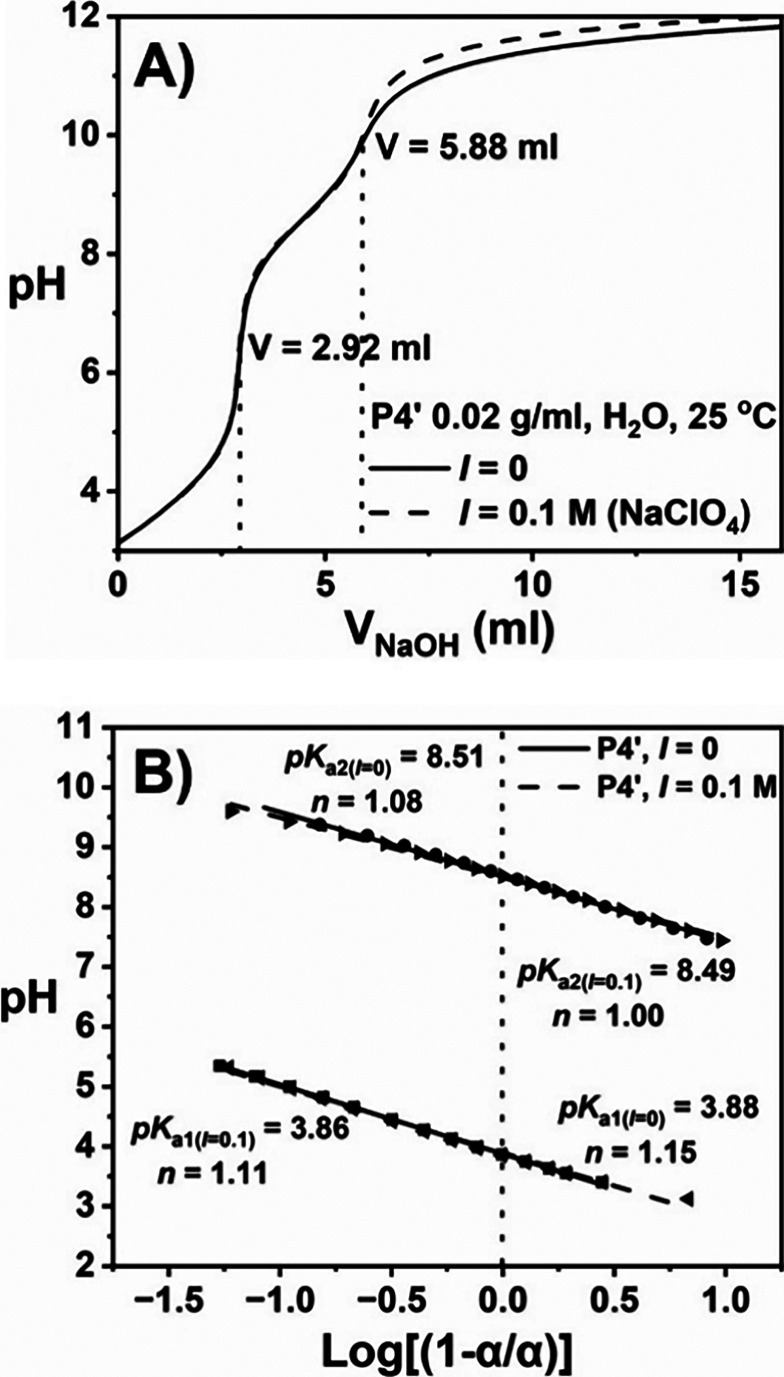
(A) Potentiometric titration curve of **P4′** in H_2_O (solid line) and NaClO_4_ (0.1 M, dashed); (B) corresponding p*K*_a_ values of p*K*_a1_ = 3.88 and p*K*_a2_ = 8.51 determined by relating the pH to the degree of dissociation (α = 0.5 (p*K*_1/2_)) using the extended and modified Henderson–Hasselbach equation.

Examination of the first part of the curves revealed a decrease in pH with increasing [Pt], i.e., [As]/[Pt] = 4 > 2 > 1 in DI water (Figure 4A) and *I* = 0.1 M (Figure S18). This decrease displays no significant difference in the presence or absence of added salt, as the apparent ionization constant (p*K*_a_) of the polymer in both conditions is similar (Figure 3B). Closer inspection of the titration curves indicated that the pH decreased with increasing neutralization (increase in α), while p*K*_a1_ values at α = 0.5 were lower at higher [Pt] (Figure 4B,C and Table S1). Using α as an indirect measure of reaction progress, it was hypothesized that while monovalent Na^+^ behaves as an inert counterion, Pt^II^ undergoes complexation with the pendent arsenic acid groups resulting in the release of H^+^ into solution (Scheme 1).^74^ This causes a decrease in pH and ultimately leads to cross-linking and gelation when enough interactions occur between different polymer chains. This was supported by the observation that hydrogels were not formed in the presence of either 0.1 M NaClO_4_ or NaCl, while the addition of Pt^II^ to a **P4′** solution (*I* = 0.1 M) resulted in a more pronounced pH decrease in the initial part of the curve compared to the addition of an equivalent amount of Na^+^ to a similar solution (Figure S19A,B).

**Scheme 1:**
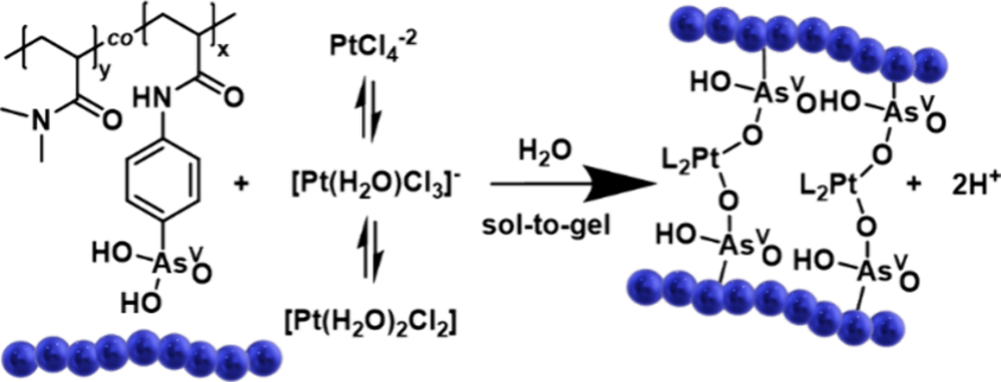
Proposed Mechanism for Gel Formation^a^

a Reactions between the arsenic acid side chains and Pt^II^ result in the release of H^+^ into solution, leading to a decrease in pH and ultimately cross-linking through coordination between the O atoms in the arsenic acid groups and Pt^II^.

**Figure 4:**
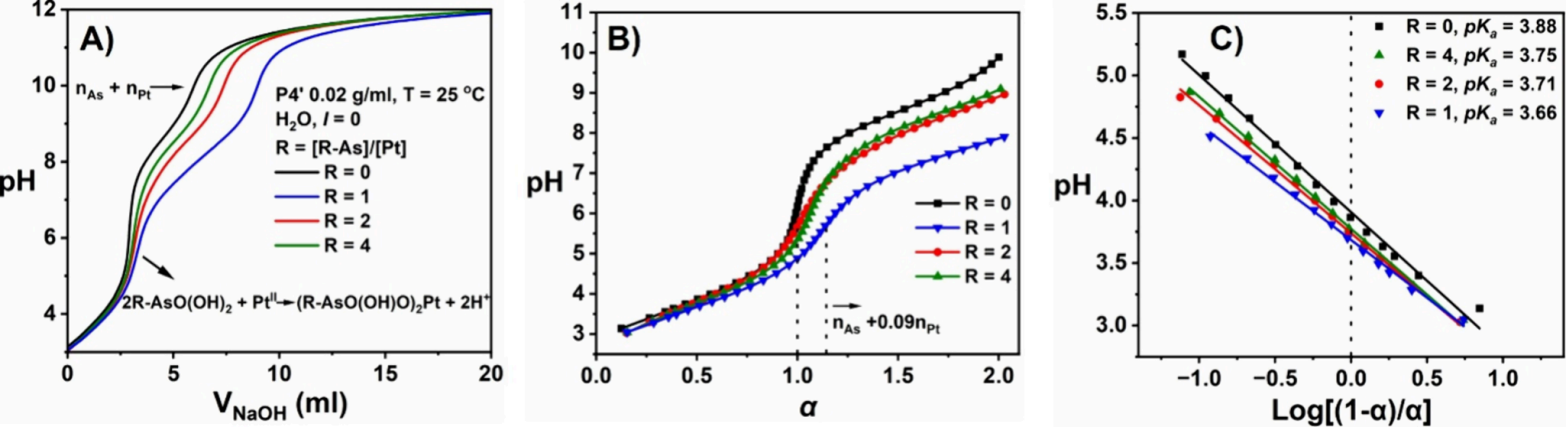
(A) Potentiometric titration curves of **P4′** in the presence of varying amounts of K_2_PtCl_4_ ([As]/[Pt] = 1–4) in H_2_O. (B) Plot of pH vs α. Closer inspection of the first part (α = 0.15–0.5) indicates that at a given α pH, the pH decreases in the presence of Pt^II^ in accordance with the mechanism described in Scheme 1. (C) Using eq 2 corresponding to p*K*_a1_, values confirm the influence of Pt^II^ on the acidity of the arsenic acid groups with p*K*_a1_ decreasing as [Pt^II^] increases.

Investigation of the first equivalence point revealed a positive shift of the end point corresponding to *n*_NaOH_ = *n*_As_ + 0.09*n*_Pt_ in the higher [Pt], i.e., [As]/[Pt] = 1. This shift suggests that protons other than those of the arsenic acid groups are titrated.

Examination of the full titration curves did not reveal a new equivalence point upon Pt^II^ addition, ruling out the involvement of amide groups in coordination. Metalation of N–H is unlikely to happen due to the very weak acidity of the amide nitrogen.^75^ Thus, the positive shift likely arises from further deprotonation of arsenic acid groups (second oxygen group) or the deprotonation of hydrated Pt^II^ species, involving the dissociation of 9% of the protons per Pt^II^ ion. For the second equivalence point observed during titration of **P4′**, the pH showed a minimal decrease at end point upon Pt^II^ addition (ΔpH_α=2_ = 0.1 at [As]/[Pt] = 1), while the additional volume of base needed for neutralization was directly proportional to [Pt] added (Figure S19).

To explore this phenomenon, titration of K_2_PtCl_4_ (6 mM) was performed in the absence of **P4′**, revealing an equivalence point at pH = 9.41 (p*K*_a_ = 7.33, Figure S21). This was associated with the deprotonation of water molecules, which are known to act as ligands to Pt^2+^ in aqueous solutions, resulting in the formation and precipitation of Pt­(OH)_2_ at pH > 9.5 (Figure S22).^65^ At pH < 9.5, this becomes less prevalent. Meanwhile, an increase in UV–vis absorbance at pH > 4 and a decrease in solution pH from 5.98 (*t* = 0) to 3.84 (*t* = 24h) at 50 °C were observed, no precipitation occurred (Figure S23A,B).

It is therefore speculated that the positive shift of the first equivalent point is attributed to hydrated Pt^II^ deprotonation, commencing at pH ≥ 4, which contributes to the greater pH decrease above α = 0.5 (Figure 4B and Figure S18). Although coordination of platinum with the second oxygen of the arsenic acid pendant group at pH 7 cannot be excluded, it is certain that the percentage of deprotonated Pt–water complexes remains below 10%, as indicated by the positive shift of *V*_eq,1_ (0.09*n*_Pt_), and is therefore deemed to be minimal in our hydrogel formulation.

A stability constant for the interaction between Pt^II^ and the arsenic acid groups was estimated by the general method reported by Bjerrum^76^ and modified by Gregor et al.^77^ and then Mandel and Leyte (see the Supporting Information for details).^78^ It is important to note that the model assumes that the interaction is between the metal ion and individual acid group and thus is independent of molecular weight and always decreases the negative charge of the polymer. Furthermore, consistent with Mandel and Leyte’s method, it is assumed that the complexation to an acid group does not influence the dissociation constant of other acid groups present. Finally, activity corrections are omitted due to the use of dilute solutions.

Initially, a reference plot for **P4**′ at *I* = 0 and *I* = 0.1 M was constructed (Figure S24). The linearity of the plot confirmed the absence of any conformational transitions of the polymer across the whole range of α for the first equivalent point. From these plots, formation curves were constructed at varying [As]/[Pt] (Figure S25). When [As]/[Pt] ≤ 2, the average number (*ñ*) of arsenic acid groups bound to Pt^II^ was calculated to be *ñ* = 1. The average increased and approached *ñ* = 2 when [As]/[Pt] = 4, i.e., when arsenic acid groups are in excess. Thus, it is proposed that under the conditions for hydrogel formation Pt^II^ can interact with arsenic acid groups from different polymer chains, resulting in efficient cross-linking. The resulting complexation reaction without taking into consideration the Pt^II^ coordination sphere to ligands other than the polymer is given below:

$$2R-\mathrm{AsO}\left(\mathrm{OH}\right)O^{-}+{\mathrm{Pt}}^{2+}\to {\left(R-\mathrm{AsO}\left(\mathrm{OH}\right)O\right)}_{2}\mathrm{Pt}\;K_{f2}$$

Finally, Mandel and Leyte’s method allowed the average complexation constant between the arsenic acid group and Pt^II^ (at *ñ* = 2) to be measured as log*B*_av_ = −1.36 ([As]/[Pt] = 4) and the overall formation constant was calculated as ***K*_f2_= 1.1 × 10^5^Μ^–2^** (log*K*_f2_ = 5.04). The stability constant is greater than those reported for electrostatic interactions.^79^ The interactions are somewhat weaker compared to previously metal-coordinated gels;^21^ however, it is noted that this promotes the tunable and self-healing mechanical properties of these hydrogels (*vide infra*). Additionally, the overall formation constant was measured under conditions of added NaClO_4_ (*I* = 0.1 M), yielding a value of ***K*_f2 (I=0.1)_= 2.3 × 10^5^M^–2^** (log*B*_av_ = −1.18). The small increase compared to the value obtained in pure water is attributed to the electrostatic influence of Na^+^ ions on the arsenic acid pendants. However, this increase is minimal, confirming the negligible effect of the inert electrolyte on the Pt^II^ complexation.

Further evidence for the proposed interactions between Pt^II^ and the arsenic acid side chain was gathered from X-ray photoemission spectroscopy (XPS) and was further corroborated by energy-dispersive X-ray spectroscopy (EDX) during scanning electron microscopy (SEM). The XPS spectrum of As 3d peaks for **P4** was deconvoluted into two peaks assigned to 3d_3/2_ and 3d_5/2_ electrons of As^V^ in the pendent arsenic acid group (*E* = 45.20 eV, Figure S26). When mixed with Pt^II^, an increase in 3d peaks of **P4-Pt** was observed, while the binding energies indicated that As remains at As^V^ (*E* = 45.40 eV, Figure S27). The deconvoluted binding energies of the Pt 4f XPS spectrum for **P4-Pt** (Figure S28) confirmed the presence of Pt^II^ after gelation with no evidence of formation of Pt^IV^ or Pt^0^. The binding energy of Pt^II^ in **P4-Pt** was found to be ∼0.8 eV higher compared to **P5-Pt** (PDMAm only, Figure S29), supporting the proposed Pt–polymer interaction upon cross-linking. Furthermore, the coordinated Pt^II^ exhibited binding energies attributable to multiple Pt–O_*x*_ rather than single Pt–O bonding. To further examine the Pt–O complexation, the O 1s spectrum was analyzed. While deconvolution was complex, it provided good evidence that the proposed Pt–O interaction was not arising between C=O of pendant amide functional groups (Figure S30A). The existence of a new peak at *E* = 529.50 eV in **P4-Pt** was assigned to the formation of As–O–Pt bonds upon cross-linking (Figure S30B). Moreover, analysis of the N 1s spectrum of **P4** and **P5** before and after addition of Pt^II^ provided additional evidence for the nonparticipation of the amide functional group in the Pt^II^–polymer interactions (Figure S31). Additionally, the atomic concentrations (%) of all important elements in **P4-Pt** was measured (Table S2). The theoretical atomic concentration (%) of arsenic (As) atoms per polymer chain (at 8 mol % AsAm) was calculated as ∼0.57%, in close agreement with the measured values of 0.66–0.69% (relative atoms/nm^2^). Likewise, [Pt] was detected at less than half concentration of [As], suggesting its preference for the interaction with two or more arsenic acid groups, which was consistent with potentiometric analysis and favorable for cross-linking. The presence of metal/metalloid components lends itself to imaging by electron microscopy. For example, polymeric arsenicals have been shown to enable near atomic resolution imaging of non-conjugated polyacrylamide scaffolds.^90^ Here, the presence of As and Pt on the surface of the hydrogels was verified through EDX during SEM imaging, with the characteristic peaks attributed to As and Pt being present in all gels (Figure 5B and Figure S32A–D). EDX mapping was used to get an insight on the distribution of As and Pt on the surface of the samples, showing that As and Pt were present all over the hydrogel’s surface. The surface and pores of freeze-dried **P4-Pt** were imaged through SEM (Figure 5A and Figure S32E).

**Figure 5:**
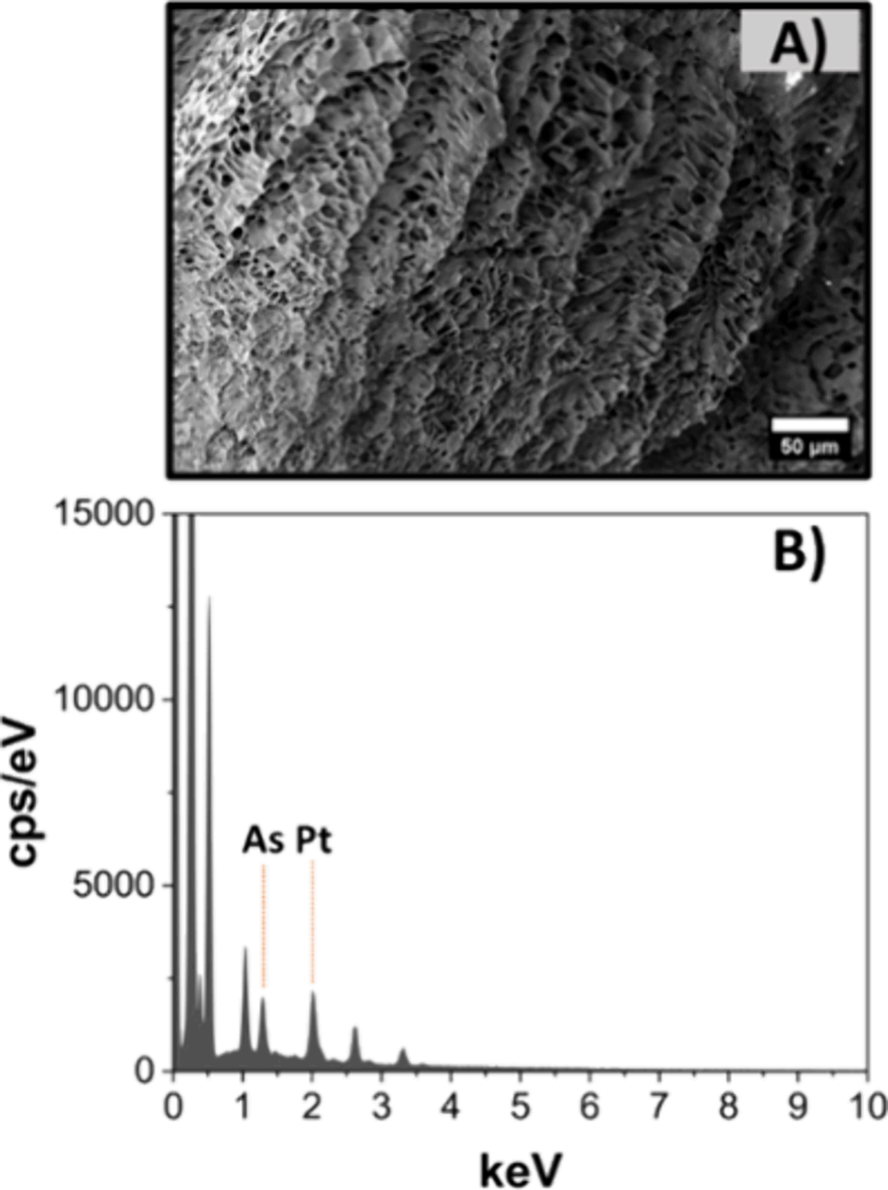
(A) Scanning electron microscopy (SEM) of dry 10 wt % **P4-Pt** after lyophilization (scale bar = 50 μm). (B) EDX spectrum of **P4-Pt** after lyophilization.

### Mechanical Properties of As^V^–Pt^II^ Hydrogels

The viscoelastic properties of **P1-Pt**–**P4-Pt** hydrogels were further investigated by plate-to-plate oscillatory rheology. Amplitude sweeps were initially obtained at 25 °C at a constant frequency of ω = 10 rad.s^–1^ to find the linear viscoelastic regions (LVE). All gels were able to withstand a large amount of deformation (γ > 900%) without losing their integrity based on the relative values of the storage (*G′*) and loss (*G*″) moduli (Figure 6A). Above γ = 900% strain, extensive slippage was observed. The *G′* values showed the tendency to lose their linearity beyond certain strain (%) leading to a yield point (τ_y_). This behavior is attributed to the gradual formation of microcracks by increasing deformation, which did not cause material failure. The yield point was found to increase with increasing AsAm mole fraction (**P1-Pt** < **P2-Pt** < **P3-Pt** < **P4-Pt**), which was attributed to a gradual increase of cross-linking density.^80^ Conversely, the *G″* values decreased with increasing AsAm, reflecting the reduction of network’s dynamic viscosity (*G*″/ω) with greater cross-linking density.^23^

**Figure 6:**
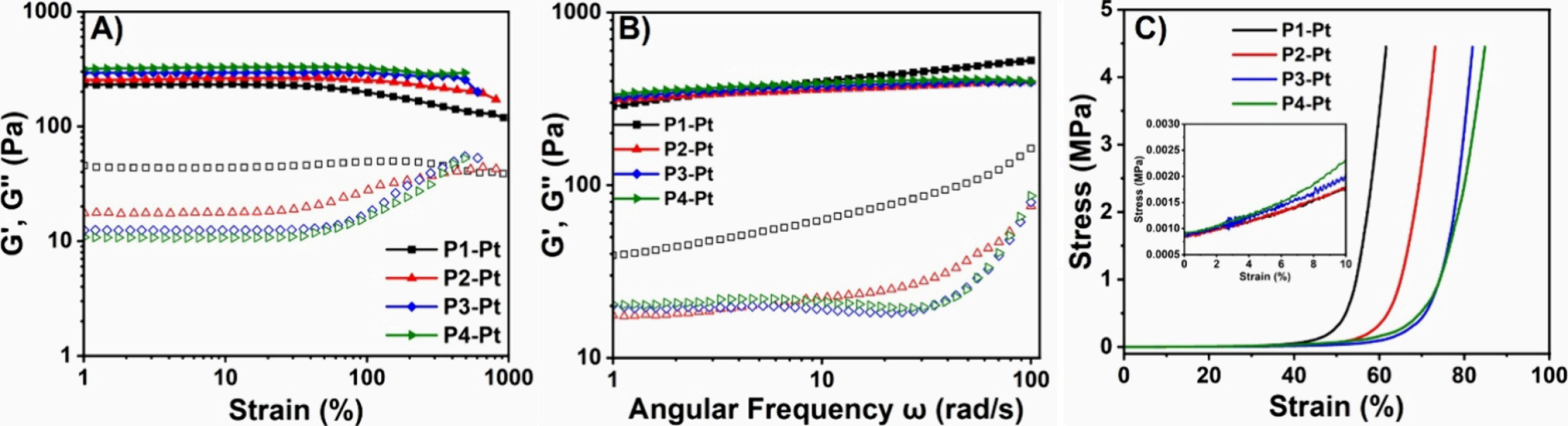
(A) Determination of the linear viscoelastic region of **P1-Pt**–**P4-Pt** from plate-to-plate oscillatory rheology. *G′* (filled in squares) and *G″* (empty squares). (B) Frequency sweeps (ω = 1–100 rad.s^–1^) of **P1-Pt**–**P4-Pt** at γ = 10% from plate-to-plate oscillatory rheology. (C) Determination of the Young’s modulus from stress/strain curves generated during compression testing (500 N load, 30 s) at 10% deformation.

Frequency sweep measurements were conducted at a constant strain of γ = 10% (within LVE region), at 25 °C and an angular frequency range of ω = 1–100 rad.s^–1^ to examine the frequency-dependent properties (Figure 6B). For every hydrogel studied, *G′* > *G*″ throughout the examined frequency range indicating fully cross-linked networks were retained. Small increases in the stiffness were observed with increasing AsAm mole fraction. Specifically, **P1-Pt** exhibited a *G′* = 286 Pa at ω = 1 rad.s^–1^, while **P2-Pt**, **P3-Pt**, and **P4-Pt**, *G′* = 310, 320, and 333 Pa, respectively (Table S3). At higher frequencies (ω = 100 rad.s^–1^), a moderate increase in the *G′* values was observed. The relatively low *G′* values obtained reflect the stability constant calculated from potentiometric titration. Furthermore, the moderate changes in *G*′ and *G*″ with applied frequency suggest that while hydrogel formation is driven by interactions between Pt^II^ and the arsenic acid groups present, these are supported by the existence of other physical interactions (e.g., chain entanglements, H-bonding, and π–π) within the network, which enforced the elastic part of the gels.

Metal–ligand coordinative networks offer a versatile platform that can alter energy dissipation and the mechanical properties of the material such as hardness, strength, and stiffness.^20,81^ To explore these effects on the properties of **P1-Pt**–**P4-Pt**, compression testing was performed. Stress/strain curves were generated (500 N load, 30 s), and the elastic Young’s modulus was measured at 10% deformation (Figure 6C). All hydrogels reached the upper maximum load of the load cell at 4.5 MPa, providing evidence of a strong material.^82^ However, without a break point, it was difficult to determine the ultimate compressive strength of each material. The Young’s modulus increase agreed with increasing AsAm mole fraction and therefore cross-linking (**P1-Pt** < **P2-Pt** < **P3-Pt** < **P4-Pt**), which agreed with the results obtained from rheology.

Consecutive compressive cycles were conducted to shed light on the energy dissipation mechanism and explore elasticity. Interestingly, for **P2-Pt**–**P4-Pt**, the stiffness increased after unloading and reloading on the second cycle, followed by a moderate increase on the third cycle (Figure S33 and Table 2). The mechanical properties of metal-coordinated networks can be influenced by the kinetic remodeling and ligand exchange at the metal center. In the case **P2-Pt**–**P4-Pt**, with increasing AsAm mole fraction, it is hypothesized that when stress is applied, reorganization of the polymer network results in the change in the coordination sphere of Pt^II^ ultimately leading to an increase in cross-linking density resulting in the hydrogel becoming stiffer over the time scale of the experiment. On the other hand, **P1-Pt** did not exhibit an increase in stiffness upon cycling (Figure S34 and Table 2). This indicates that at lower AsAm mole fraction (2 mol %), the density of available AsAm groups to interact with the coordination sphere of Pt^II^ is insufficient to promote further cross-linking. The gel shows reversible deformation upon stress and returned to its original shape after unloading instantly, providing excellent elasticity.

**Table 2:** Young’s Modulus Values (*Y*) at 10% Deformation for **P1-Pt**–**P4-Pt** Hydrogels Obtained During Three Consecutive Compression Tests

**Hydrogel**	*Y* **/kPa** **1st cycle**	*Y* **/kPa** **2nd cycle**	*Y* **/kPa** **3rd cycle**
P1-Pt	9.1	9.1	9.1
P2-Pt	9.6	18.0	16.2
P3-Pt	11.1	15.3	16.9
P4-Pt	13.3	23.4	24.2

To further understand the effect of cross-linking density on network integrity and stability, the swelling behavior of **P1-Pt**–**P4-Pt** was investigated in DI water. Weak metal–polymer coordination generally allows deep penetration of water molecules into loosely cross-linked networks.^83^

In the case of **P1-Pt** and **P2-Pt**, swelling ratios >1000% were obtained (Figure 7 and Table S4). However, at lower AsAm mole fraction (2 and 4 mol %, respectively), and therefore lower cross-linking density, destabilization of the hydrogel network was observed so experiments were stopped after 96 h.

**Figure 7:**
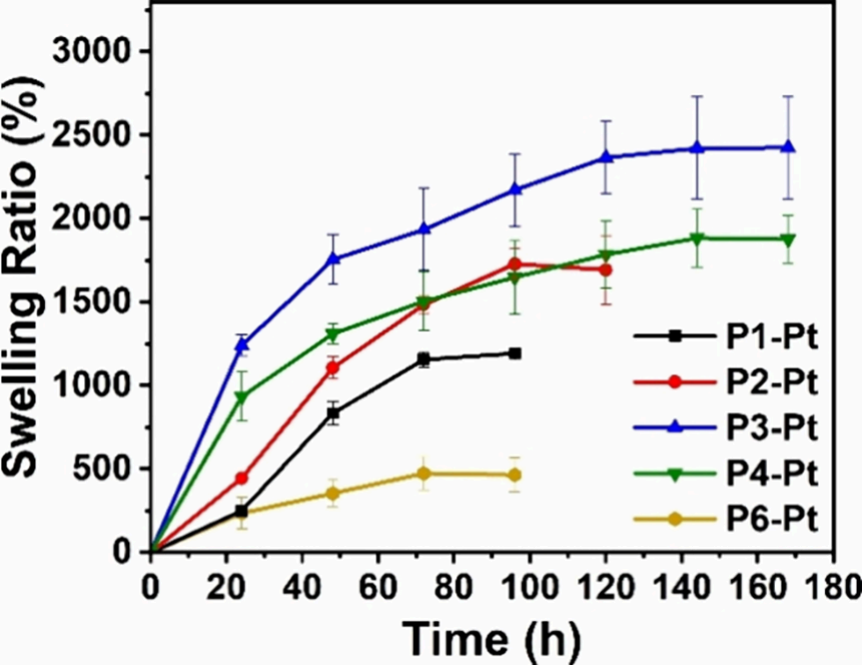
Swelling behavior of **P1-Pt**–**P4-Pt** and **P6-Pt** as a function of time investigated in DI water. At each time point, the hydrogel sample was removed from the aqueous solution, patted dry, weighed, and then returned to a fresh aqueous solution.

The nature of metal–ligand-coordinated networks depends on the hard–soft acid–base (HSAB) theory. Here, oxygen atoms of the arsenic acid moieties of the polymer behave as electron donors to soft Pt^II^ ions resulting in relatively weak As–O–Pt interactions that are likely to undergo exchange with H_2_O within the coordination sphere upon swelling. As swelling progresses, the concentration of AsAm and Pt^II^ in the swollen networks of **P1-Pt** and **P2-Pt** was insufficient to drive reformation of the As–O–Pt interactions. Consequently, **P1-Pt** was observed to undergo fragmentation after a few hours of swelling and complete dissolution was observed within 120 h (Figure S35). While dissolution of **P2-Pt** was not observed over the time of these experiments, fragmentation was again apparent (Figure S36). Conversely, **P3-Pt** and **P4-Pt** retained their structural integrity reaching equilibrium swelling after 144 h (Figure S37). **P3-Pt** (6 mol % AsAm) exhibited the higher (2425%) swelling ratio than **P4-Pt** (8 mol % AsAm, 1875%). Overall, these observations suggest that >5 mol % AsAm is required to allow efficient water penetration and swelling without losing the overall gel integrity as observed for **P1-Pt** and **P2-Pt**. The increased swelling ratio of **P3-Pt** compared to that of **P4-Pt** reflects the mechanical properties of the hydrogels. **P4-Pt** has the highest cross-linking density resulting in slightly stiffer gels with a slightly lower swelling ratio compared to **P3-Pt**.

To investigate the HSAB explanation and significance of the As–O–Pt interaction on cross-linking, a carboxylic acid equivalent of AsAm, 4-acrylamidobenzoic acid, was synthesized (Figure S38A,B). Copolymerization with dimethylacrylamide yielded **P6**, which was found to contain a comparable mole fraction of carboxylic acid pendant groups (3 mol %, Figure S39A,B) and molecular weight distribution (*M*_w_ > 300,000 g·mol^–1^, *Đ* > 2, Figure S40) compared to **P1** and **P2**. Potentiometric titration of **P6** revealed that pendant carboxylic acid groups (p*K*_a_ = 4.55, Figure S41) were less acidic than the pendant arsenic acid groups (p*K*_a1_ = 3.88, Figure 3), suggesting that the arsenate conjugate base is better stabilized and softer, by comparison, than the carboxylate. This was manifest in hydrogel **P6-Pt** wherein weaker interactions with Pt^II^ led to diminished swelling ratios (<500%) before loss of gel integrity (Figure 7, Figure S42, and Table S3). Furthermore, attempts to form hydrogels of **P4** with harder metal ions, such as Ca^II^ (CaCl_2_) were unsuccessful, while borderline hard/soft Cu^II^ (from CuCl_2_ and CuSO_4_) formed a hydrogel with **P4** within 30 min at 25 °C (Figure S43). Finally, the presence of high concentrations of hard monovalent metal ions (Na^+^) did not perturb gel formation with Pt^II^ (Figure S44).

Considering that metal-coordinated cross-linked networks have emerged as a promising platform for self-healing biomaterials, the self-healing properties of **P1-Pt**–**P4-Pt** were investigated. Freshly prepared hydrogels were cut in half before being placed in a humidity chamber with both halves in contact along the original point of incision. After 1 h, oscillatory strain rheology (ω = 10 rad.s^–1^) revealed that *G*′ > *G*″ across the applied strain, with comparable *G*′, *G*″, and τ_y_ values to the original gels (Figure 8 and Figure S45). This suggests that **P1-Pt**–**P4-Pt** were able to self-heal into free-standing hydrogels (Figure S46).^84^

**Figure 8:**
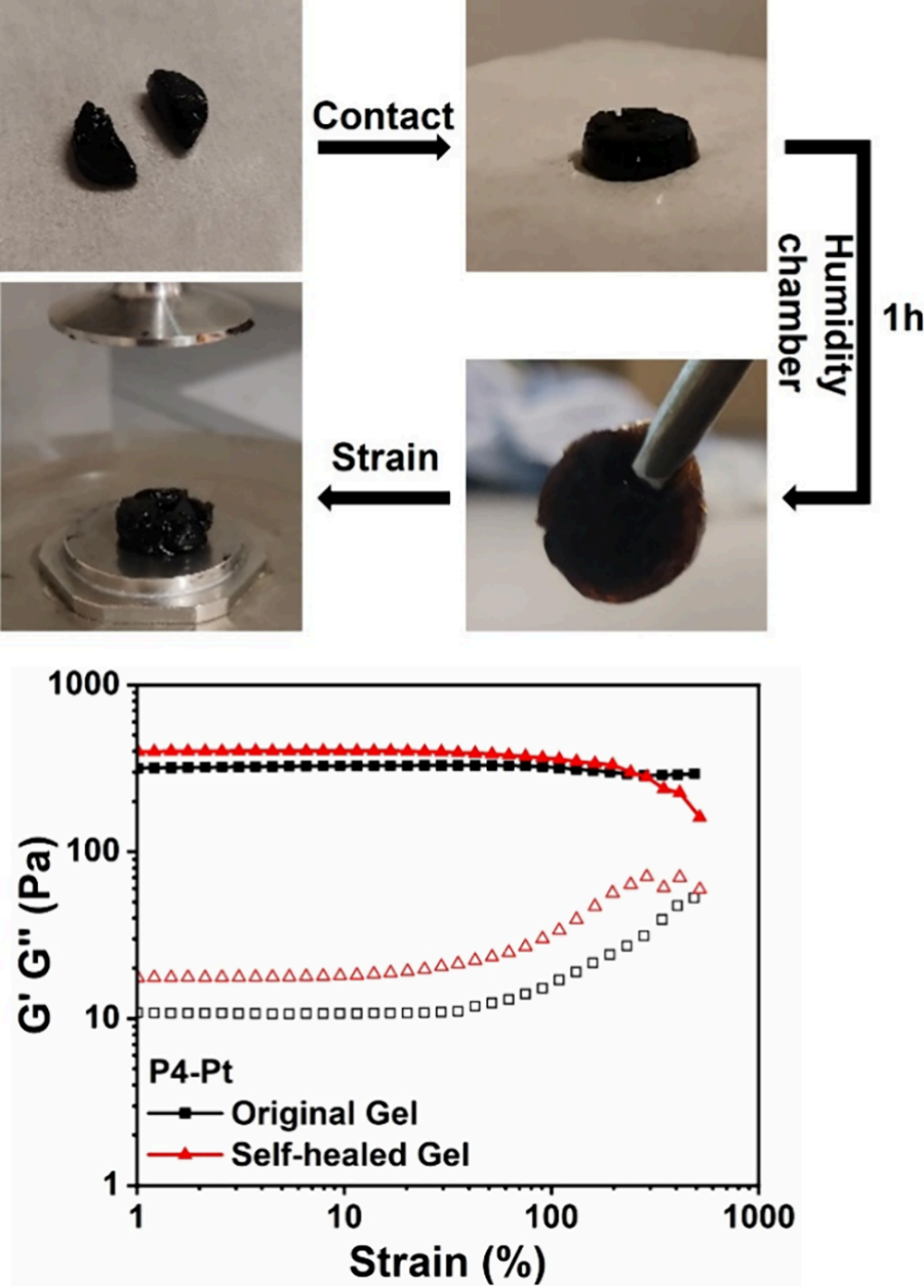
Representative demonstration of the self-healing potential of **P1-Pt**–**P4-Pt**. Freshly prepared **P4-Pt** was cut in half before being placed in a humidity chamber with both halves in contact along the original incision. Oscillatory strain rheology (ω = 10 rad.s^–1^) was performed at 1 h revealing *G*′, *G*″, and τ_y_ values comparable to the freshly prepared gel. Video’s available in the Supporting Information files.

Qualitative stretching of the healed gels was performed, and their resilience was recorded. Recovered **P1-Pt** and **P2-Pt** showed good resilience to stretching (Videos SV1 and SV2), while **P3-Pt** and **P4-Pt** with greater cross-linking density demonstrated poor resilience to stretching, readily breaking at the original point of incision (Videos SV3 and SV4). This can be related back to the mechanical properties of the original hydrogels, with the stiffer gels **P3-Pt** and **P4-Pt** exhibiting limited chain mobility due to stronger coordination, which hinders the reorganization of the Pt^II^ coordination sphere along the original point of incision. Furthermore, this restricts the ability to exploit supporting physical interactions that give rise to the elastic properties observed for **P1-Pt**. Although stronger coordinating agents enhance shape stability and increase cross-linking density, they also lead to higher relaxation times and slower or poorer self-recovery.^32,85^

Overall, the results from rheology, compression testing, swelling, and self-healing are in line with other reported metal-coordinating systems that can undergo changes in their coordination sphere enabling tuning of the mechanical properties at different [M]:[L] stoichiometries. Here, the combination of previously reported polymeric arsenical scaffolds with Pt^II^ forms a cross-linked network generating soft, strong, and self-healing hydrogels with tunable stiffness and elasticity.

### Antimicrobial Properties of As^V^–Pt^II^ Hydrogels

Preliminary, qualitative evaluation of the potential antimicrobial activity of As^V^–Pt^II^ hydrogel **P4-Pt** was performed via antibiotic disk diffusion assays against Gram-negative (UPEC, E. coli K12MG1655) and Gram-positive (B. subtilis and S. aureus) bacterial strains. Initially, K_2_PtCl_4_ was run as a positive Pt control showing a zone of inhibition against each bacterial strain, with notably greater inhibition against *UPEC* and E. coli K12MG1655. A polymer scaffold (**P4**) was used as an As control, showing only faint zones of inhibition, while in the absence of Pt^II^ and As^V^, there was no observable zone of inhibition (Figure S47). Pleasingly, when **P4-Pt** hydrogel was employed in the disk diffusion assay, a zone of inhibition comparable to the Pt control was observed with more prominent inhibition observed against *UPEC* and E. coli K12MG1655 (Figure S48) compared to B. subtilis and S. aureus. Cytotoxicity has been reported for Pt^II^ salts due to their interaction with DNA^86,87^ or binding to protein molecules,^88^ which induces cell death. However, the available data are limited compared to those for other Pt^II^-based cytostatic drugs, such as cisplatin.^89^ In contrast, **P4** is nontoxic, as demonstrated here and in our previous work.^52^ While the mode of action is currently speculative, these preliminary results confirm that As^V^–Pt^II^ hydrogels have some antimicrobial activity, and further research is required to fully quantify and benchmark the efficacy of the system and understand the mechanisms of action.

## Conclusions

Polymeric arsenical scaffolds (**P1**–**P4**) have been mixed with Pt^II^ (from K_2_PtCl_4_) giving rise to hydrogels (**P1-Pt**–**P4-Pt**). From spectroscopic techniques and potentiometric titration, it has been proposed that **P1-Pt**–**P4-Pt** formation is driven by interactions between Pt^II^ and oxygen atoms present in the pendant arsenic acid (AsO_3_H_2_) groups of the polymer scaffolds. Evaluation of hydrogel formation kinetics demonstrated that the rate of cross-linking increased with increasing AsAm mole fraction (**P4** > **P3** > **P2** > **P1**). Rheology, compression testing, and swelling tests revealed hydrogels that were soft but strong with tunable properties that are dependent on the [As]/[Pt] ratio and therefore cross-linking density. The hydrogels also demonstrated potential for self-healing, with free-standing hydrogels yielding comparable moduli (*G*′, *G*″) and yield points (τ_y_) by rheology obtained after **P1-Pt**–**P4-Pt** were cut in half and healed in a homemade humidity chamber. Finally, preliminary and qualitative antimicrobial evaluation indicated that **P4-Pt** had comparable activity to Pt^II^ against Gram-negative (*UPEC*, E. coli K12MG1655) and Gram-positive (B. subtilis and S. aureus) bacterial strains. Further development and evaluation of new therapies involving Pt and As delivery are required to help address some of the limitations associated with existing Pt (resistance) As (acute toxicity)-based therapeutics, which is subject to ongoing work in our research program.

## Supplementary Material











## Notes

Raw data sets available from the authors upon request. The Supporting Information is available free of charge at https://pubs.acs.org/doi/10.1021/acs.chemmater.5c00184.

Additional experimental details, supporting images and tables, NMR, FT-IR, UV-Vis, XPS, SEM/EDX, SEC, potentiometric titration and pH changes, water ligand exchange, reference plots, antibiotic diffusion assays, macroscopic demonstration of self-healing, rheological properties and swelling ratios (PDF)
Video recording, demonstrating the mechanical resilience of the self-healed **P1-Pt** hydrogel upon stretching (SV1) (MP4)
Video recording, demonstrating the mechanical resilience of the self-healed **P2-Pt** hydrogel upon stretching (SV2) (MP4
Video recording, demonstrating the mechanical resilience of the self-healed **P3-Pt** hydrogel upon stretching (SV3) (MP4)
Video recording, demonstrating the mechanical resilience of the self-healed **P4-Pt** hydrogel upon stretching (SV4) (MP4)

## †

Medherant Ltd., The Venture Centre, University of Warwick Science Park, Coventry, CV4 7EZ, U.K. (S.E.)

## Abbreviations

AsAm: 4-(*N*-acrylamido)­phenylarsonic acid
DMAm: *N*,*N*-dimethylacrylamide
NMR: nuclear magnetic resonance
FT-IR: Fourier-transform infrared spectroscopy
SEM: scanning electron microscopy
XPS: X-ray photoelectron spectroscopy
UPEC: uropathogenic *Escherichia coli*
SEC: size exclusion chromatography
FRP: free radical polymerization
VA-044: 2,2′-azobis­[2-(2-imidazolin-2-yl)­propane] dihydrochloride
LVE: linear viscoelastic region
HSAB: hard-soft acid–base theory
EDX: energy-dispersive X-ray spectroscopy
UV–vis spectroscopy: ultraviolet–visible spectroscopy
