# Supplementary material for: A Tunable Hydrogel Platform Based on Platinum-Containing Polymeric Arsenicals
Source: Chem Mater. 2025 May 7;37(10):3733–46. doi: 10.1021/acs.chemmater.5c00184 (PMC12120916; doi:10.1021/acs.chemmater.5c00184)
Supplement: Supplementary file 1 [file cm5c00184_si_001.pdf]

Supplementary Information

A tuneable hydrogel platform based on platinum-containing polymeric arsenicals

Alexandros Magiakos,[a] Evelina Liarou,[a] Spyridon Efstathiou,[a] Andrea Dsouza,[a,b] Chrystala Constantinidou,[b] Marc Walker,[c] Constantinos Methenitis,[d] and Paul Wilson*[a]

[a]Department of Chemistry, University of Warwick, Coventry, CV4 7AL, UK

[b]Warwick Medical School, University of Warwick, Coventry, CV4 7AL, UK

[c]Department of Physics, University of Warwick, Coventry, CV4 7AL, UK

[d]Department of Chemistry, National and Kapodistrian University of Athens, Panepistimioupolis, GR-15771, Greece

*Email: p.wilson.1@warwick.ac.uk

TABLE OF CONTENTS

Supplementary experimental details S4

Preparation of protonated polymer scaffolds S4

Preparation of phosphate buffer solutions S4

General considerations for potentiometric titration analysis and K_f determination.................. S4

Figure S1A. ^{1}H NMR spectrum (400 MHz, D_2O/NaOH 1 M) of 4-(*N*-acrylamido)phenylarsonic acid (AsAm).. S7

Figure S1B. ^{13}C NMR (400 MHz, D_2O/NaOH 1 M) spectrum of 4-(*N*-acrylamido)phenylarsonic acid (AsAm).. S8

Figure S1C. FT-IR spectrum of 4-(*N*-acrylamido)phenylarsonic acid (AsAm) in the range of 500 – 4000 cm^{-1} (left) and 1500 – 1700 cm^{-1} (right).. S8

Figure S2A. ^{1}H NMR spectra (400 MHz, D_2O) of P1 – P4 polymerics arsenical scaffolds S8

Figure S2B. FT-IR spectra of P1 – P4 in the range of 500 – 4000 cm^{-1} (left) and 500 – 1100 cm^{-1} (right) S9

Figure S3. Aqueous SEC of P1 – P4 polymeric arsenicals S9

Figure S4A. ^{1}H NMR spectrum (400 MHz, D_2O) of PDMAm / P5 (left) and FT-IR spectrum in the range of 600 – 4000 cm^{-1} (right)... S10

Figure S4B. DMF SEC of PDMAm (P5) S10

Figure S5. Image of 10 wt% P1-Pt – P4-Pt hydrogels fabricated at 50 °C S11

Figure S6. UV-Vis spectrum of P4 polymer scaffold in H_2O, recorded in the range of 200 – 500 nm.. S11

Figure S7. Blue shift of K_2PtCl_4 at λ = 391 nm after 48 h at 25 °C, attributed to ligand exchange (Cl^- → H_2O) in the cis position S12

Figure S8B. Images of a 30 mM K_2PtCl_4 solution at t =0 and t = 48h, incubated at 50 °C S13

Figure S9A. Gelation time (t_{lag}) at 50 °C for polymer scaffolds with varying AsAm content.... S13

Figure S9B. Gel completion time for different AsAm content hydrogels (P1-Pt – P4-Pt)........ S13

Figure S10. ^{1}H NMR spectra (400 MHz, D_2O) of P5 and P5-Pt showing no shifts in the backbone proton signals upon mixing with K_2PtCl_4 .... S14

Figure S11. FT-IR of P4 and P4-Pt showing no change to the amide region upon mixing with K_2PtCl_4 .... S14

Figure S12. ^{195}Pt NMR spectrum (600 MHz, D_2O) of the P4-Pt hydrogel in the range of -3400 to -3700 ppm S15

Figure S13. ^{195}Pt NMR spectrum (600 MHz, D_2O) of the P4-Pt hydrogel in the range of -1000 to -1650 ppm S15

Figure S14. ^{195}Pt NMR spectrum (600 MHz, D_2O) of a 30 mM K_2PtCl_4 solution in D_2O, recorded in the range of -1000 to -1650 ppm S16

Figure S15A. Downfield shift of aromatic protons in the ^{1}H NMR spectrum (400 MHz, D_2O) of the P4' polymer scaffold, attributed to the complete protonation of arsenic acid groups (AsAm pendants) S16

Figure S15B. FT-IR spectrum of fully protonated P4' polymer scaffold S17

Figure S15C. Aqueous SEC of P4' low molecular weight polymeric arsenical scaffold S17

Figure S16. Potentiometric titration of AsAm monomer in H_2O (MeOH 64%) with 0.1 M $NaClO_4$. Henderson-Hasselbalch plots, using Equation 3, to determine pK_a and n values (right) S18

Figure S17. Potentiometric titration of P5 in H_2O at 25 °C, showing the absence of equivalent point S18

Figure S18. Potentiometric titrations of P4' in H_2O and 0.1 M $NaClO_4$ in the absence and presence of K_2PtCl_4 with [As]/[Pt] ratios of 1 – 4 (left). A drop in solution pH is observed with the progress of deprotonation-complexation upon addition of Pt^{II} ([As]/[Pt] = 1 – 4) at the first part of the pH = f(α) curve (right) S19

Figure S19A. 10 wt% solution of P4 in the presence of 0.1 M $NaClO_4$ (right) and 0.1 M NaCl (left) after overnight incubation at 50 °C S20

Figure S19B. pH change of a P4' solution with I = 0.1 M upon the addition of equivalent amounts of Pt^{II} and Na^+ S20

Figure S20. Positive shift of the second equivalence point ($V_{eq,2}$) of P4' directly proportional to the concentration of Pt^{II} added S21

Figure S21. Potentiometric titration of 6 mM K_2PtCl_4 solution in presence and absence of electrolyte ($NaClO_4$ 0.1 M) S21

Figure S22. Water ligand exchange in aqueous solution of K_2PtCl_4 (30 mM) and precipitation of the dark $Pt(OH)_2$ after deprotonation at pH > 9.5 .... S22

Figure S23A. Image of K_2PtCl_4 solutions (7.5 mM) in different pH media incubated at 50 °C for 24 hours. Solutions: pH = 2, pH = 4, PBS (1X, pH = 7.4) and H_2O (left to right) S22

Figure S23B. UV-Vis spectra of K_2PtCl_4 solutions (7.5 mM) in different pH media, recorded at t = 0 (left) and after 24 hours (right) of incubation at 50 °C S22

Figure S24. Reference plot p([H^+]/HA]) vs p[A^-] for P4' (0.02 g/ml) in DI water with no added electrolyte (left). At I = 0.1 M (right) S23

Figure S25. Typical formation curve $\tilde{n}$ vs p([HA]/H^+]) for P4'/Pt in DI water with no added electrolyte ([As]/[Pt] = 1 – 4, left). At I = 0.1 M ([As]/[Pt] = 4, right) S23

Figure S26. As3d XPS spectrum of P4 polymer, with the peak deconvoluted into $3d_{5/2}$ and $3d_{3/2}$ components S24

Figure S27. As3d XPS spectrum of dried P4-Pt gel, with the peak deconvoluted into $3d_{5/2}$ and $3d_{3/2}$ components S24

Figure S28. Pt4f XPS spectrum of dried P4-Pt gel, with the peak deconvoluted into $4f_{7/2}$ and $3d_{5/2}$ components S25

Figure S29. Pt4f XPS spectrum of dried P5-Pt mixture (PDMAm with 30 mM of K_2PtCl_4), with the peak deconvoluted into $4f_{7/2}$ and $3d_{5/2}$ components S25

Figure S30A. Deconvoluted XPS peaks of the -C=O group for the P4 polymer and dried P4-Pt gel S26

Figure S30B. O1s XPS spectrum of dried P4-Pt gel with deconvoluted corresponding components S26

Figure S31. N1s XPS spectra of P5 and dried P5-Pt mixture (PDMAm with 30 mM K_2PtCl_4) on the left, and P4 and dried P4-Pt gel on the right S27

Figure S32A. SEM/EDX spectrum of the corresponding P1-Pt dried gel image S28

Figure S32B. SEM/EDX spectrum of the corresponding P2-Pt dried gel image. S28

Figure S32C. SEM/EDX spectrum of the corresponding P3-Pt dried gel images. S29

Figure S32D. SEM/EDX mapping analysis for the P4-Pt dried gel, showing the distribution of As and Pt on the gel's surface S29

Figure S32E. Scanning electron microscopy (SEM) images of P4-Pt dried gel, showing surface morphology and pore structure S30

Figure S33. Consecutive stress-strain curves on P2-Pt, P3-Pt and P4-Pt hydrogels (left to right) obtained from cyclic compression tests. S31

Figure S34. Consecutive stress-strain curves on P1-Pt hydrogel obtained from cyclic compression tests S31

Figure S35. Images of P1-Pt hydrogel at t = 0 and after 120 hours of swelling in DI water S32

Figure S36. Images of P2-Pt hydrogel at t = 0 and after 120 hours of swelling in DI water S32

Figure S37. Images of P3-Pt and P4-Pt hydrogel at t = 0 and after 120 hours of swelling in DI water. Comparison of the sizes of P3-Pt and P4-Pt samples at swelling equilibrium S32

Figure S38A. ^1H NMR spectrum (400 MHz, D_2O/NaOH 1 M) of 4-acrylamidobenzoic acid ... S33

Figure S38B. FT-IR spectrum of 4-acrylamidobenzoic acid in the range of 500 – 4000 cm^{-1} (left) and 1200 – 1750 cm^{-1} (right) S33

Figure S39A. ^1H NMR spectrum (400 MHz, D_2O) of P6 copolymer of DMAm and 4-acrylamidobenzoic acid S34

Figure S39B. FT-IR spectrum of P6 copolymer with DMAm and 4-acrylamidobenzoic acid in the range of 500 – 4000 cm^{-1} S34

Figure S40. Aqueous SEC of P6 copolymer with DMAm and 4-acrylamidobenzoic acid S35

Figure S41. Titration curves of P6 in H_2O (solid line) and 0.1 M $NaClO_4$ (dashed) on the left. Corresponding Henderson-Hasselbalch plots, using Equation 3, to determine pK_a and n values on the right S35

Figure S42. Images of P6-Pt hydrogel at t = 0 and after 100 hours of swelling in DI water S36

Figure S43. 10 wt% solution of P4 in the presence of 30 mM $CuSO_4$, $CuCl_2$ and 0.1 M $CaCl_2$ S36

Figure S44. 10 wt% solution of P4 in the presence of 1 M and 3 M $NaClO_4$ (left). Formation of P4-Pt gel in 1 M $NaClO_4$ aqueous media (right)..S36

Figure S45. Cycled amplitude sweeps for P1-Pt, P2-Pt, and P3-Pt at 25 °C and ω = 10 rad s^{-1} before and after 1 hour healing in a humidity chamber..S37

Figure S46. Macroscopic demonstration of self-healing properties of P1-Pt, P2-Pt, and P3-Pt hydrogels after cutting and recombination for 1 hour in a humidity chamber at 25 °C. S37

Figure S47. Antibiotic diffusion assay for As and Pt controls against *UPEC, E. coli K12MG1655, S. aureus* and *B. subtilis*...S38

Figure S48. Antibiotic diffusion assay for P4-Pt hydrogels against *UPEC, E. coli K12 MG1655, S. aureus* and *B. subtilis.* S39

References...S40

TABLES

Table S1. Decrease in pH at different neutralization points for a P4' solution in the presence and absence of inert electrolyte ($NaClO_4$) upon the addition of 30 mmol Pt^{II}................................S19

Table S2. Atomic concentrations (%) of key elements in P4-Pt determined by X-ray photoelectron spectroscopy (XPS)...S27

Table S3. Rheological properties of P1-Pt – P4-Pt gels. Storage modulus (*G'*) and loss modulus (*G''*) were determined by frequency sweep experiments at ω = 1 rad/s and ω = 100 rad/s.......S30

Table S4. Swelling ratios' of P1-Pt – P4-Pt and P6-Pt as a function of time investigated in DI water S31

Supplementary experimental Details

Preparation of protonated polymer scaffolds. Polymer samples were acidified (pH = 1) with solution of HCl 2M before dialyzed against milli-Q water (SpectrumLabs PreWet Laboratory dialysis membrane 6000 Da) for a week and lyophilized to afford a white solid. The efficient acidification was confirmed by the downfield shift of AsAm aromatic protons in ^{1}H NMR spectra (**Figure S15A**).

Preparation of phosphate buffer solutions. pH 2, 0.01 M, I = 0.12: 0.98 g of phosphoric acid (Mr = 98) were dissolved in approximately, 900 ml of milli-Q water and 6.743 g NaCl (Mr = 58.44) were added to the solution. Few drops of 37% HCl were used to adjust the pH before making up the volume to 1L. pH 4, 0.05 M, I = 0.12: 5.04 g Na_2HPO_4 (Mr = 141.96) and 3.01 g KH_2PO_4 (Mr = 136.10) were dissolved in approximately, 900 ml of milli-Q water. Few drops of HCL 0.2 M or glacial acetic acid were utilized to adjust the pH before making up the volume to 1L.

Safety All experiments involving *p*-arsanilic acid and derivatives were conducted in a fume hood with appropriate PPE, and waste was disposed of according to institutional hazardous waste protocols to ensure safety and compliance with regulations.

General considerations for potentiometric titration analysis and K_f determination

To investigate the behaviour of a polyelectrolyte in solution, the following factors need to be considered:[1]

- Repulsive electrostatic forces from the charged groups of the macromolecule. They are called 'long distance interactions' and they are responsible for the extension of the polymer chains.
- The cohesive forces, namely 'short distance interaction' that are consisted of Van der Waals interactions between hydrophobic groups of the polymer and hydrogen bonds. They are responsible for the shrinking of polymer chains leading to compact conformations.

Given the fact that a polyelectrolyte can be (de)ionized like PMMA or PAA, the conformation adaptation is strongly dependent on the degree of dissociation (α) of the ionizable group. Therefore, the different conformations vary between two different limiting conditions within the numbers of $\alpha = 0$ and $\alpha = 1$. When $\alpha = 0$ the polymer adopts a compact formulation, while in $\alpha = 1$, an extended.[2] During an acid-base titration the changes of pH upon the addition of a base in the system is recorded. In a monoprotic acid-base titration, the degree of dissociation $\boldsymbol{\alpha}$ of an acidic group (HA) can be defined by the equation,[3]

$$\boldsymbol{a} = \frac{[\boldsymbol{base}]+[\boldsymbol{H}^{+}]-[\boldsymbol{OH}^{-}]}{[\boldsymbol{HA}]}, \qquad \boldsymbol{Eq\ 1}$$

where [base], $[H^+]$, and $[OH^-]$ are the molarities of added base, free proton, and hydroxide anion, respectively, and [HA] is the total concentration of acid groups expressed in mol/L. $[H^+]$and $[OH^-]$ ions can be obtained from pH values, assuming the activity coefficient is unity ($\alpha = 1$ in complete neutralisation). This equation has large uncertainty in low dissociation and is not calculated at values $\alpha < 0.15$.[4] It has been observed that the titration curve for many polyelectrolytes over a large range of a values, conforms to the extended Henderson-Hasselbach equation,[5]

$$\boldsymbol{pH = pK_{1/2} - nlog\left[\frac{1-a}{a}\right]}, \qquad \boldsymbol{Eq\ 2}$$

where $pK_{1/2}$ and n are constants at constant polymer and neutral salt concentrations (ionic strength). These two parameters reflect the strength of the electrostatic interactions between the ionizable groups and thus are dependent on the ionic strength and on the conformation of the polymer. From the slope of Henderson-Hasselbach equation (pH=f(log[(1-α)/α]), we can measure n which gives us the deviation of polyacid's behaviour from its monoprotic acid monomers.

A complete titration curve can be given by plotting apparent dissociation constant (pK_a) against the degree of dissociation (α). Thus, we may determine pK_a from the titration data using the modified Henderson-Hasselbach equation,

$$\boldsymbol{pK_a = pH - nlog\left(\frac{1-a}{a}\right)}, \qquad \boldsymbol{Eq\ 3}$$

The large dependence of pK_a on a is generally attributed to the increase of the electrostatic free enthalpy of the ionized polymer during the titration. Thus, it depends on the polymer conformation and the electrostatic potential Y of the existing charges in polymer chain. Katchalsky et al. confirmed the following observations:[6]

- pK_a is independent of degree of polymerization (as does n) and $pK_{1/2}$ equals to the average of the apparent pK_a in a range symmetrical around $\alpha = 0.5$.
- K_a increases with ionic strength, while n constant increases with dilution of polymer solution and decreases with the increase of ionic strength.

By extrapolating the pK_a curve to zero degree of neutralization, the negative logarithm of the intrinsic dissociation constant pK_0 can be determined. pK_0 and $pK_{1/2}$ are the characterizing constants for a polyacid. In the case of a small molecule, $pK_{1/2}$ is equal to pK_0 (namely, pK_a), constant in the whole range of α and $n = 1$. A slight deviation of this model is observed in high molecular weight (<500 kDa) due to the influence of the polymer charged ions to K_0 and $K_{1/2}$, though negligible in our case where the ionizable groups are only 8 mol% in the best case.[7-9]

Following the processing of above equations, we can construct the pH = f(α), pH = f(log[(1-α)/α]) and pK_a = f(α) plots and extract valuable information about the polyelectrolyte system.

The stability constant of Pt^{II} interaction with the arsenic groups were determined by the general method of Bjerrum[10] modified first by Gregor et al.[11] and then by Mandel and Leyte.[12] For the implication of the method Mandel and Leyte assumed that the interaction is between the metal ion and the individual acid group (rather than with the polyelectrolyte as a whole), thus is independent of molecular weight and always decreases the negative charge of the polymer. Considering the length of polymer chain, it is assumed that the complexation of part of acid groups does not influence the dissociation constant of the others (except from the charge and concentration changes). Moreover, activity corrections are omitted due to diluted solutions.

The complexation involves the deprotonation of acidic group and the direct reaction of the deprotonated group with the metal ion, following the below scheme :

$$\mathbf{M^{+2} + A^- \rightarrow MA^+} \quad \boldsymbol{k_1}, \quad \textbf{\textit{Eq} 4}$$

$$\mathbf{MA^+ + A^- \rightarrow MA_2} \quad \boldsymbol{k_2}, \quad \textbf{\textit{Eq} 5}$$

$$\mathbf{MA_{(j-1)} + A^- \rightarrow MA_j} \quad \boldsymbol{k_j}, \quad \textbf{\textit{Eq} 6}$$

Where k_1, k_2, k_j the constant formations of each complexation step with j the number of different reacting groups. The reaction (6) is expressed for a metal ion of unspecified charge. Knowing that $K_a = \frac{[H^+][A^-]}{[HA]}$ (***Eq*** **7**), the overall stability constant at every step (B_j) including the deprotonation of the acid groups can be described by the equation:

$$\mathbf{HA + MA_{j-1} \rightarrow MA_j + H^+}, \quad \boldsymbol{B_j} \quad \textbf{\textit{Eq} 8}$$

where $\boldsymbol{B_j} = \frac{[\mathbf{MA}_j][\mathbf{H}^+]}{[\mathbf{MA}_{j-1}][\mathbf{HA}]}$ (**Eq 9**). The relation between all constants will be $B_j = K_a k_j$ (***Eq*** **10**) and $K_{fj} = k_1 k_2 k_{j-1}$ (***Eq*** **11**). The relation (10) describes the overall formation constant for the total of j complexation-deprotonation steps. In Bjerrum method, Gregor showed that the titration data can be transformed into a formation curve $\tilde{n}$ = f{p([HA]/[H^+]), where $\tilde{n} = \frac{\sum_{j=1}^{N} j B_j (\frac{HA}{H^+})^j}{1 + \sum_{j=1}^{N} B_j (\frac{HA}{H^+})^j}$ (***Eq*** **12**) is the average number of ligands bound to one metal ion and results from the reaction (8). The values of p([HA]/[H^+]) in the half of $\tilde{n}$ values give logB_j, while ñ indicates the number of groups reacting per metal ion. For example, if $\tilde{n}$ =1, p([HA]/[H^+]) = logB_1 at $\tilde{n}$ = 0.5. When the curve appears with low value of slope (flat regions), this corresponds to a region in which one complex dominates and constants can be calculated separately for every complexation step. In case that this separation is not clear and the complexes coexist, the p([HA]/[H^+]) for the $\tilde{n}$ value at $\tilde{n}$/2, results in an average stability constant (B_{av}) of the $\tilde{n}$ complexation-deprotonation steps, hence $B_{av} = \sqrt{B_1 B_2}$ for a two-step reaction. Following the relation (10), $B_1 = K_a k_1$ and $B_2 = K_a k_2$ for the metal-polymer binding with one or two groups respectively. Apparent $K_{1/2}$ is used as K_a. The overall formation constant for the two-coordinated complexation reaction ($K_{f2} = k_1 k_2$) can now be calculated as $\mathbf{log}\boldsymbol{K_{f2}} = \mathbf{2log}\boldsymbol{B_{av}} + \mathbf{2p}\boldsymbol{K_{1/2}}$ ($B_{av}^2 = B_1 B_2 = K_{1/2}^2 K_{f2}$) (***Eq*** **13**).

The value $\tilde{n}$ can be extracted from the relation $\boldsymbol{\tilde{n}} = \mathbf{([A_t] - [HA] - [A^-])/[M_t]}$ (***Eq*** **14**), where [A_t] and (M_t) are the total concentrations of ligand and metal ion, [A^-] and [HA] are the concentrations of free and protonated ligand in the moment t. Consequently, [HA] = [A_t](1 - α) - [H^+] (***Eq*** **15**) with α as the dissociation constant of the polyelectrolyte. While in monomer the [A^-] can be measured from the K_a at every moment, in polyelectrolyte solution it's not possible since K_a is not constant. Mandel and Leyte developed a process to measure the [A^-] by constructing a reference plot p([H^+]/HA]) = f(p[A^-]) of the polymer in same concentrations, but absence of metal. This is used as a standard curve to calculate the formation curves in presence of different concentrations of metals.

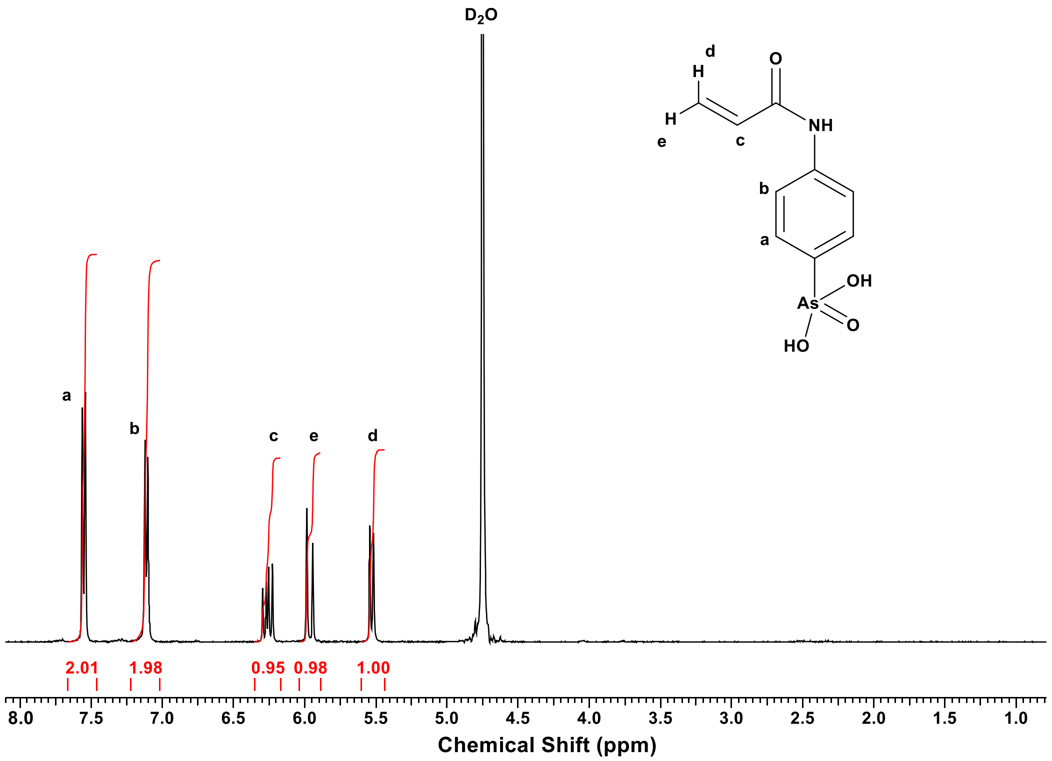

Figure S1A. ^{1}H NMR spectrum (400 MHz, D_2O/NaOH 1 M) of 4-(*N*-acrylamido)phenylarsonic acid (**AsAm**).

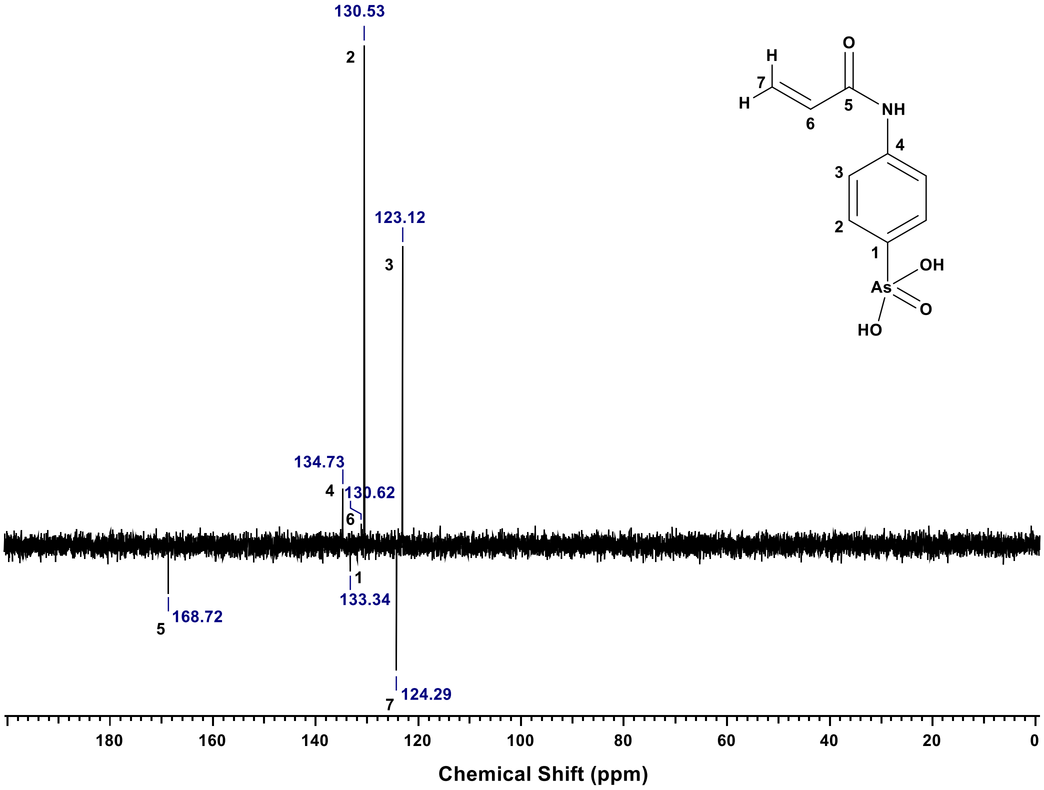

Figure S1B. ^{13}C NMR (400 MHz, D_2O/NaOH 1 M) spectrum of 4-(*N*-acrylamido)phenylarsonic acid (**AsAm**).

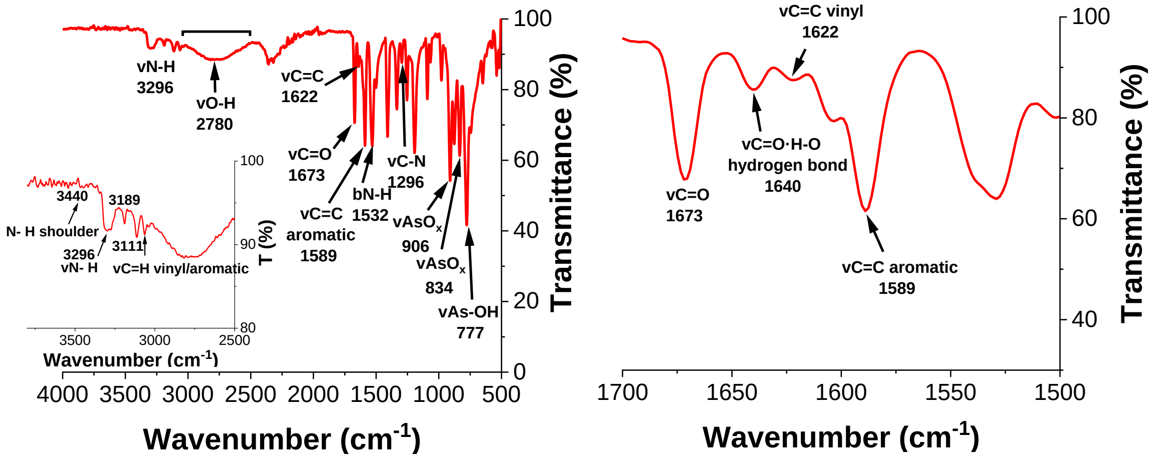

Figure S1C. FT-IR spectrum of 4-(*N*-acrylamido)phenylarsonic acid (**AsAm**) in the range of 500 – 4000 cm^{-1} (left) and 1500 – 1700 cm^{-1} (right).

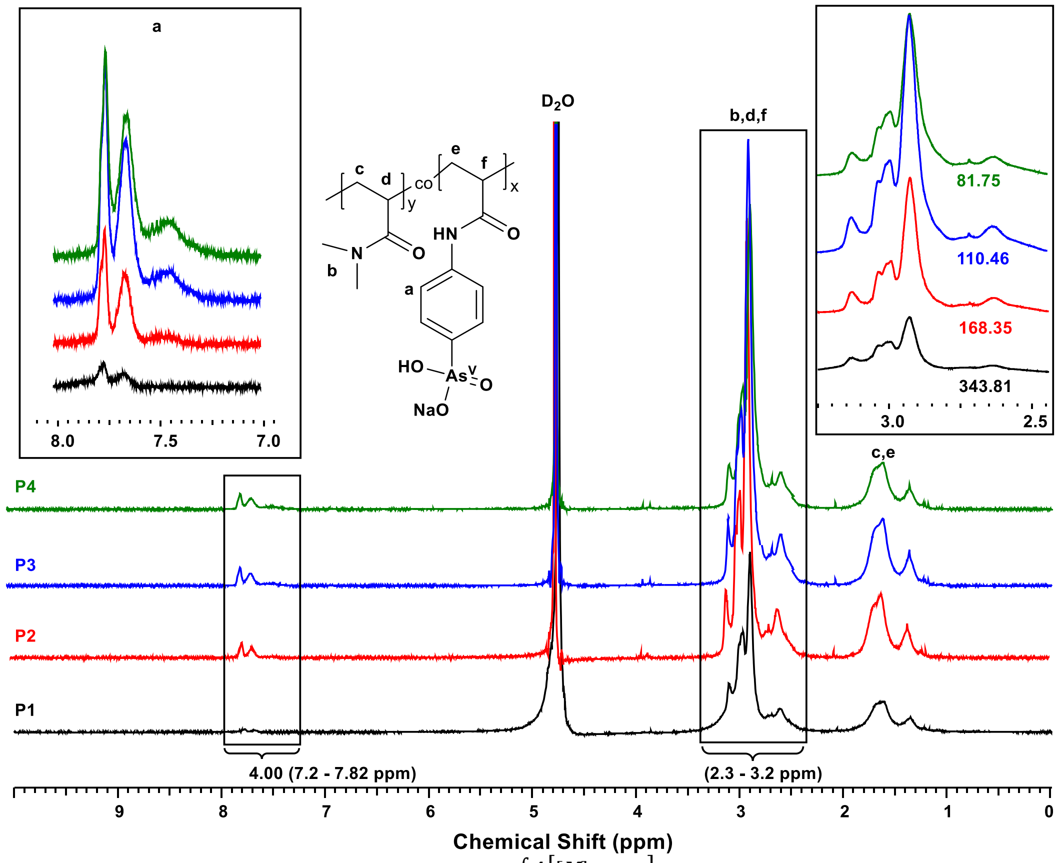

Figure S2A. ^{1}H NMR spectra (400 MHz, D_2O) of **P1 – P4** polymerics arsenical scaffolds. Calculation of AsAm content with respect to DMAm followed the equation:

$$\frac{\int [[M]_{AsAm}+7[M]_{DMAm}]_{2.3-3.2\ ppm} - \int 4[[M]_{AsAm}]_{7.2-7.82\ ppm}}{7} = [M]_{DMAm}$$

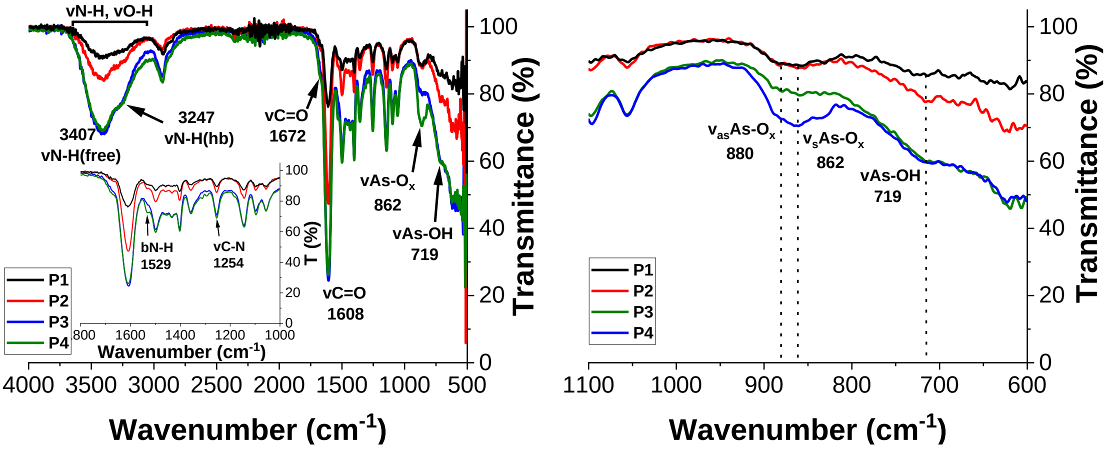

Figure S2B. FT-IR spectra of **P1** – **P4** in the range of 500 – 4000 cm^{-1} (left) and 500 – 1100 cm^{-1} (right). Polymer samples were analysed after direct dialysis and lyophilization, without any further deprotonation.

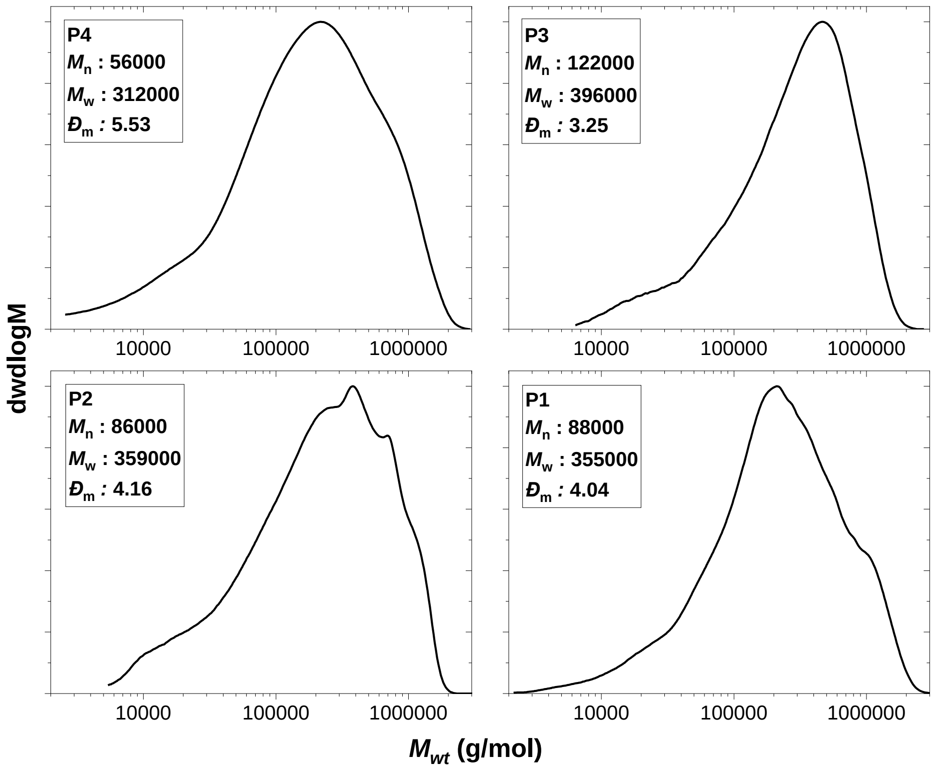

Figure S3. Aqueous SEC of **P1** – **P4** polymeric arsenicals.

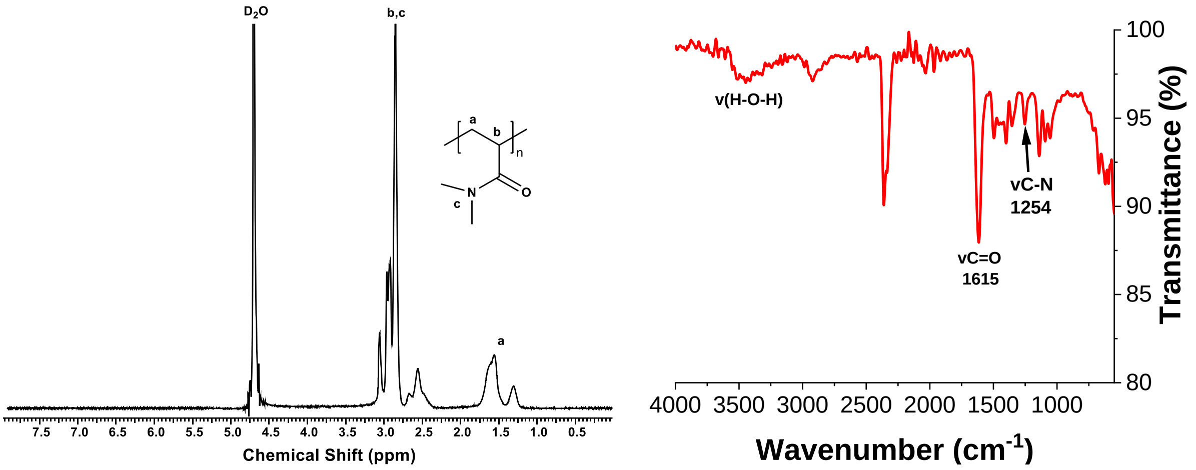

Figure S4A. ^{1}H NMR spectrum (400 MHz, D_2O) of PDMAm / **P5** (left) and FT-IR spectrum in the range of 600 – 4000 cm^{-1} (right).

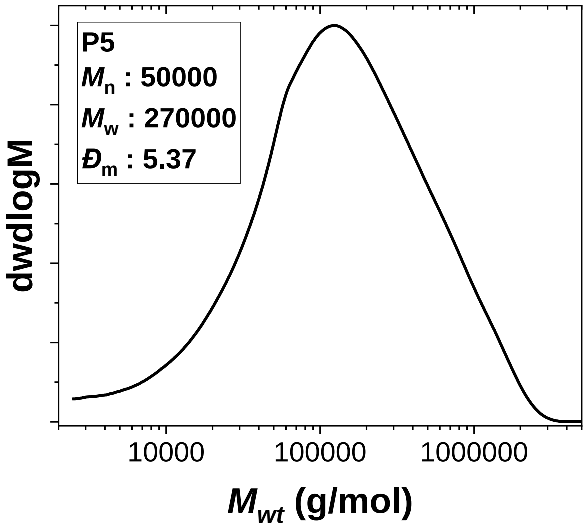

Figure S4B. DMF SEC of PDMAm (**P5**).

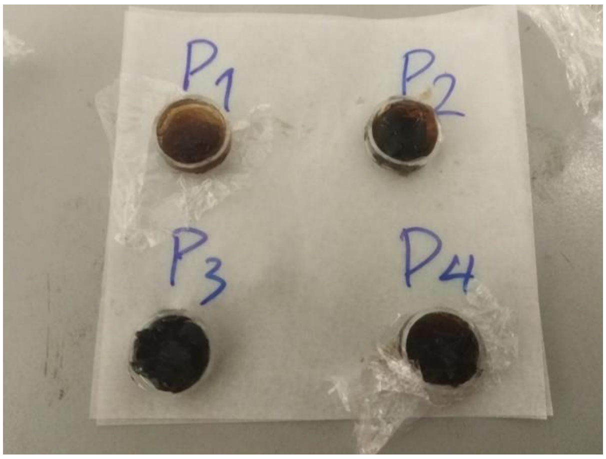

Figure S5. Image of 10 wt% **P1-Pt** – **P4-Pt** hydrogels fabricated at 50 °C.

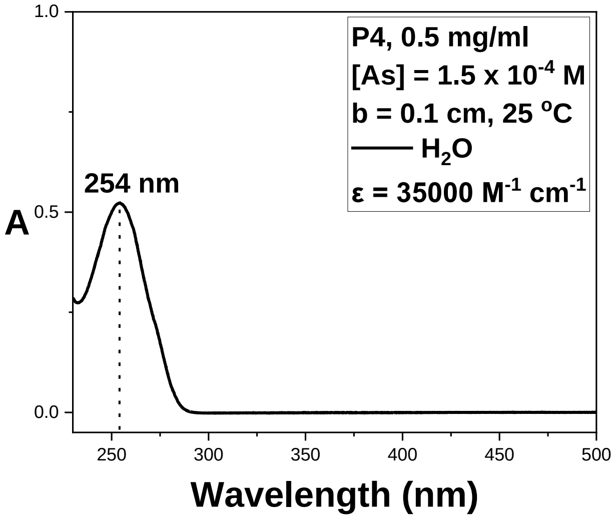

Figure S6. UV-Vis spectrum of **P4** polymer scaffold in H_2O, recorded in the range of 200 – 500 nm. The spectrum show no absorption at $\lambda > 300$ nm. The cell optical path length was b = 0.1 cm.

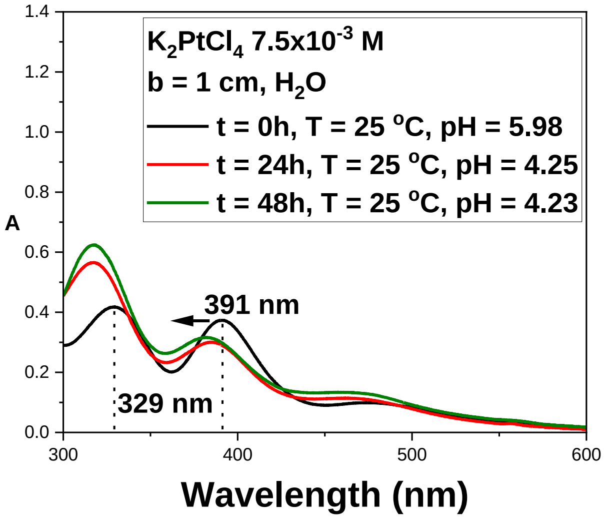

Figure S7. Blue shift of K_2PtCl_4 at λ = 391 nm after 48 h at 25 °C, attributed to ligand exchange ($Cl^- \rightarrow H_2O$) in the cis position. Minimal deprotonation of Pt-water coordinated molecules is indicated by a decrease in solution pH, with no corresponding change in absorption intensity. The cell optical path length was b = 1 cm.

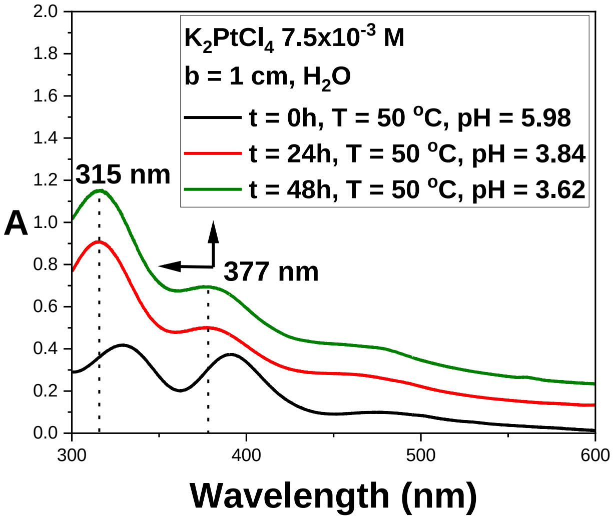

Figure S8A. Blue shift of K_2PtCl_4 at λ = 391 nm after 48 h at 50 °C, attributed to ligand exchange ($Cl^- \rightarrow H_2O$) in the cis position. The deprotonation of Pt-water coordinated indicated by the absorption increase and the decrease in solution pH, is attributed to the formation of $Pt(OH)_2$ at 50 °C. The cell optical path length was b = 1 cm.

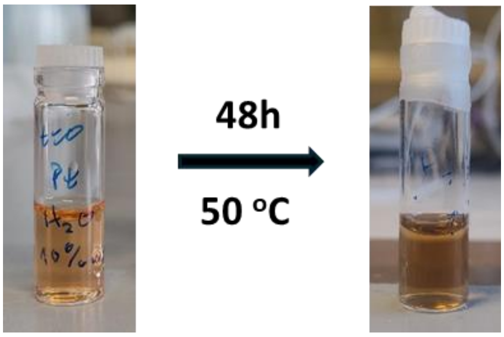

Figure S8B. Images of a 30 mM K_2PtCl_4 solution at t =0 and t = 48h, incubated at 50 °C. The solution darkens over time due to the formation of $Pt(OH)_2$.

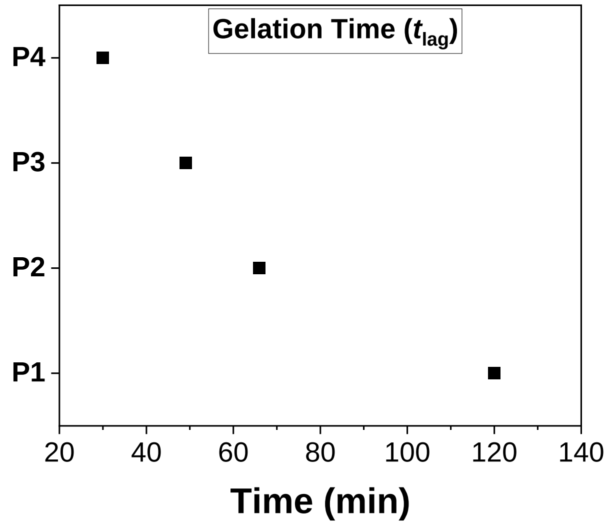

Figure S9A. Gelation time (t_{lag}) at 50 °C for polymer scaffolds with varying AsAm content

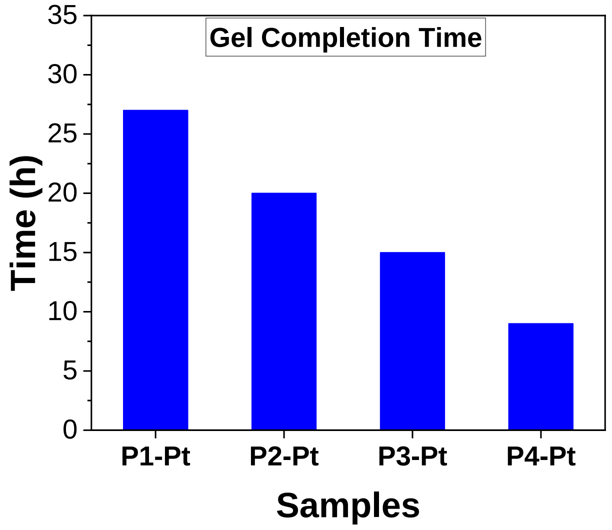

Figure S9B. Gel completion time for different AsAm content hydrogels (**P1-Pt – P4-Pt**)

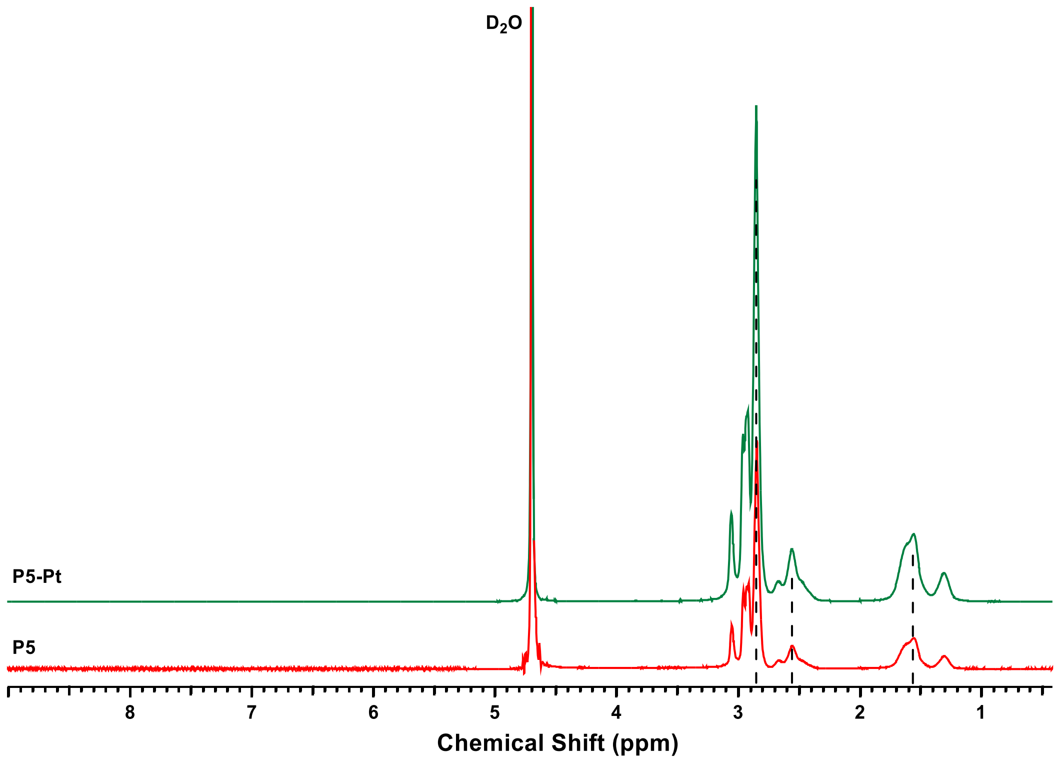

Figure S10. ^{1}H NMR spectra (400 MHz, D_2O) of **P5** and **P5-Pt** showing no shifts in the backbone proton signals upon mixing with K_2PtCl_4.

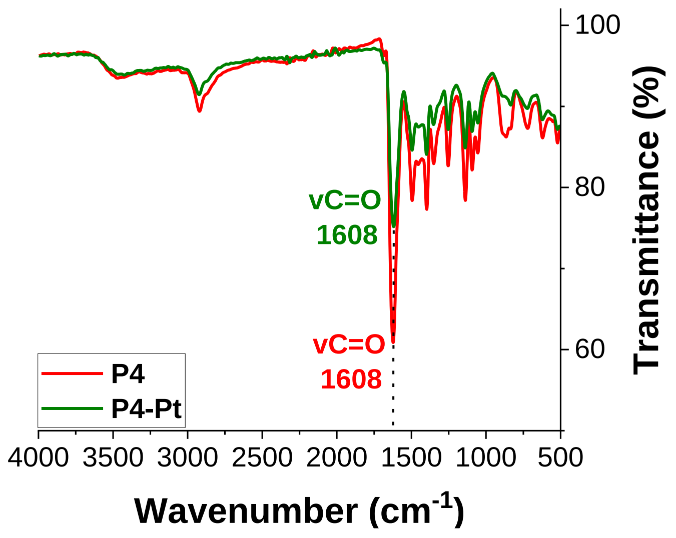

Figure S11. FT-IR of **P4** and **P4-Pt** showing no change to the amide region upon mixing with K_2PtCl_4.

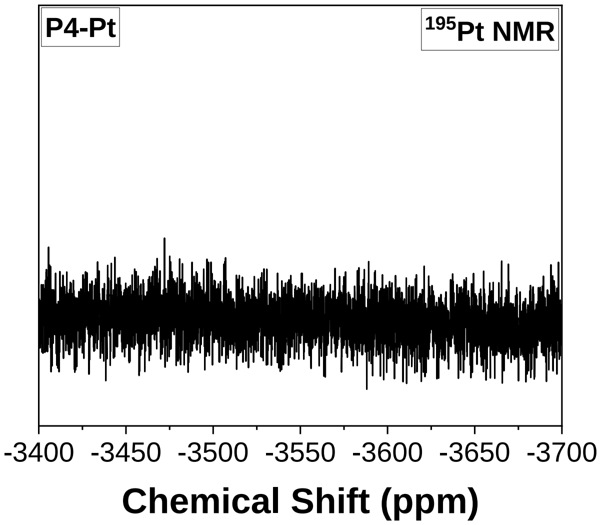

Figure S12. ^{195}Pt NMR spectrum (600 MHz, D_2O) of the **P4-Pt** hydrogel in the range of -3400 to -3700 ppm. No distinctive peak corresponding to the Pt-As bond is observed.

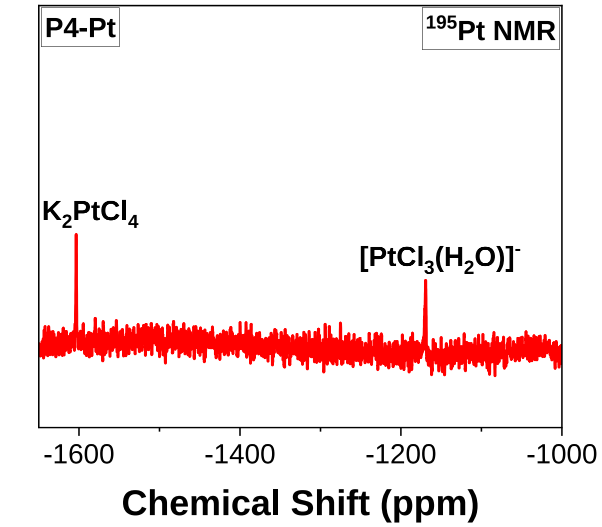

Figure S13. ^{195}Pt NMR spectrum (600 MHz, D_2O) of the **P4-Pt** hydrogel in the range of -1000 to -1650 ppm.

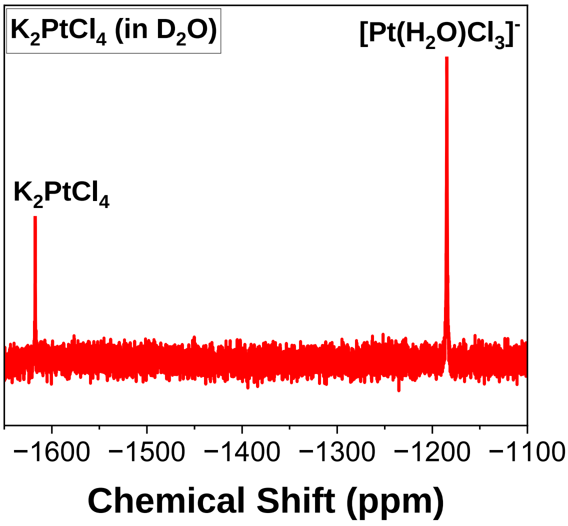

Figure S14. ^{195}Pt NMR spectrum (600 MHz, D_2O) of a 30 mM K_2PtCl_4 solution in D_2O, recorded in the range of -1000 to -1650 ppm.

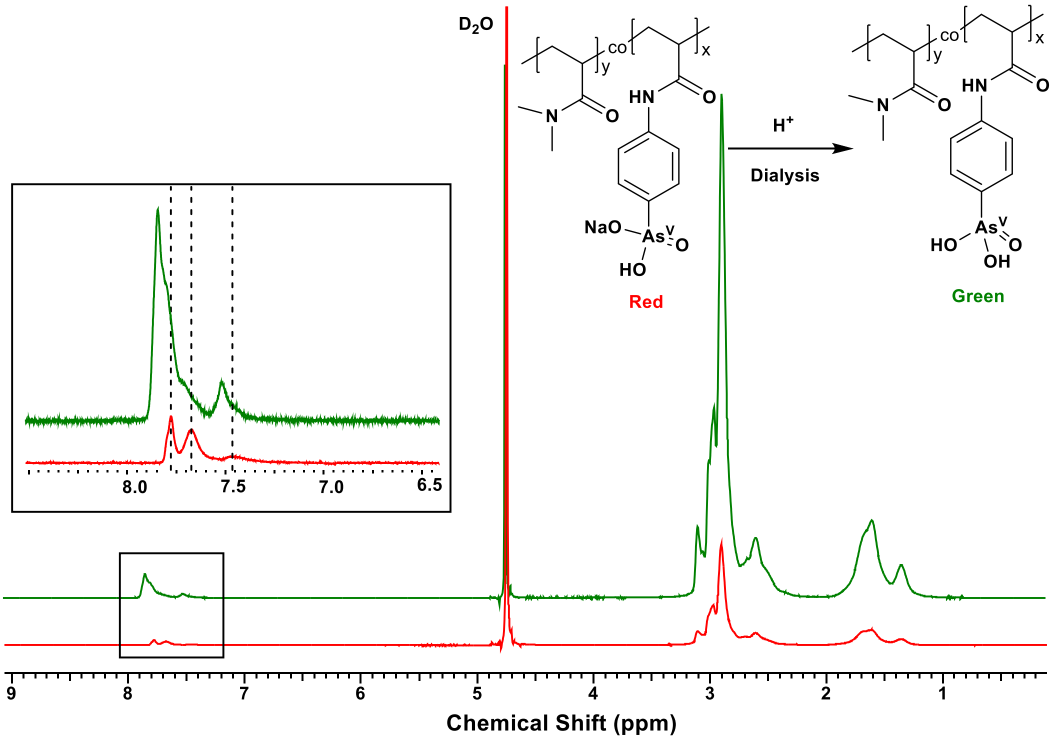

Figure S15A. Downfield shift of aromatic protons in the ^1H NMR spectrum (400 MHz, D_2O) of the **P4'** polymer scaffold, attributed to the complete protonation of arsenic acid groups (AsAm pendants).

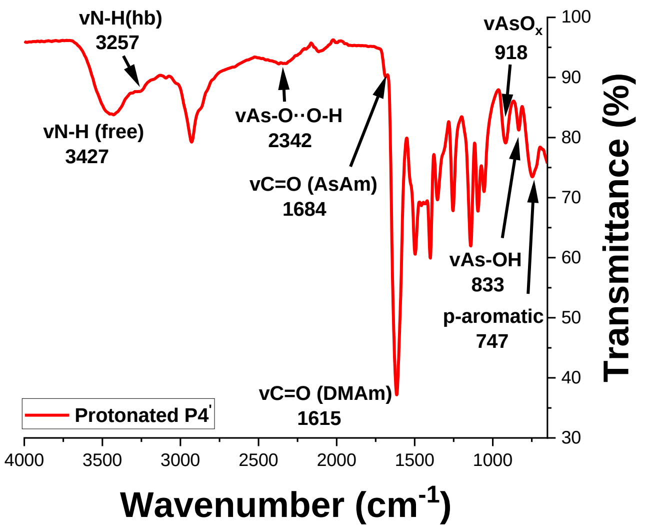

Figure S15B. FT-IR spectrum of fully protonated **P4’** polymer scaffold.

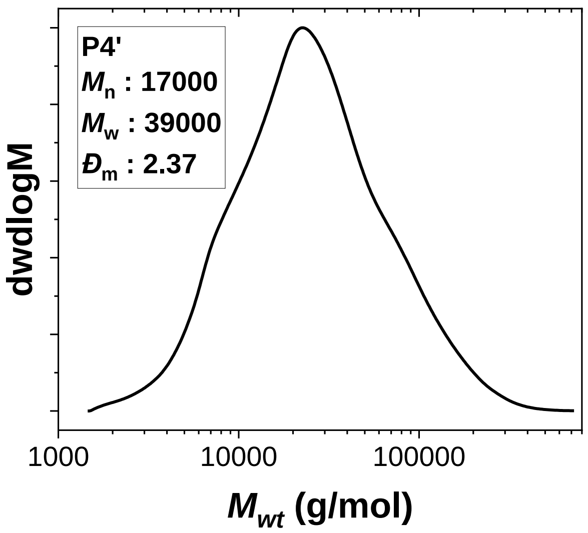

Figure S15C. Aqueous SEC of **P4’** low molecular weight polymeric arsenical scaffold.

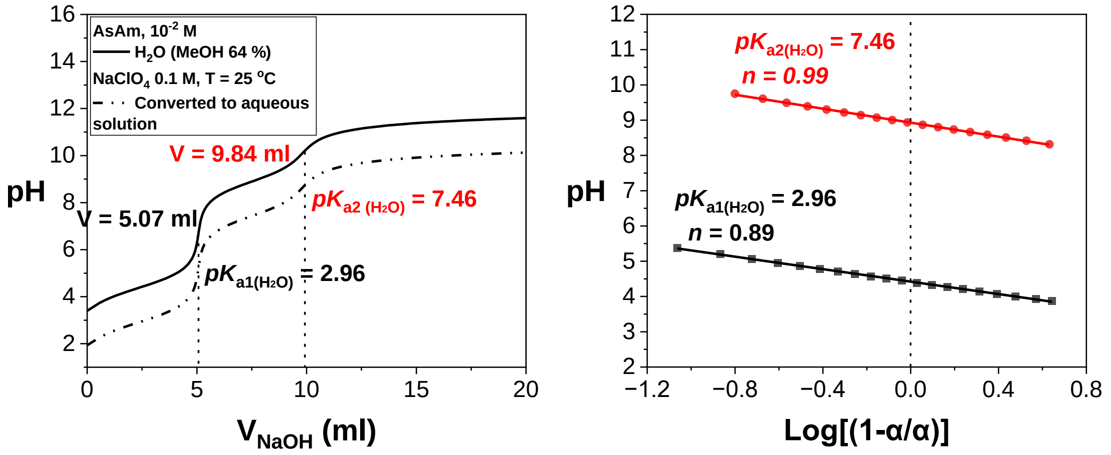

Figure S16. Potentiometric titration of **AsAm** monomer in H_2O (MeOH 64%) with 0.1 M $NaClO_4$. The presence of salt increases monomer solubility due to the salting-in effect. The titration curve is shown on the left, with the corresponding curve converted to aqueous solution using the IUPAC-proposed method (see Experimental Section). Henderson-Hasselbalch plots, using Equation 3, to determine pK_a and n values (right). Both curves were used to calculate the pK_a values, showing absolute agreement.

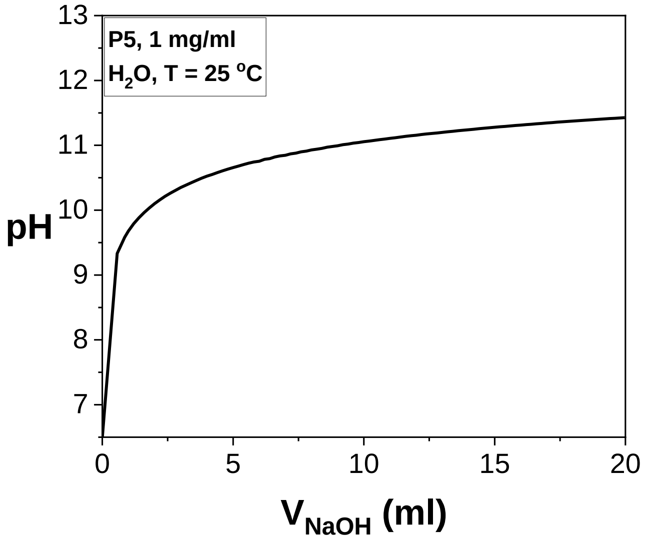

Figure S17. Potentiometric titration of **P5** in H_2O at 25 °C, showing the absence of equivalent point.

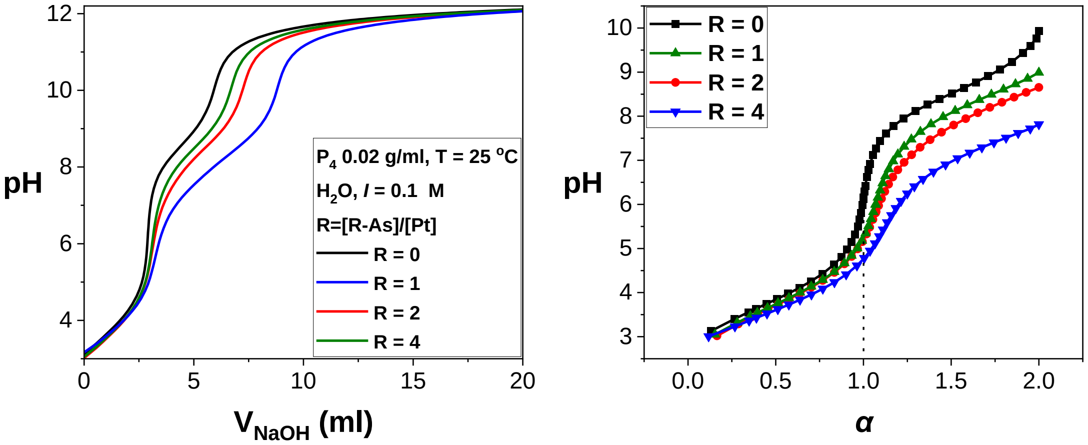

Figure S18. Potentiometric titrations of **P4'** in H_2O and 0.1 M $NaClO_4$ in the absence and presence of K_2PtCl_4 with [As]/[Pt] ratios of 1 – 4 (left). A drop in solution pH is observed with the progress of deprotonation-complexation upon addition of Pt^{II} ([As]/[Pt] = 1 – 4) at the first part of the pH = f(α) curve (right).

Table S1. Decrease in pH at different neutralization points for a **P4'** solution in the presence and absence of inert electrolyte ($NaClO_4$) upon the addition of 30 mmol Pt^{II}.

**Sample**	***α***	**Δ*pH***
0.02 g/ml **P4'** in DI water (I = 0)	0	0.12
	0.25	0.15
	0.5	0.19
0.02 g/ml **P4'** in DI water in presence of 0.1 M $NaClO_4$ (I = 0.1 M)	0	0.13
	0.25	0.18
	0.5	0.23

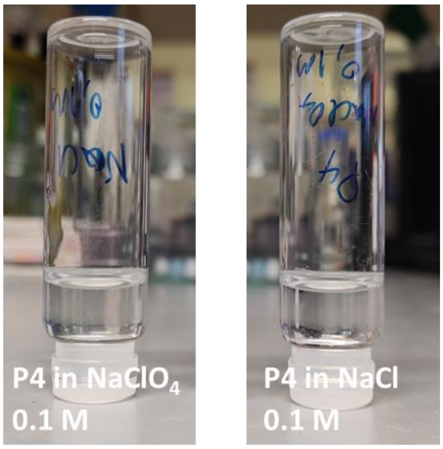

Figure S19A. 10 wt% solution of **P4** in the presence of 0.1 M $NaClO_4$ (right) and 0.1 M NaCl (left) after overnight incubation at 50 °C. The inert electrolytes act as balancing ions, failing to form a gel network.

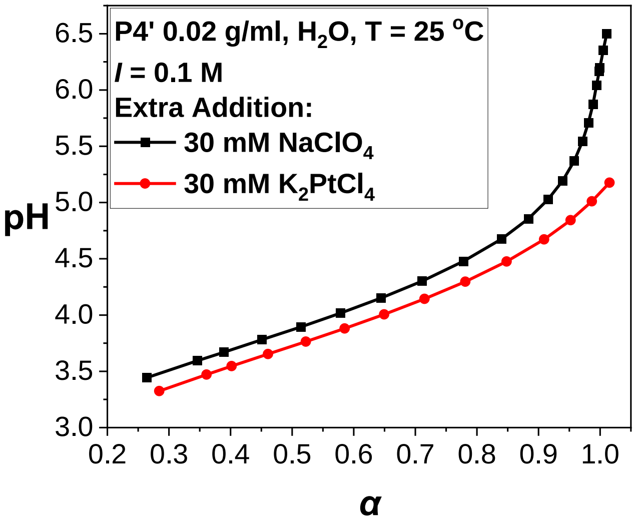

Figure S19B. pH change of a **P4'** solution with I = 0.1 M upon the addition of equivalent amounts of Pt^{II} and Na^+.

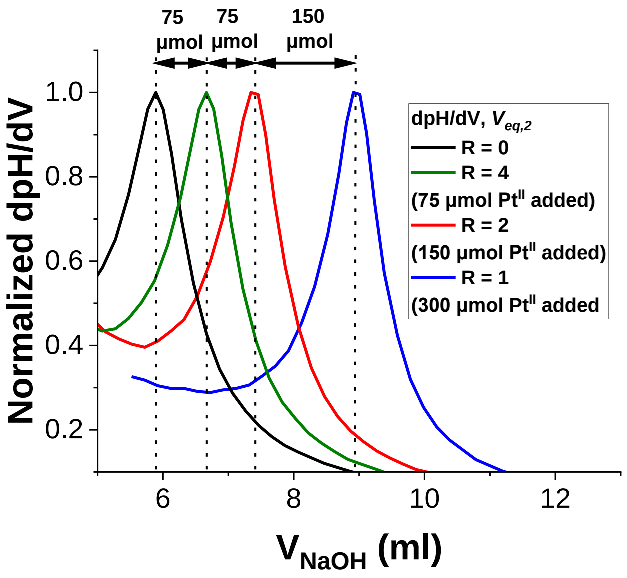

Figure S20. Positive shift of the second equivalence point ($V_{eq,2}$) of **P4'** directly proportional to the concentration of Pt^{II} added.

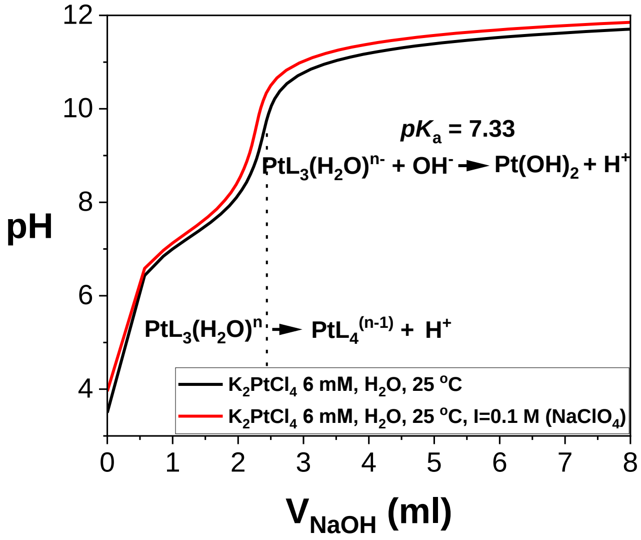

Figure S21. Potentiometric titration of 6 mM K_2PtCl_4 solution in presence and absence of electrolyte ($NaClO_4$ 0.1 M).

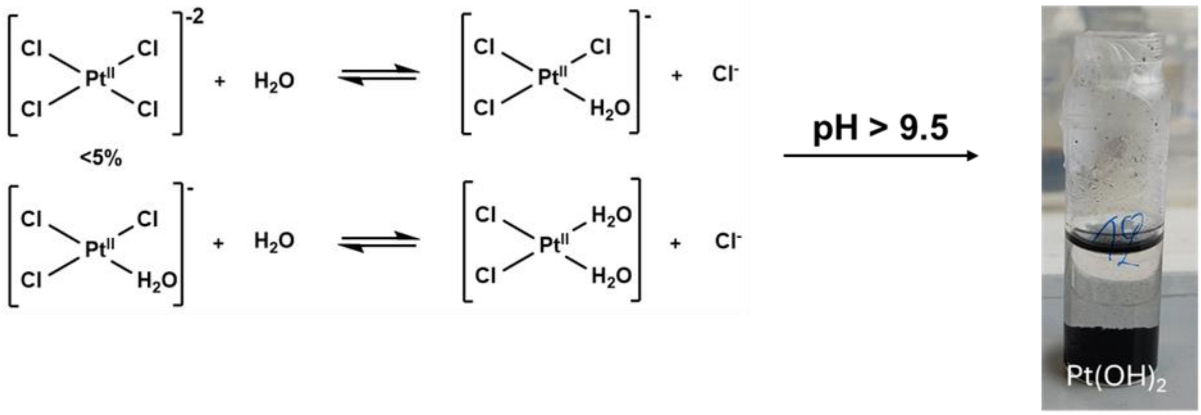

Figure S22. Water ligand exchange in aqueous solution of K_2PtCl_4 (30 mM) and precipitation of the dark $Pt(OH)_2$ after deprotonation at pH > 9.5.

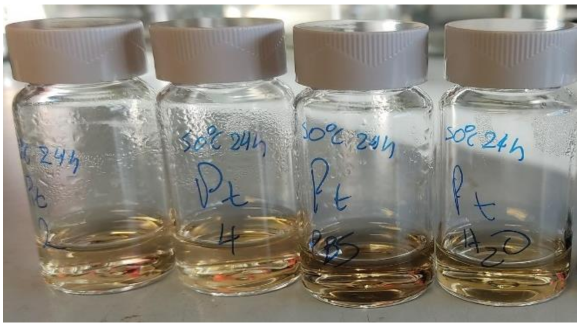

Figure S23A. Image of K_2PtCl_4 solutions (7.5 mM) in different pH media incubated at 50 °C for 24 hours. Solutions: pH = 2, pH = 4, PBS (1X, pH = 7.4) and H_2O (left to right). The solution darkens over time due to the formation of $Pt(OH)_2$, but no precipitation is observed at pH < 7.

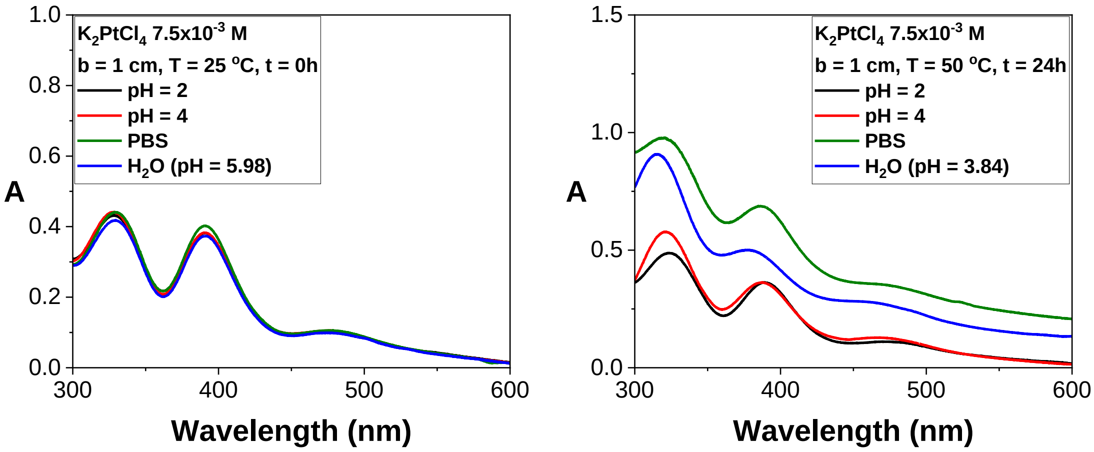

Figure S23B. UV-Vis spectra of K_2PtCl_4 solutions (7.5 mM) in different pH media, recorded at t = 0 (left) and after 24 hours (right) of incubation at 50 °C. A small increase in absorption is observed over time, predominantly near pH = 7. The cell optical path length was b = 1 cm.

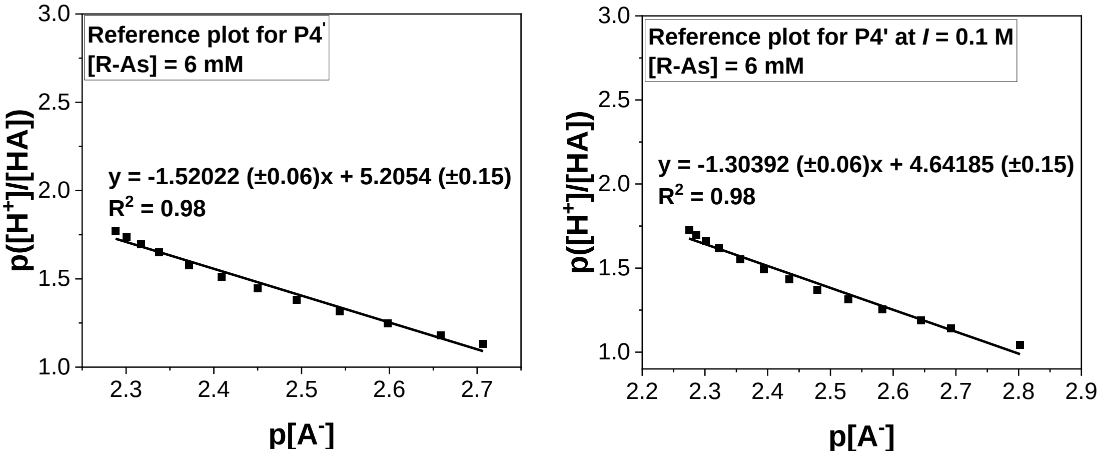

Figure S24. Reference plot p([H^+]/HA]) vs p[A^-] for **P4'** (0.02 g/ml) in DI water with no added electrolyte (left). At I = 0.1 M (right).

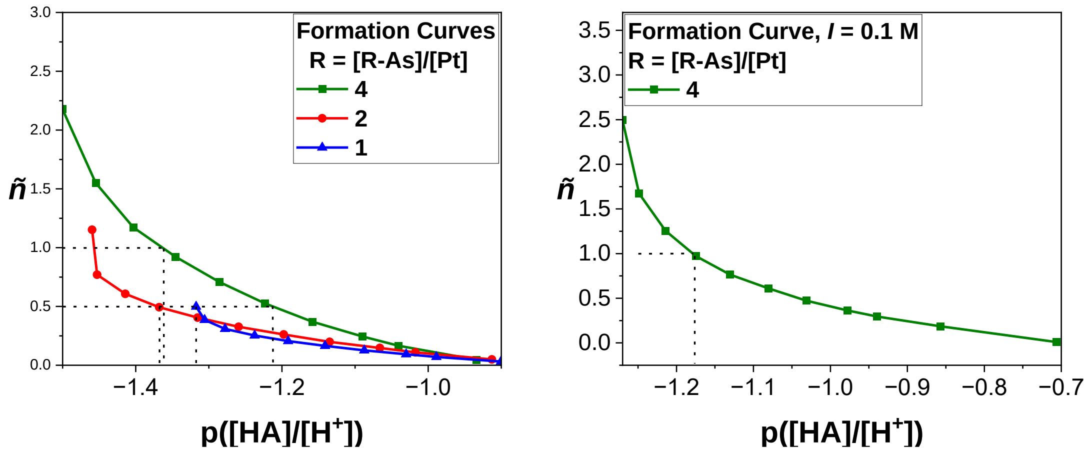

Figure S25. Typical formation curve $\tilde{n}$ vs p([HA]/H^+]) for **P4'/Pt** in DI water with no added electrolyte ([As]/[Pt] = 1 – 4, left). At I = 0.1 M ([As]/[Pt] = 4, right).

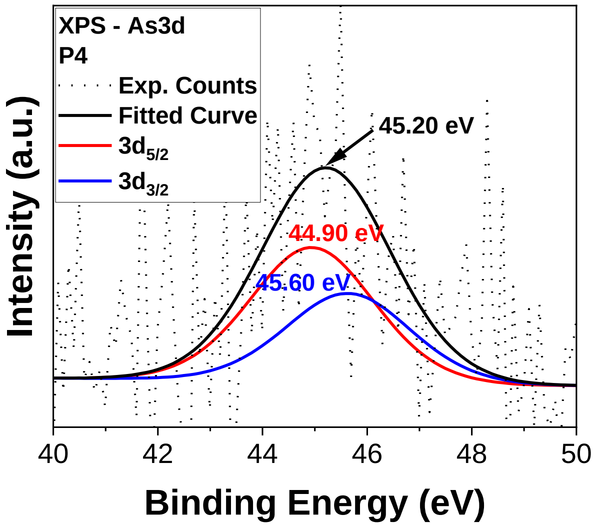

Figure S26. As3d XPS spectrum of **P4** polymer, with the peak deconvoluted into $3d_{5/2}$ and $3d_{3/2}$ components.

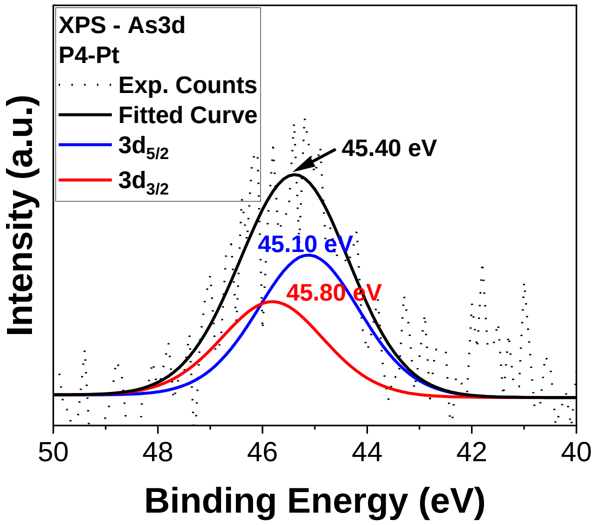

Figure S27. As3d XPS spectrum of dried **P4-Pt** gel, with the peak deconvoluted into $3d_{5/2}$ and $3d_{3/2}$ components.

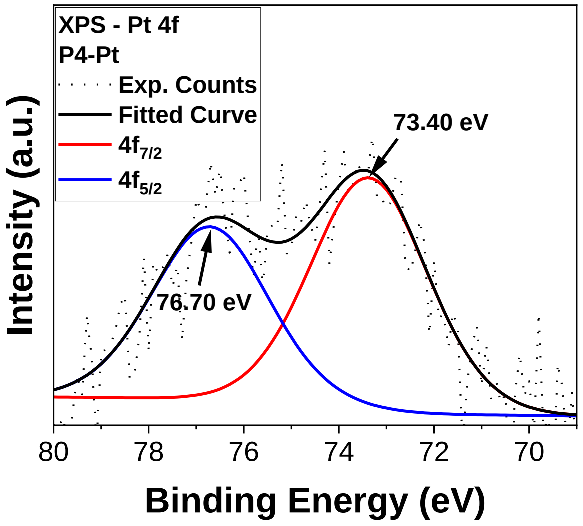

Figure S28. Pt4f XPS spectrum of dried **P4-Pt** gel, with the peak deconvoluted into $4f_7/_2$ and $3d_5/_2$ components.

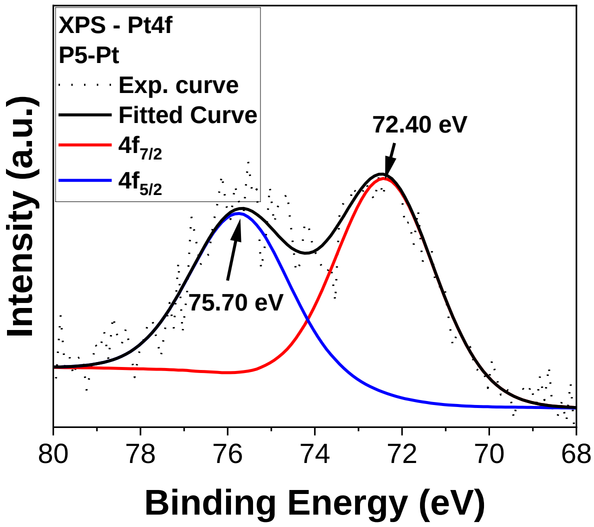

Figure S29. Pt4f XPS spectrum of dried **P5-Pt** mixture (PDMAm with 30 mM of K_2PtCl_4), with the peak deconvoluted into $4f_7/_2$ and $3d_5/_2$ components.

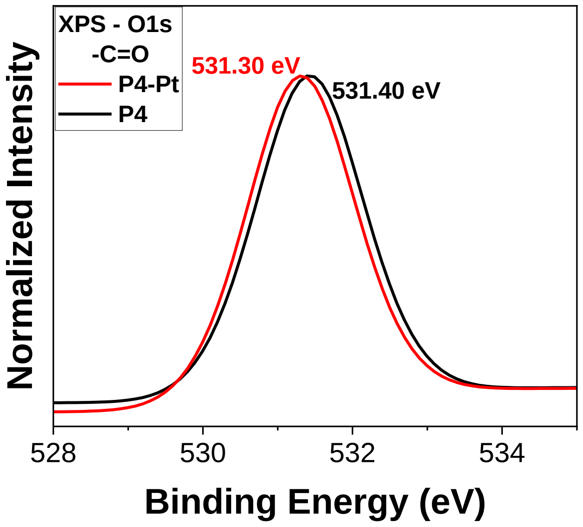

Figure S30A. Deconvoluted XPS peaks of the -C=O group for the **P4** polymer and dried **P4-Pt** gel. The spectra show minimal shift for the amidic oxygen after gelation.

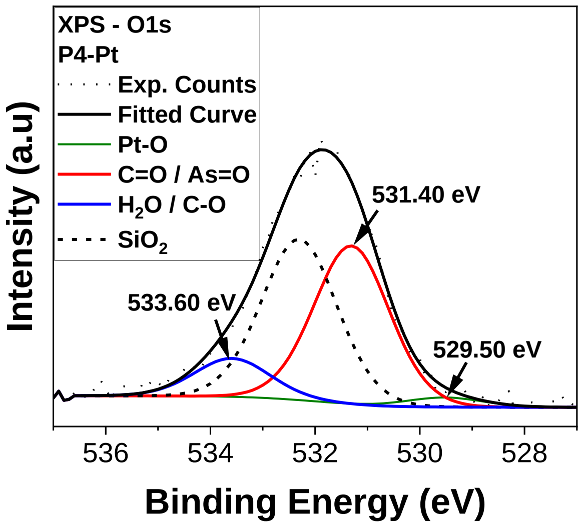

Figure S30B. O1s XPS spectrum of dried **P4-Pt** gel with deconvoluted corresponding components.

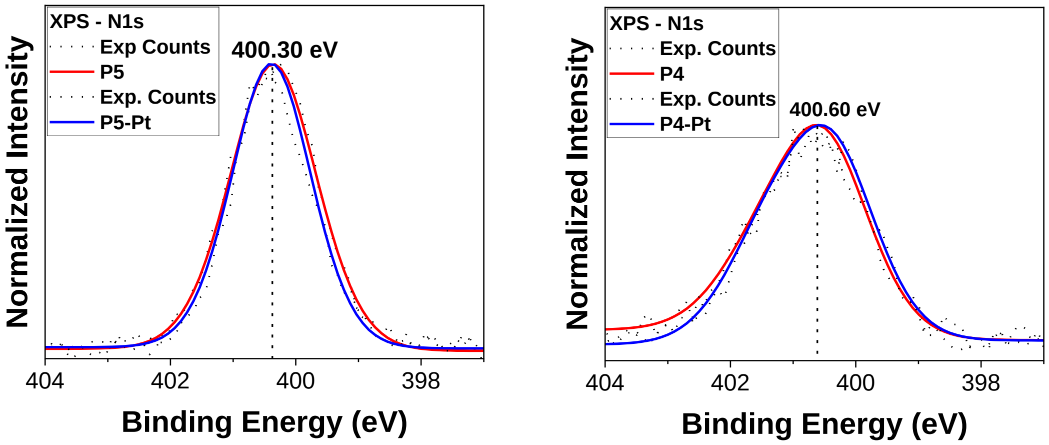

Figure S31. N1s XPS spectra of **P5** and dried **P5-Pt** mixture (PDMAm with 30 mM K_2PtCl_4) on the left, and **P4** and dried **P4-Pt** gel on the right. No peak shift is observed after reaction with Pt^{II}.

Table S2. Atomic concentrations (%) of key elements in **P4-Pt** determined by X-ray photoelectron spectroscopy (XPS).

Sample	O	N	C	Pt	As	Cl
**P4**	13.93	7.84	75.75	0.00	0.46	0.00
**P4-Pt**	14.50	11.59	68.69	0.29	0.67	1.95

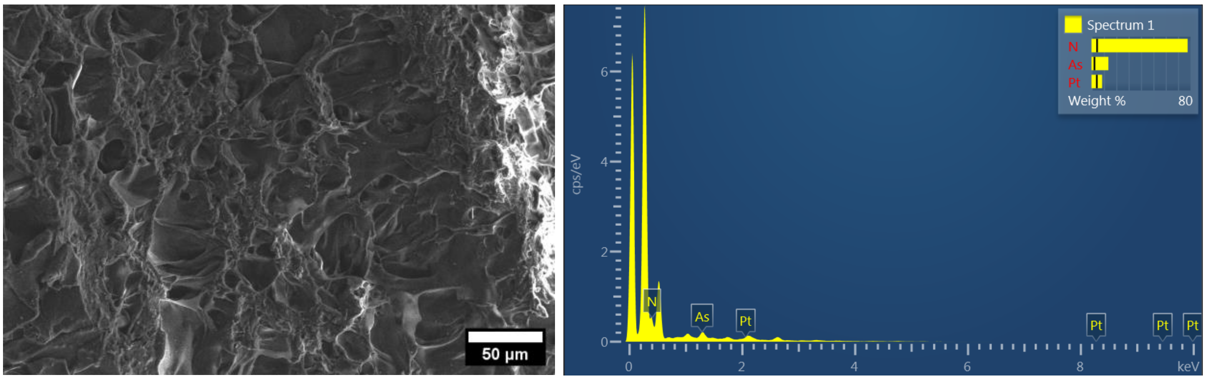

Figure S32A. SEM/EDX spectrum of the corresponding **P1-Pt** dried gel image.

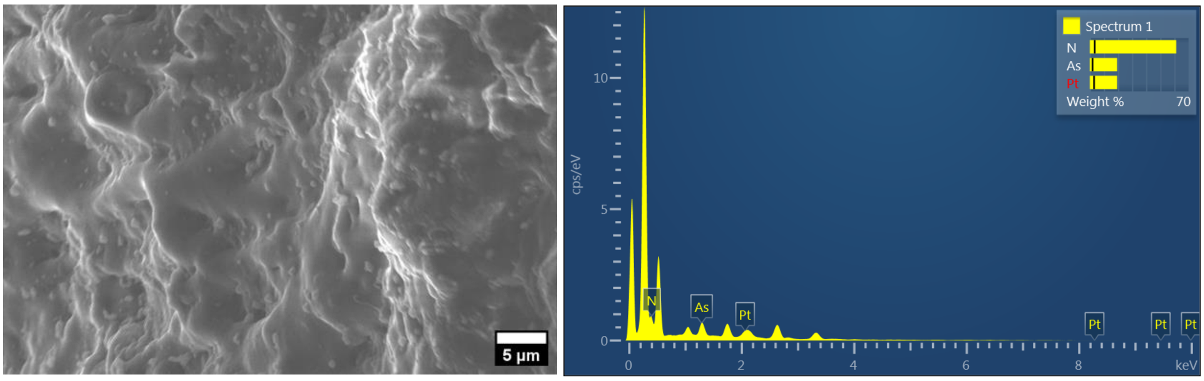

Figure S32B. SEM/EDX spectrum of the corresponding **P2-Pt** dried gel image.

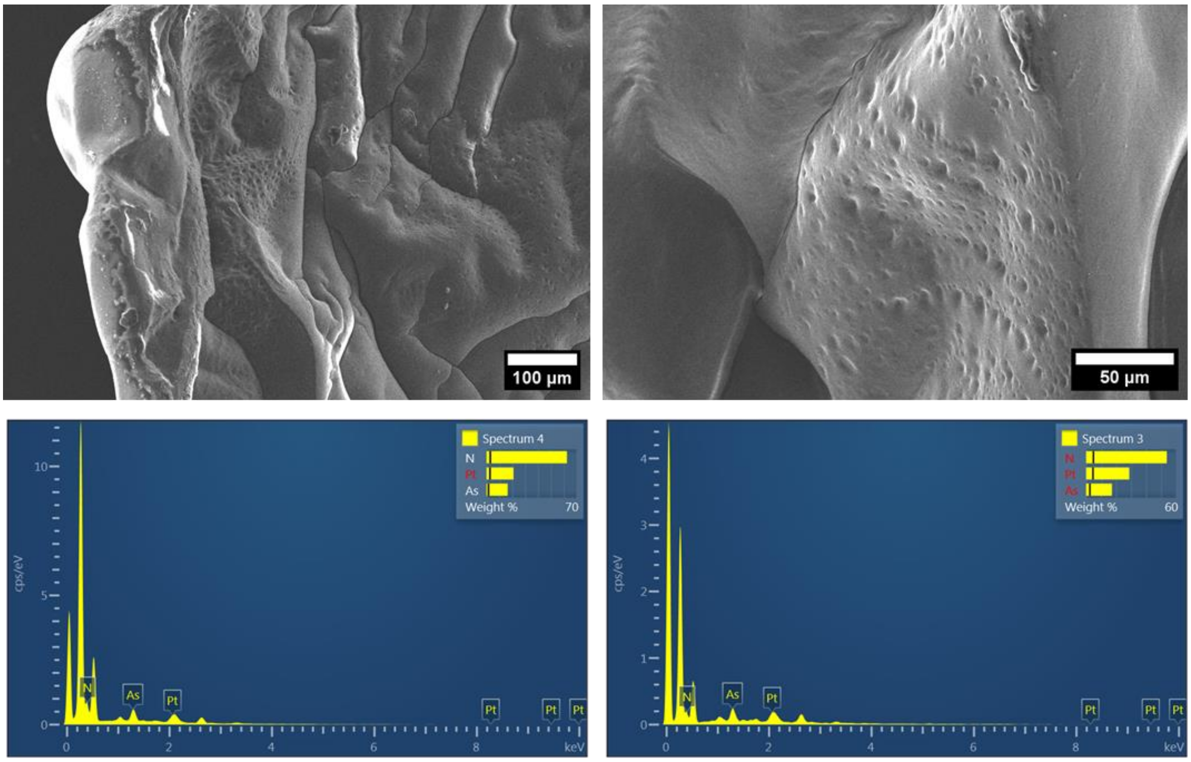

Figure S32C. SEM/EDX spectrum of the corresponding **P3-Pt** dried gel images.

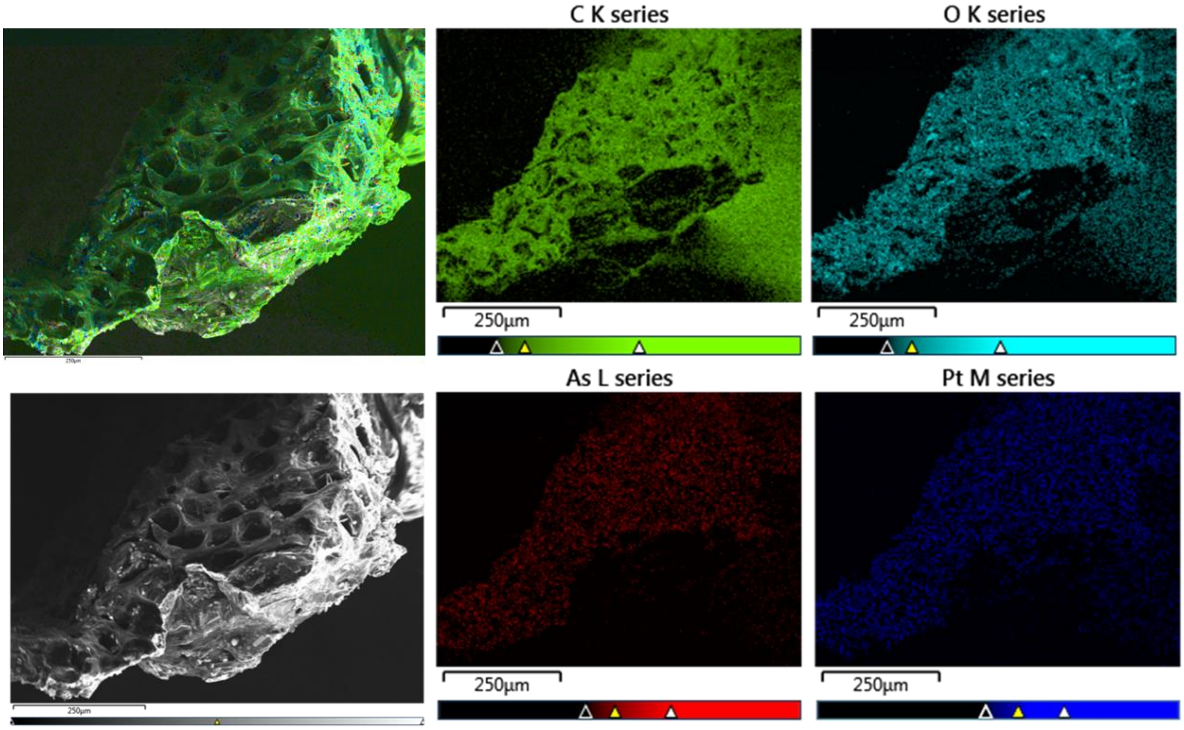

Figure S32D. SEM/EDX mapping analysis for the **P4-Pt** dried gel, showing the distribution of As and Pt on the gel's surface.

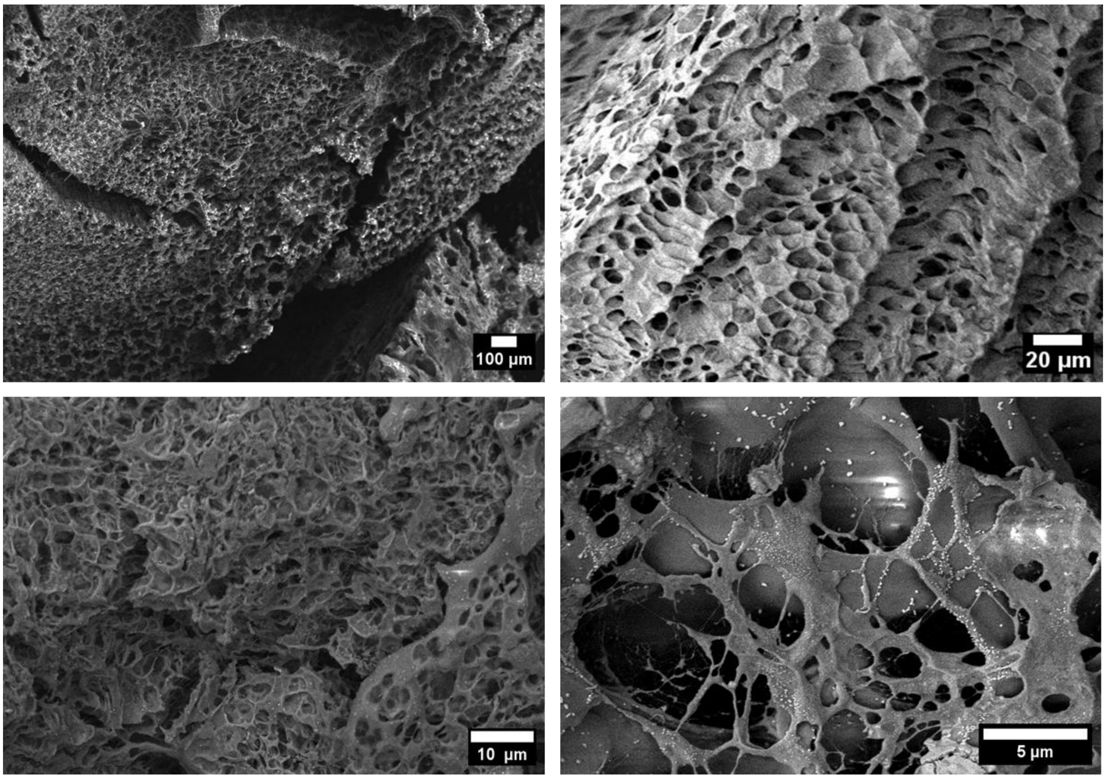

Figure S32E. Scanning electron microscopy (SEM) images of P4-Pt dried gel, showing surface morphology and pore structure. Scale bars represent 100, 20, 10 and 5 µm.

Table S3. Rheological properties of **P1-Pt** – **P4-Pt** gels. Storage modulus (*G'*) and loss modulus (*G''*) were determined from frequency sweep experiments at ω = 1 rad/s and ω = 100 rad/s.

**Hydrogel**	***G'* (Pa), ω = 1 rad/s**	***G'* (Pa), ω = 100 rad/s**
**P1-Pt**	286 ± 109	527 ± 125
**P2-Pt**	310 ± 77	398 ± 93
**P3-Pt**	320 ± 40	395 ± 40
**P4-Pt**	333 ± 19	399 ± 37

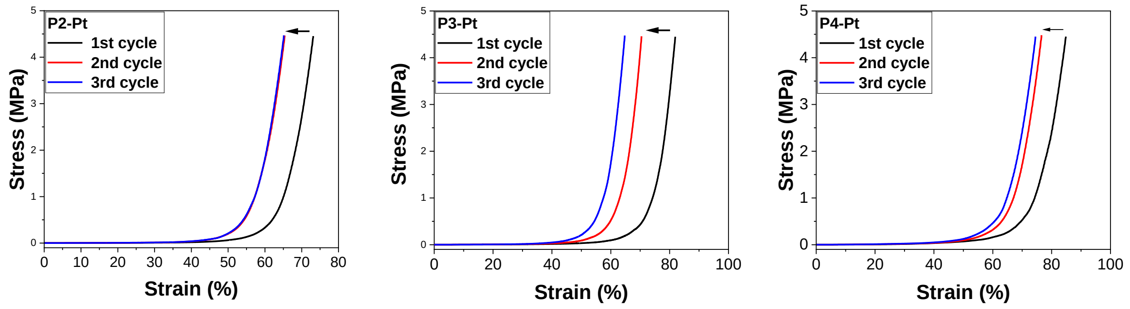

Figure S33. Consecutive stress-strain curves on **P2-Pt**, **P3-Pt** and **P4-Pt** hydrogels (left to right) obtained from cyclic compression tests.

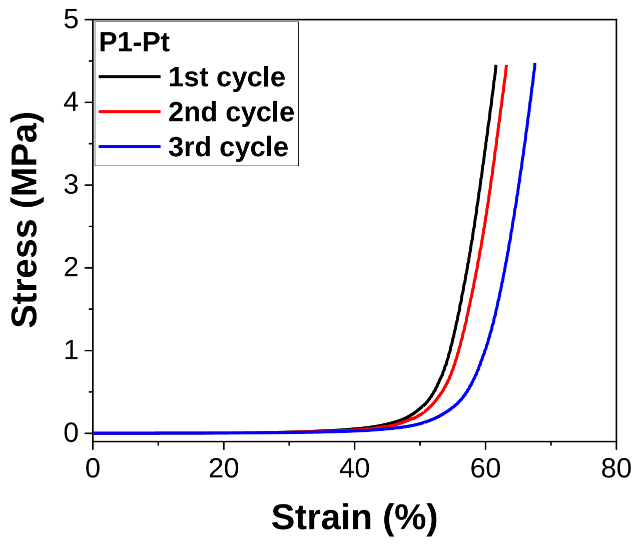

Figure S34. Consecutive stress-strain curves on **P1-Pt** hydrogel obtained from cyclic compression tests.

Table S4. Swelling ratios' of **P1-Pt** – **P4-Pt** and **P6-Pt** as a function of time investigated in DI water. The swelling ratios' are expressed as the degree of swelling $(W_s - W_0)/W_0 \times 100$, rounded to the nearest 5 or 10.

Hydrogel	24 h	48 h	72 h	96 h	120 h	144 h	168 h
**P1-Pt**	250	835	1155	1195	-	-	-
**P2-Pt**	445	1105	1485	1730	1695	-	-
**P3-Pt**	1240	1755	1935	2170	2365	2420	2425
**P4-Pt**	935	1310	1505	1650	1785	1885	1875
**P6-Pt**	235	355	475	465	-	-	-

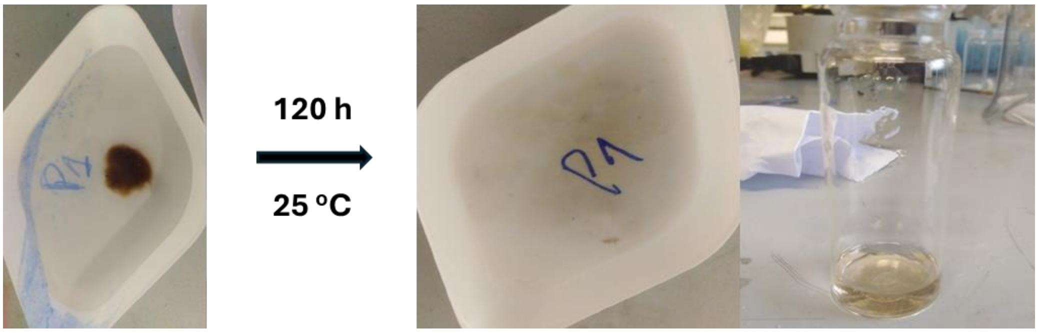

Figure S35. Images of **P1-Pt** hydrogel at t = 0 and after 120 hours of swelling in DI water.

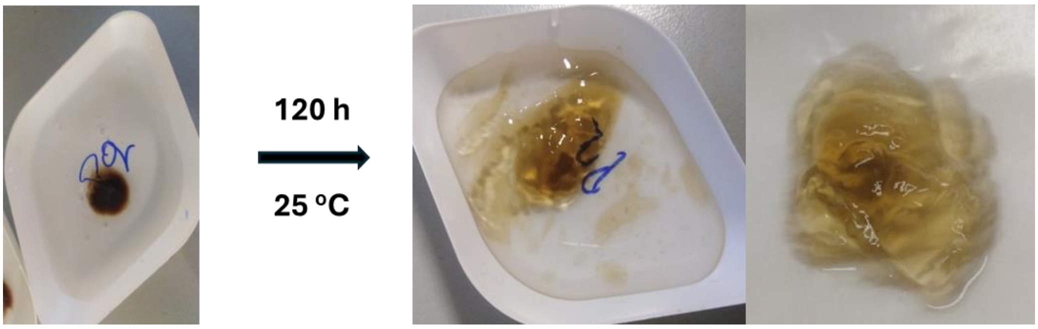

Figure S36. Images of **P2-Pt** hydrogel at t = 0 and after 120 hours of swelling in DI water.

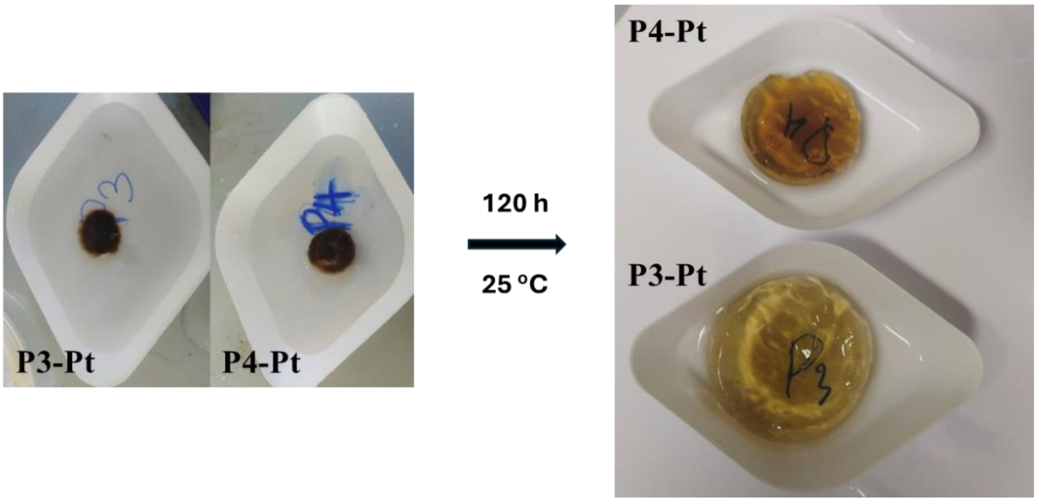

Figure S37. Images of **P3-Pt** and **P4-Pt** hydrogel at t = 0 and after 120 hours of swelling in DI water. Comparison of the sizes of **P3-Pt** and **P4-Pt** samples at swelling equilibrium.

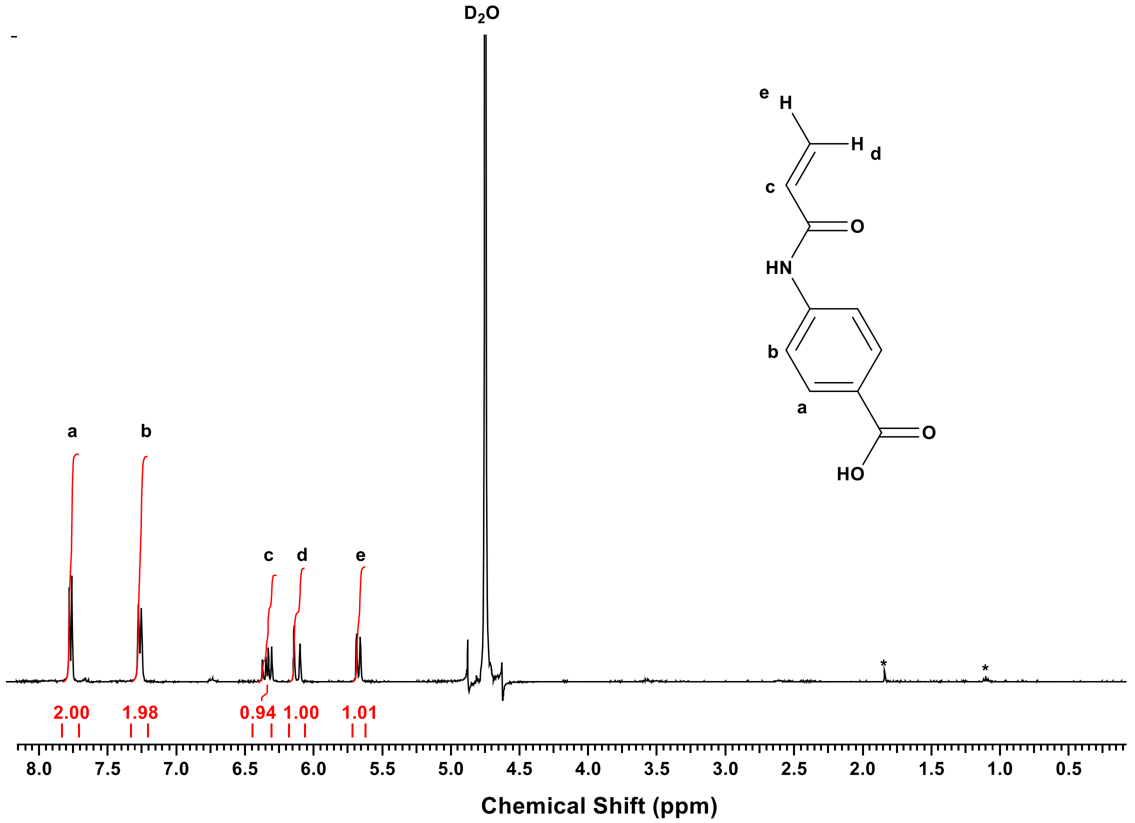

Figure S38A. ^{1}H NMR spectrum (400 MHz, D_2O/NaOH 1 M) of 4-acrylamidobenzoic acid. *EtOAc impurities.

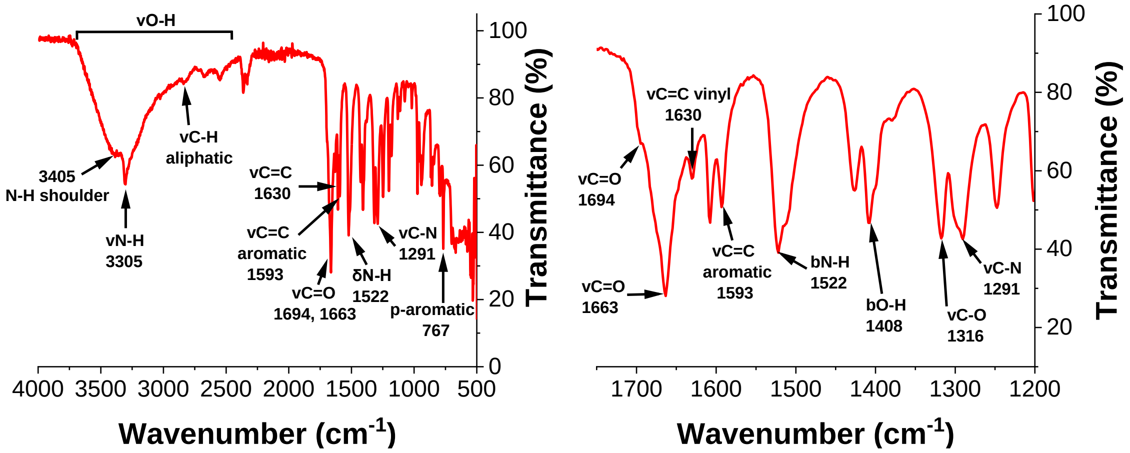

Figure S38B. FT-IR spectrum of 4-acrylamidobenzoic acid in the range of 500 – 4000 cm^{-1} (left) and 1200 – 1750 cm^{-1} (right).

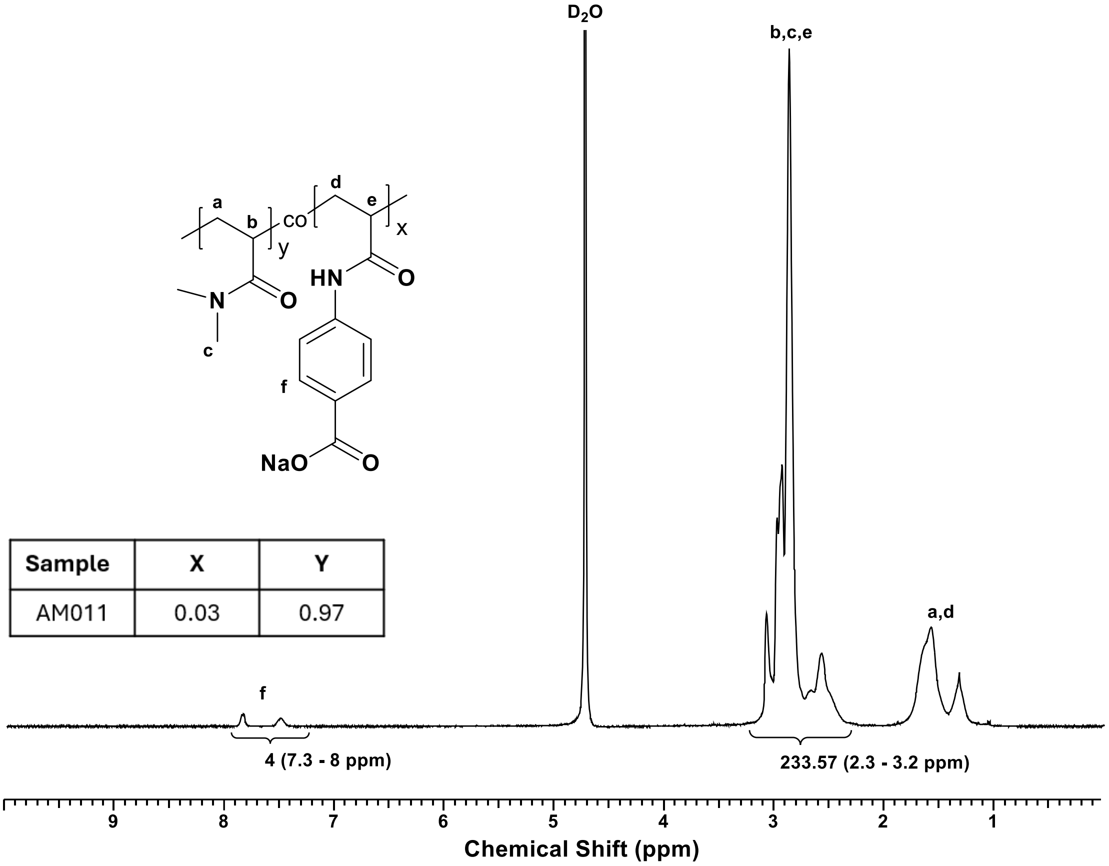

Figure S39A. ^{1}H NMR spectrum (400 MHz, D_2O) of **P6** copolymer of DMAm and 4-acrylamidobenzoic acid.

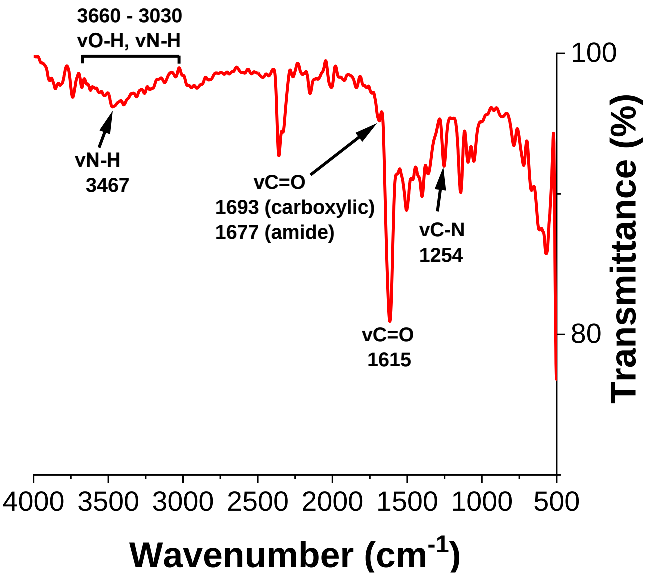

Figure S39B. FT-IR spectrum of **P6** copolymer with DMAm and 4-acrylamidobenzoic acid in the range of 500 – 4000 cm^{-1}.

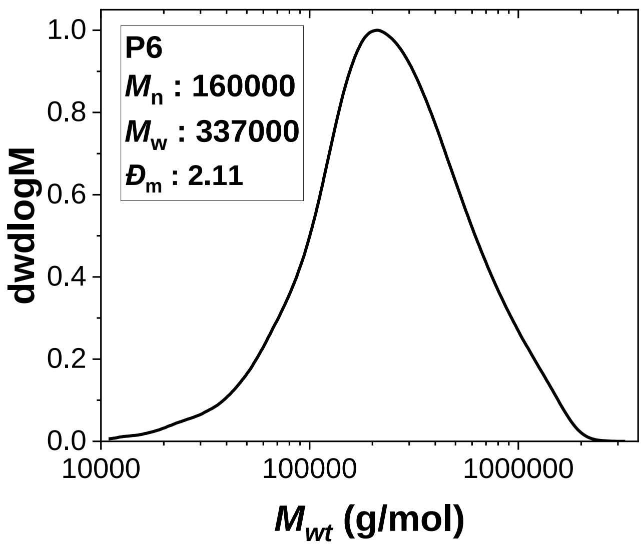

Figure S40. Aqueous SEC of **P6** copolymer with DMAm and 4-acrylamidobenzoic acid.

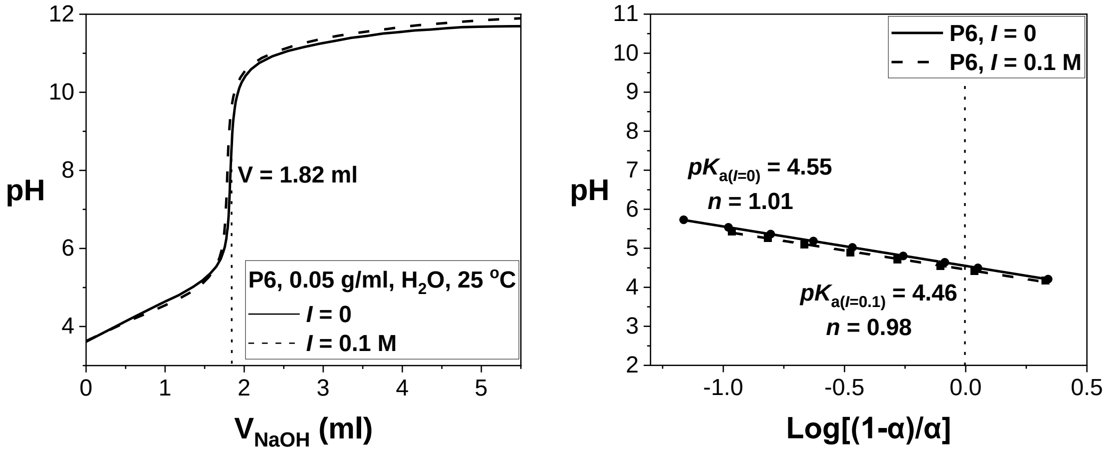

Figure S41. Titration curves of **P6** in H_2O (solid line) and 0.1 M $NaClO_4$ (dashed) on the left. Corresponding Henderson-Hasselbalch plots, using Equation 3, to determine pK_a and n values on the right. The pK_a in H_2O ($I = 0$) was determined to be 4.55.

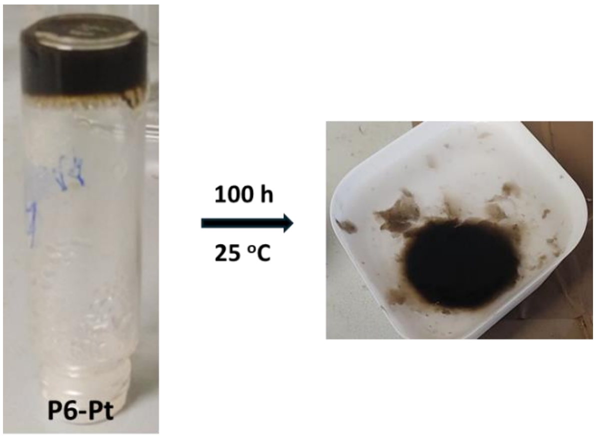

Figure S42. Images of **P6-Pt** hydrogel at t = 0 and after 100 hours of swelling in DI water. The material immediately begins to disintegrate, eventually breaking into pieces after 4 days.

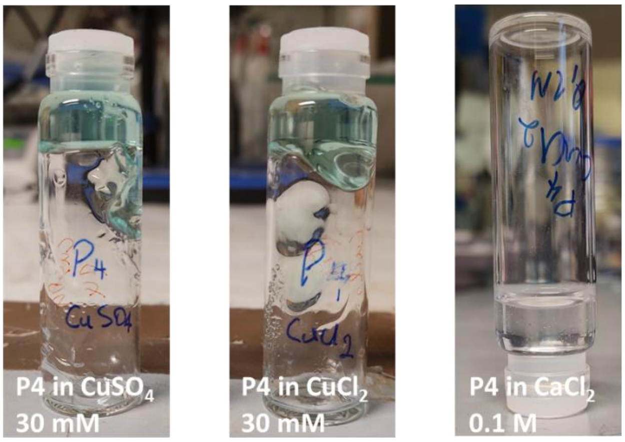

Figure S43. 10 wt% solution of **P4** in the presence of 30 mM $CuSO_4$, $CuCl_2$ and 0.1 M $CaCl_2$. The hard Ca^{II} ions act as balancing ions, failing to form a gel even after overnight incubation at 50 °C. In contrast, the softer Cu^{II} ions rapidly form a stable hydrogel within 30 minutes of mixing at 25 °C.

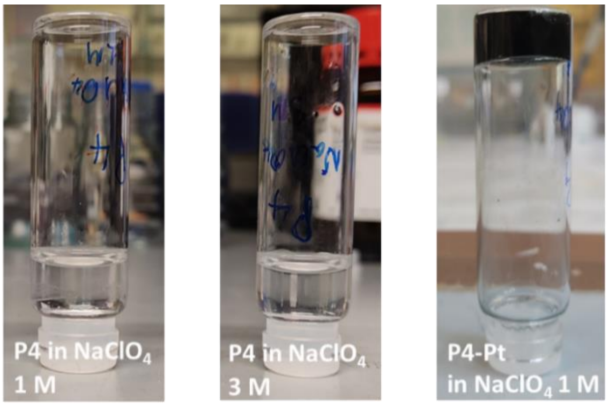

Figure S44. 10 wt% solution of **P4** in the presence of 1 M and 3 M $NaClO_4$ (left). Formation of **P4-Pt** gel in 1 M $NaClO_4$ aqueous media (right). The presence of a high concentration of hard, monovalent Na^+ ions does not inhibit gel formation with Pt^{II}.

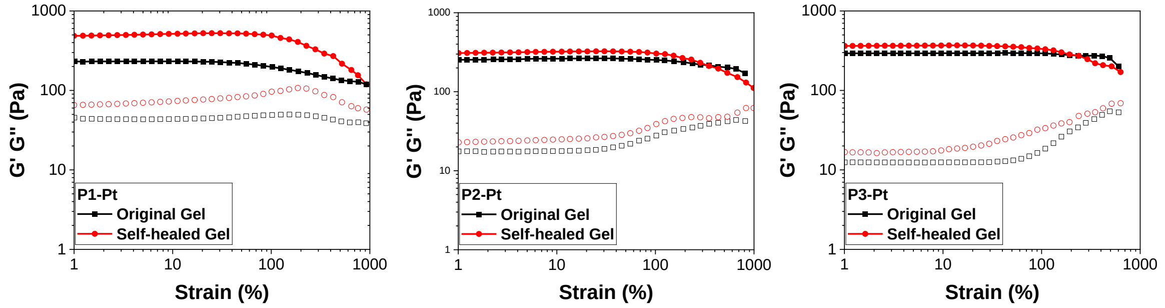

Figure S45. Cycled amplitude sweeps for **P1-Pt**, **P2-Pt**, and **P3-Pt** at 25 °C and $\omega = 10$ rad s^{-1} before and after 1 hour healing in a humidity chamber. All gels successfully regained comparable rheological characteristics after the healing process.

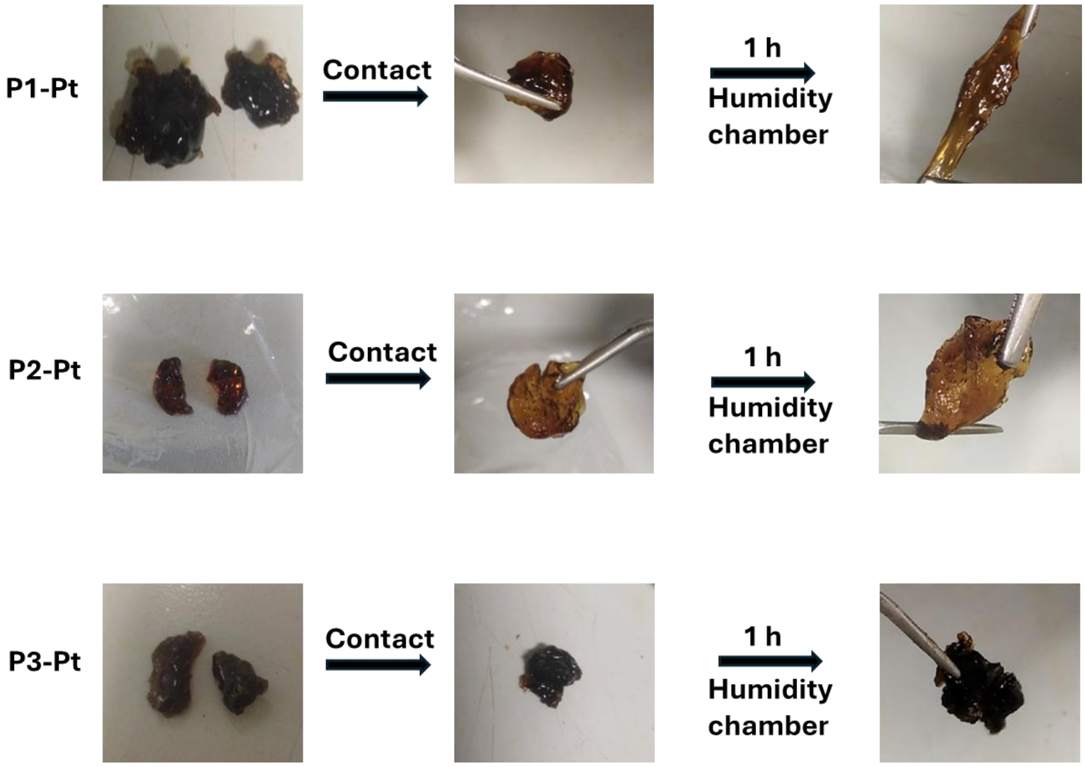

Figure S46. Macroscopic demonstration of self-healing properties of **P1-Pt**, **P2-Pt**, and **P3-Pt** hydrogels after cutting and recombination for 1 hour in a humidity chamber at 25 °C.

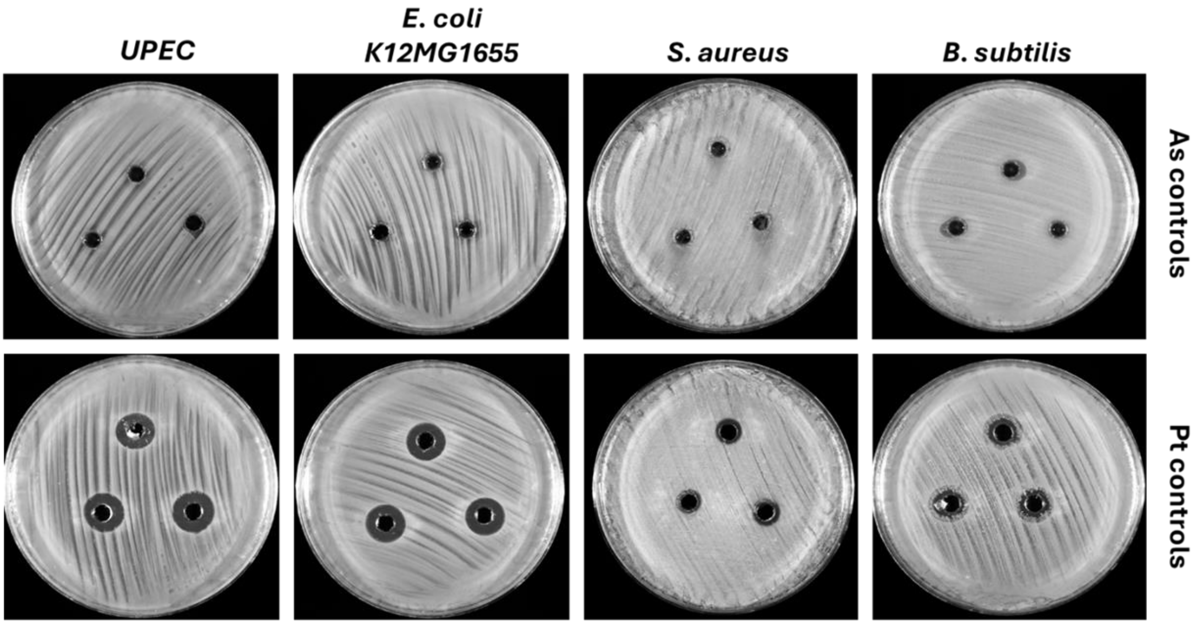

Figure S47. Antibiotic diffusion assay for As, and Pt controls against *UPEC*, *E. coli K12MG1655*, *S. aureus* and *B. subtilis*. *As controls*: **P4** polymer scaffold aqueous solution at a concentration of 100 mg/mL. *Pt controls*: Platinum (Pt^{II}) aqueous solution of 30 mM K_2PtCl_4.

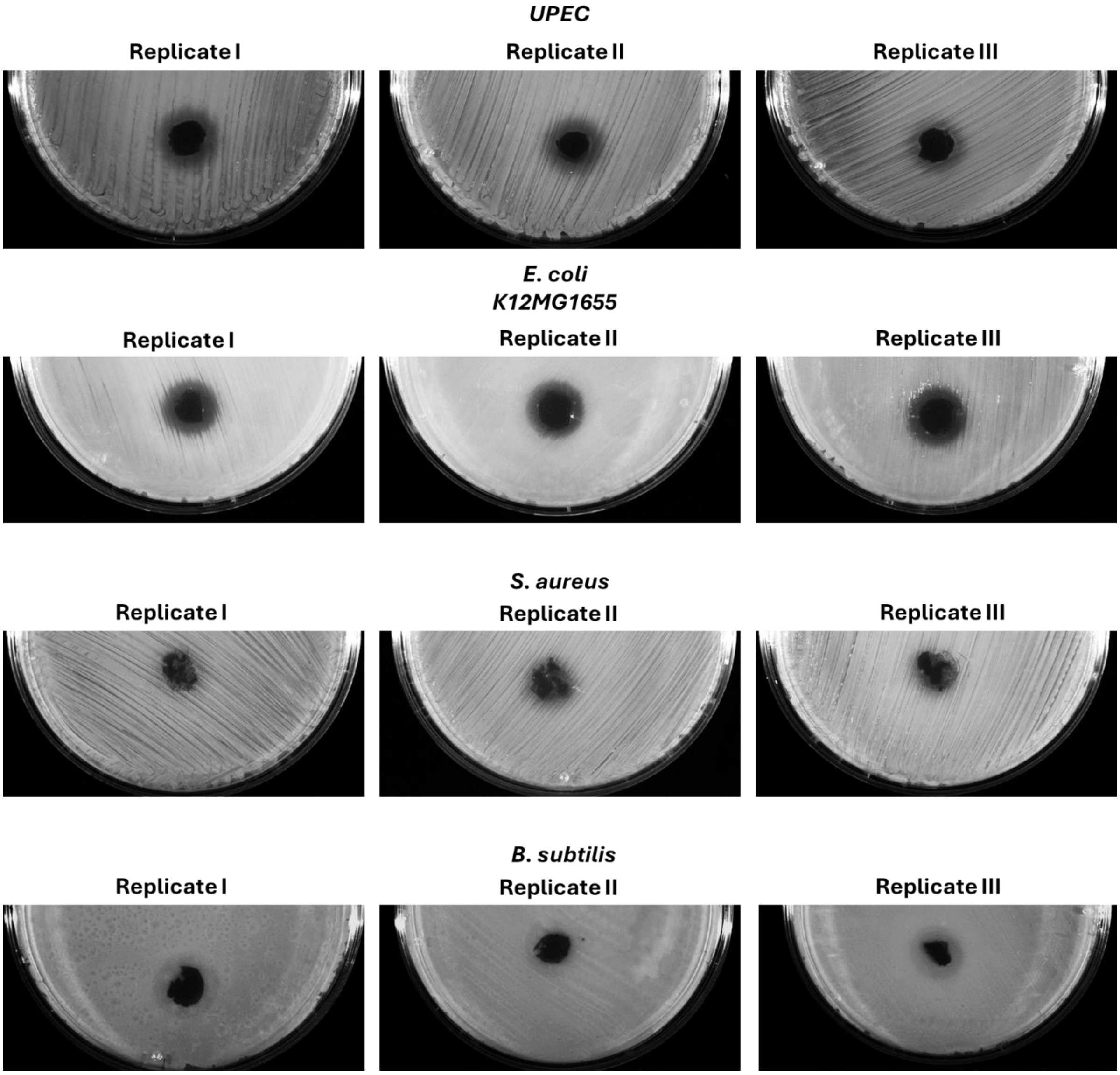

Figure S48. Antibiotic diffusion assay for **P4-Pt** hydrogels against *UPEC, E. coli K12 MG1655, S. aureus* and *B. subtilis.*

References

(1) Tsuchida, E.; Abe, K. Interactions between macromolecules in solution and intermacromolecular complexes. In *Interactions Between Macromolecules in Solution and Intermacromolecular Complexes*, Tsuchida, E., Abe, K. Eds.; Vol. 45; Springer-Verlag: Berlin-Heidelberg, 1982; pp 1-119.

(2) Mandel, M; Leyte, J. C.; Stadhouder, M. The Conformational Transition of Poly (methacrylic acid) in Solution. *J. Phys.Chem.* **1967**, *71* (3), 603-612.

(3) Suzuki, H; Wang, B.; Yoshida, R.; Kokufuta, E. Potentiometric Titration Behaviors of a Polymer and Gel Consisting of N-Isopropylacrylamide and Acrylic Acid. *Langmuir* **1999**, *15* (12), 4283-4288.

(4) Porasso, R. D.; Benegas, J. C.; Van den Hoop, M. A. G. T. Chemical and Electrostatic Association of Various Metal Ions by Poly(acrylic acid) and Poly(methacrylic acid) As Studied by Potentiometry. *J. Phys. Chem. B* **1999**, *103* (13), 2361-2365.

(5) Spitnik, P.; Katchalsky; A. Potentiometric Behavior of Polymethacrylic Acid. *J. Polym. Sci.* **1947**, *2* (4), 432-446.

(6) Katchalsky, A. Solutions of polyelectrolytes and mechanochemical systems. *J. Polym. Sci.* **1951**, *7* (4), 393-412.

(7) De Stefano, C.; Gianguzza, A.; Piazzese, D.; Sammartano, S. Polyacrylates in aqueous solution. The dependence of protonation on molecular weight, ionic medium and ionic strength. *React. Funct. Polym.* **2003**, *55* (1), 9-20.

(8) El Brahmi, K.; Rawiso, M.; François, J. Potentiometric titration of acrylamide - acrlic acid copolymers: Influence of the concentration. *Eur. Polym. J.* **1993**, *29* (12), 1531-1537.

(9) Petrov, A. I.; Antipov, A. A.; Sukhorukov; G. B. Base-Acid Equilibria in Polyelectrolyte Systems: From Weak Polyelectrolytes to Interpolyelectrolyte Complexes and Multilayered Polyelectrolyte Shells. *Macromolecules* **2003**, *36* (26), 10079-10086.

(10) Bjerrum, J. *Metal ammine formation in aqueous solution: theory of the reversible step reactions*; P. Haase: Copenhagen, 1957.

(11) Gregor, H. P.; Luttinger L. B.; Loebl, E. M. Metal-polyelectrolyte complexes. I. The polyacrylic acid-copper complex. *J. Phys. Chem.* **1955**, *59* (1), 34–39.

(12) Mandel, M.; Leyte, J. C. Interaction of Poly(methacrylic Acid) and Bivalent Counterions. I. *J. Polym. Sci. A Gen. Pap.* **1964**, *2* (6), 2883-2899.
